# Hyperbaric oxygen therapy induces transcriptome changes in elderly: a prospective trial

**DOI:** 10.18632/aging.203709

**Published:** 2021-11-24

**Authors:** Amir Hadanny, Relly Forer, Dina Volodarsky, Malka Daniel-Kotovsky, Merav Catalogna, Yonatan Zemel, Yair Bechor, Shai Efrati

**Affiliations:** 1 The Sagol Center for Hyperbaric Medicine and Research, Shamir (Assaf-Harofeh) Medical Center, Zerifin, Israel; 2 Sackler School of Medicine, Tel-Aviv University, Tel-Aviv, Israel; 3 Bar Ilan University, Ramat-Gan, Israel; 4 Aviv Scientific LTD, Bnei-Brak, Israel; 5 Dyn Diagnostics, Zerifin, Israel; 6 Sagol School of Neuroscience, Tel-Aviv University, Tel-Aviv, Israel

**Keywords:** transcriptome, aging, hyperbaric oxygen, HBOT, RNA

## Abstract

Introduction: Aging is characterized by the progressive loss of physiological capacity. Changes in gene expression can alter activity in defined age-related molecular pathways leading to cellular aging and increased aging disease susceptibility. The aim of the current study was to evaluate whether hyperbaric oxygen therapy (HBOT) affects gene expression in normal, non-pathological, aging adults.

Methods: Thirty-five healthy independently living adults, aged 64 and older, were enrolled to receive 60 daily HBOT exposures. Whole blood samples were collected at baseline, at the 30th and 60th HBOT session, and 1–2 weeks following the last session. Differential gene expression analysis was performed.

Results: Following 60 sessions of HBOT, 1342 genes and 570 genes were differently up- and downregulated (1912 total), respectively (*p* < 0.01 FDR), compared to baseline. Out of which, five genes were downregulated with a >1.5-fold change: ABCA13 (FC = −2.28), DNAJ6 (FC = −2.16), HBG2 (FC = −1.56), PDXDC1 (FC = −1.53), RANBP17 (FC = −1.75). Two weeks post-HBOT, ABCA13 expression was significantly downregulated with a >1.5fold change (FC = −1.54, *p* = 0.008).

In conclusion, for the first time in humans, the study provides direct evidence of HBOT is associated with transcriptome changes in whole-blood samples. Our results demonstrate significant changes in gene expression of normal aging population.

## INTRODUCTION

Aging can be defined by the gradual decline of physiological capacities, resulting in impaired functions and susceptibility for diseases and death. This biological deterioration is considered a major risk factor for Alzheimer’s disease, cardiovascular disease, diabetes mellitus, cancer among others. The aging rate is partially determined, by genetics and other evolutionary conserved biochemical processes [1].

Gene expression (whole transcriptome) interrogation is widely used to explore differences between individual young and old, and diseased and healthy populations [2–4]. Significant transcriptional changes related to aging have been suggested with respect to inflammation, oxidative stress, mitochondrial and lysosomal degradation pathways associated genes [3]. Moreover, these gene expression changes can alter activity in defined age-related molecular pathways leading to cellular aging and increased susceptibility to aging diseases.

Hyperbaric oxygen therapy (HBOT) utilizes pure oxygen (100%) at high barometric pressure (>at least over one absolute atmospheres (ATA)) to enhance the body’s tissues oxygen content. Specific HBOT protocols which utilize repeated intermittent hyperoxic exposures induce physiological effects which are classically associated with hypoxia, only using the hyperoxic environment instead. This has been previously referred as the hyperoxic-hypoxic paradox [5–8]. Clinically, HBOT has been used for non-healing ischemic wounds and post-radiation injuries by promoting angiogenesis and wound healing [9]. In our previous study, our group provided evidence HBOT HBOT can induce significant cognitive improvement in the healthy aging population, mediated by cerebral blood flow changes [10]. This indicates that HBOT’s pleiotropic regenerative effects are mediated by activating various genetic pathways. Previous studies evaluated HBOT’s effects on isolated cell cultures and identified different genes sensitive to pressure and oxygen changes including inflammatory, growth, repair, angiogenesis, tumor suppressors, stress, cellular stress and apoptosis associated genes [11–14]. So far, HBOT effects on gene expression *in-vivo* has yet to be explored in clinical studies.

The aim of the current study was to evaluate whether HBOT affects gene expression in the normal aging population (excluding pathological aging).

## METHODS

### Subjects

Thirty-five aging independent adults good functional and cognitive status over 64 years old were enrolled. The clinical study was performed in the Shamir (Assaf-Harofeh) Medical Center, Israel between 2016–2020. All included participants ruled out any history of cardiac or cerebrovascular ischemia for at least one year prior to inclusion. Patients with previous HBOT exposure in the last three months prior to inclusion, known malignancy in the year prior to inclusion, pathological cognitive decline, uncontrolled diabetes mellitus (HbA1C>8, fasting glucose>200), severe chronic renal failure (GFR <30), immunosuppressants, MRI contraindications or active smoking or pulmonary diseases were excluded.

### Study design

The study protocol was approved by the Shamir Medical Center’s Institutional Review Board. All patients signed an informed consent followed by baseline evaluations, HBOT protocol and post therapy evaluations. Measurement points were evaluated at baseline, 2nd baseline control following 60–100 days, last (60th) HBOT session and 7–14 days following the last HBOT session.

The study cohort included only HBOT treated patients, who are part of a larger cohort of normal ageing population studied at the Shamir Medical Center, Israel (NCT02790541 [10]).

### Interventions

The HBOT protocol was provided using a multiplace chamber (Starmed-2700, HAUX, Germany). The protocol included 60 daily sessions, during a three-month period with five sessions per week. Each session comprised of 100% oxygen provided by mask at 2ATA for 90 minutes and intermittent air breaks for five minutes every 20 minutes of oxygen. Compression and decompression rates were controlled at 1 meter/minute. During the study, lifestyle and diet changes and medication adjustments were not allowed.

### Blood samples

Whole blood samples were collected into Tempus™ blood RNA tubes containing a stabilizing reagent using a standard technique following overnight fasting, at baseline, at the second control following 60–100 days, the day of the last HBOT session (60th session) and 10–14 days following HBOT protocol.

### RNA extraction

RNA was extracted using a Tempus™ Spin RNA Isolation Kit (LifeTechnologies, Warrington, UK). Spectrophotometer (NanoDrop 1000, Thermo Fisher Scientific, Waltham, MA, United States) was used to quantify total RNA samples followed by integrity confirmation with the Agilent Bioanalyzer 2100 with an RNA Nano-Chip Kit (Agilent Technologies, Waldbronn, Germany). One hundred nanograms of RNA were used for the Clariom S assay (Affymetrix) following manufacturer’s standardized protocol. The Clariom assay interrogates over 22,000 annotated genes, each consisting of 6–10 probes exactly matching the target transcript sequence.

In brief, 100 ng total RNA and dNTP-T7 random primers were used for reverse transcription (RT). Amplified cRNA was made using Second strand synthesis and *in vitro* transcription amplified cRNA. Subsequent reverse transcription produced cDNA, followed by fragmentation. Fragments were labeled with biotin for hybridization to the replication and transcription activator (RTA). Array processing was done using an Affymetrix GeneChip^®^ Hybridization, Wash, and Stain kit. Initially, 5.2 μg biotin-labeled fragmented ss-cDNA was added to 1× hybridization buffer containing oligo B2, hybridization controls, and DMSO. Following incubation at 95°C and 45°C for 5 minutes each, 200 μL hybridization mix was loaded onto each array. Arrays were incubated for 16th in a GeneChip 645 hybridization oven (Affymetrix) at 45°C while rotating at 60 rpm. Wash/stain procedures were performed on an Affymetrix 450 Fluidics Station using manufacturer specified instrument protocol settings. To remove the unbound sample, arrays were washed with non-stringent wash-Buffer A. GeneChips^®^ were then stained 10 min in Stain Cocktail 1. Excess stain was removed by a subsequent wash in Buffer A. Arrays were incubated 10 min with Stain Cocktail 2 followed by 10 min incubation with Stain Cocktail 1 for signal amplification. GeneChips were washed with Buffer B then filled with Array Holding Buffer prior to removal from the fluidics station and scanned using the GeneArray^®^ 3000 scanner (see Affymetrix target hybridization protocol).

### Transcriptome analysis

The Affymetrix Transcriptome Analysis Console software (TAC) ver. 4.0.1.36 (ThermoFisher Scientific) was used for primary data analysis. Data were normalized using the TAC Robust Multiarray Average module. The Limma Bioconductor package was used for differential expression analysis using an empirical Bayesian correction approach. Gene expression data were log-transformed.

Both baseline measurements were combined as one factor to exclude non-HBOT related changes. A repeated measures one-way ANOVA was used to compare differentially expressed genes (DEG) FC between the 60th session measurement and baseline, post-treatment measurement and baseline. Then a Benjamini-Hochberg method [15] was applied on the calculated *p*-values to correct for false discovery rate. A change was considered significant if met the criterion *P* < 0.01 following a false discovery rate correction.

Functional terms were extracted using the Gene Ontology (GO) terms and Kyoto Encyclopedia of Genes and Genomes (KEGG) pathways. The Expander software was used for both GO and KEGG pathway enrichment analyses [16]. False discovery rate (FDR) below 0.05 threshold was used for statistically significant KEGG pathways and GO terms.

### Quantitative real-time PCR (qPCR)

In order to confirm microarray results RT qPCR reactions were performed. The RT reaction process of mRNA was done with oligo dT primers. The SYBR Green PCR kit (Toyobo, Osaka, Japan) was used to perform qPCR on LightCycler480 real-time PCR system (Roche Diagnostics). HPRT and ABCA13 were the main reference targets of mRNA. The following primers were used: HPRT forward 5′ TTGTTGTAGGATATGCCCTTGA 3′ and HPRT reverse 5′ GCGATGTCAATAGGACTCCAG 3′, ABCA13 forward 5′ GCTTTCTGTATCCTAGTTCTTCTGT 3′ and ABCA13 rev 5′ GATGTACTCTCGCCTCCTAGAT 3′.

### Statistical analysis

Unless otherwise specified (see above for transcriptome analysis), demographic continuous data are expressed as means ± standard-deviation. The Kolmogorov-Smirnov test was used to determine normal distribution for all variables.

Demographics categorical data are expressed in numbers and percentages. Univariate analyses were performed using either chi-square or Fisher’s exact tests, as appropriate. Correlation was performed using Pearson’s correlation coefficient.

## RESULTS

**Figure 1:**
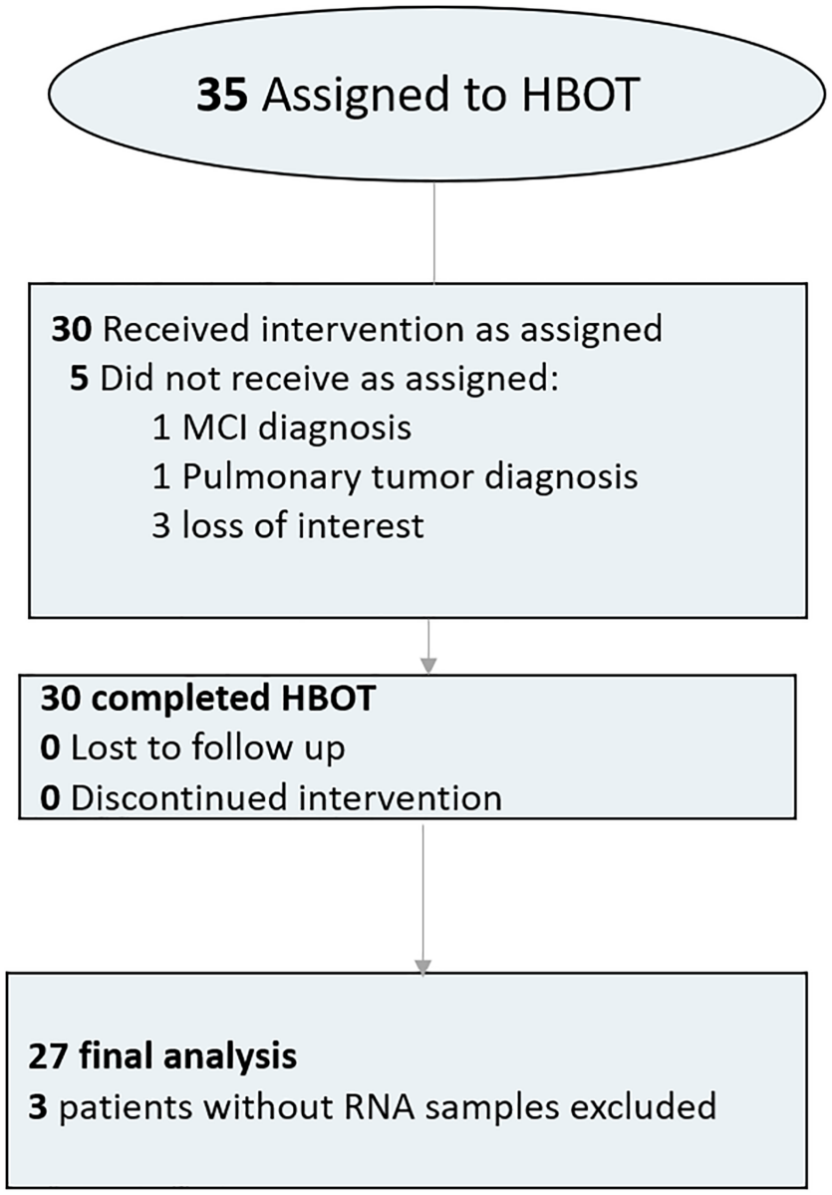
Patient flowchart.

Thirty-five individuals were assigned to HBOT. Five patients were excluded since they did not complete baseline assessments. All other 30 patients completed baseline evaluations the HBOT protocol. Three blood samples did not include RNA tubes and were excluded from analysis (Figure 1). The average age was 70.39 ± 3.72 and 51.9% were males. The baseline characteristics and comparison of the cohorts following exclusion of the patients are in Table 1.

**Table 1:** Baseline characteristics.

**N**		27
**Age (years)**		70.39 ± 3.72
**BMI**		26.55 ± 3.06
**Males**		14 (51.9%)
**Complete blood count**		
	Hemoglobin	13.98 ± 1.39
	White blood cells	6.48 ± 1.22
	Platelets	245.05 ± 45.4
**Chronic medical conditions**		
	Atrial fibrillation	4 (14.8%)
	Hypothyroidism	4 (14.8%)
	Obstructive sleep apnea	3 (11.1%)
	Asthma	1 (3.7%)
	BPH	7 (25.9%)
	GERD	3 (11.1%)
	Osteoporosis	5 (18.5%)
	Rheumatic arthritis	0 (0%)
	Osteoarthritis	4 (14.8%)
	Diabetes mellitus	3 (11.1%)
	Hypertension	6 (22.2%)
	Dyslipidemia	14 (51.9%)
	Ischemic heart disease	0 (0%)
	History of smoking	9 (33.3%)
**Chronic medications**		
	Anti-aggregation	6 (22.2%)
	ACE-Inhibitors/ARB blockers	4 (14.8%)
	Beta blockers	4 (14.8%)
	Calcium blockers	2 (7.4%)
	Alpha blockers	5 (18.5%)
	Diuretics	2 (7.4%)
	Statins	8 (29.6%)
	Oral hypoglycemic	1 (3.7%)
	Bisphosphonates	1 (3.7%)
	Proton pump inhibitors	2 (7.4%)
	Hormones	2 (7.4%)
	Benzodiazepines	2 (7.4%)
	SSRI	5 (18.5%)

Abbreviations: BMI: body mass index; BPH: benign prostate hyperplasia; GERD: gastroesophageal reflux disease; ACE: angiotensin converting enzyme; ARB: angiotensin receptor blocker; SSRI: selective serotonin reuptake inhibitor.

**Figure 2:**
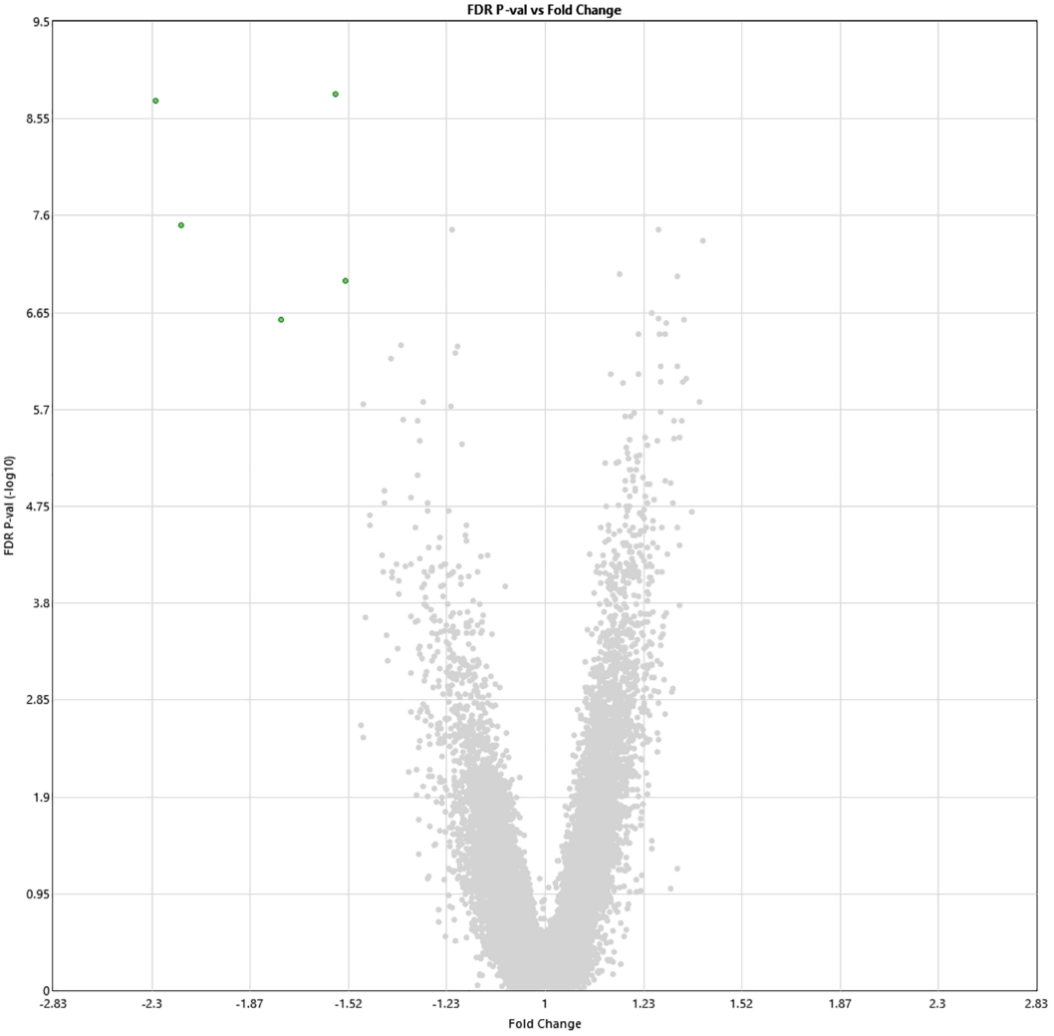
**Alterations in gene expression profile following the last HBOT session.** Volcano plot showing the distribution of gene expression following 60 HBOT sessions compared to baseline. Significance versus log2 fold change is plotted on the y and x, respectively. Red dots represent the significant upregulated DEGs, green dots represent the significant downregulated DEGs.

Following 60 sessions of HBOT, 1342 genes and 570 genes were differently up- and downregulated (1912 total), respectively (*p* < 0.01 FDR) compared to baseline (Figure 2). Out of which, five genes were downregulated with a >1.5-fold change: ABCA13 (FC = −2.28), DNAJ6 (FC = −2.16), HBG2 (FC = −1.56), PDXDC1 (FC = −1.53), RANBP17 (FC = −1.75) (Table 2). The full list of obtained DEGs are in Supplementary Table 1.

**Table 2:** Top DEG following the last HBOT session (fold change>1.5).

**ID**	**Baseline Avg (log2)**	**60th HBOT Avg (log2)**	**Fold Change**	***P*-val**	**FDR *Q*-val**	**Gene Symbol**	**Description**
TC0700007480.hg.1	8.39	7.2	–2.28	1.73E-13	1.86E-09	ABCA13	ATP Binding Cassette Subfamily A Member 13
TC0100008620.hg.1	11.85	10.74	–2.16	4.4E-12	3.15E-08	DNAJC6	DnaJ (Hsp40) homolog, subfamily C, member 6
TC0500009411.hg.1	8.67	7.86	–1.75	1.57E-10	2.60E-07	RANBP17	RAN binding protein 17
TC1100013133.hg.1	15.56	14.92	–1.56	7/62E-14	1.63E-09	HBG2; HBG1	hemoglobin, gamma G; hemoglobin, gamma A
TC1600007007.hg.1	12.09	11.48	–1.53	4.55E-11	1.08E-07	PDXDC1	pyridoxal-dependent decarboxylase domain containing 1

**Figure 3:**
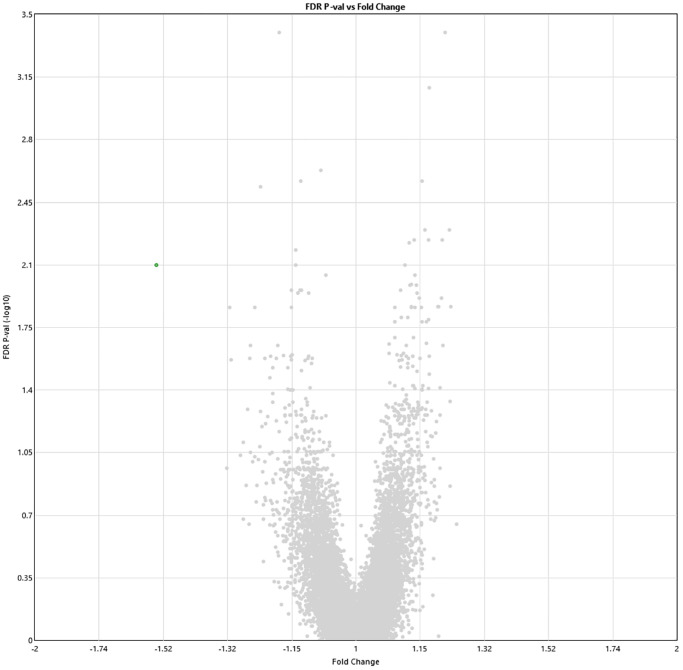
**Alterations in gene expression profile two weeks after completion of 60 HBOT sessions.** Volcano plot showing the distribution of gene expression two weeks following 60 HBOT sessions compared to baseline. Significance versus log2 fold change is plotted on the y and x axes, respectively. Red dots represent the significant upregulated DEGs, green dots represent the significant downregulated DEGs.

Two weeks post-HBOT, 11 genes and 8 genes were differently up- and downregulated (19 total), respectively (*p* < 0.01, FDR) compared to baseline (Figure 3). Out of which, ABCA13 expression was significantly downregulated with a >1.5-fold change (FC = −1.54, *p* = 0.008). The full list of obtained DEGs are in Supplementary Table 2.

The overlap of the two timepoints (Last HBOT session vs baseline, and two weeks post last HBOT sessions vs baseline) consisted of nine differentially expressed genes (DEGs), including *ABCA13*, *RNF13*, *BOC, ARL2*, *MRPS52*, *ATP5J2*, *C20orf27*, *SYNGAP1*, *RHOBTB2.* However the only gene with more than 1.5-fold change was ABCA13 (Table 3).

**Table 3:** Overlapping differentially expressed genes following the last session and two weeks post-HBOT.

**ID**	**Baseline Avg (log2)**	**60th HBOT Avg (log2)**	**2 weeks post HBOT Avg (log2)**	**60th session Fold Change**	**2 weeks post HBOT Fold Change**	***P*-val**	***Q*-val (FDR)**	**Gene Symbol**	**Description**
TC0700007480.hg.1	8.39	7.2	7.76	–2.28	–1.54	1.73e-13	1.86E-09	ABCA13	ATP Binding Cassette Subfamily A Member 13

**Figure 4:**
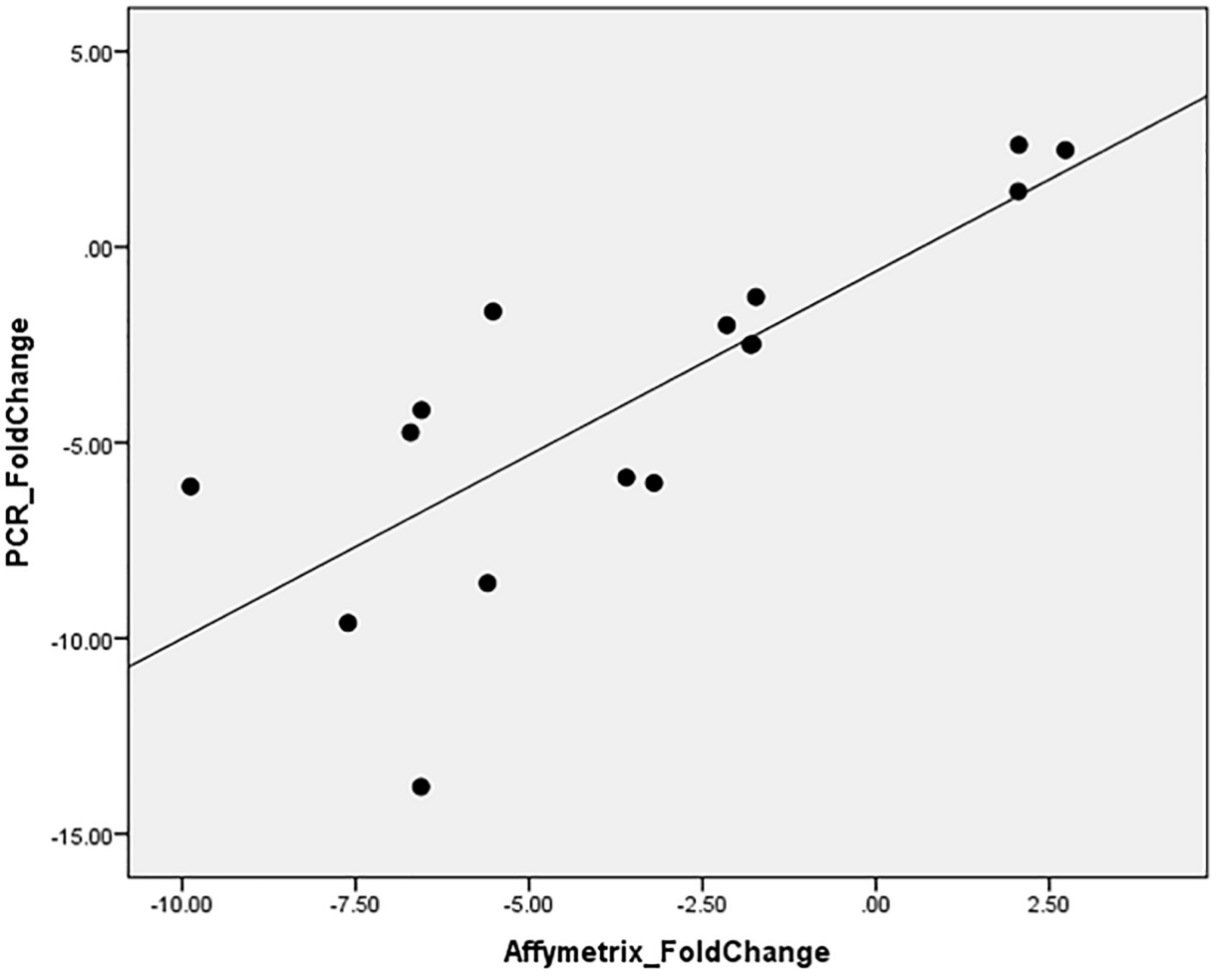
ABCA13 gene expression validation with PCR.

To validate this finding, RT-qPCR was performed for ABCA13 gene on all available samples (16/27) with an average fold change of −3.89 ± 4.45, where in 12/16 the absolute fold change was higher than 1.5. The correlation between RT-qPCR-based fold change and microarray-based fold change of ABCA13 was r = 0.782, *p* < 0.0001 (Figure 4).

Due to the low number of significant DEGs (<10), functional enrichment and pathway enrichment could not be completed.

## DISCUSSION

This longitudinal study of HBOT in healthy aging subjects demonstrates that HBOT strongly associated with blood cells gene expression profiles, as estimated by RNA microarrays. To the best of our knowledge, this serves as the first study assessing human *in vivo* HBOT-mediated changes in RNA expression. Most of the significant changes occurred during the HBOT program and normalized two weeks post-HBOT. However, 19 genes were significantly different even two weeks after the last HBOT session.

Multiple previous studies have demonstrated the effects of HBOT on single gene products in isolated cells [17–25]. Recently, gene array analyses have demonstrated differential gene expression of isolated cells exposed to either single hyperoxic exposure and/or high atmospheric pressure exposure [11–14]. Upregulated genes included anti-inflammatory and growth and repair hormones genes, while downregulated genes included apoptosis and pro-inflammation genes. However, isolated cell studies have significant disadvantages. First, *in vitro* oxygen diffusion is considerably different than *in vivo* diffusion. Obtaining rapid changes in PO_2_ within the cells/tissue culture medium proves to be difficult and limits a precise correlation between cellular processes/events and in environmental oxygen concentration changes [26, 27]. Second, the oxygen partial pressure that cells are truly exposed *in vitro* is significantly different than what is used in *in vivo* studies [28, 29]. Third, *in vitro* studies lack the microsystem interactions which occur *in vivo*. Fourth, gene expression was evaluated following a single hyperoxic exposure. The current clinical study, provides DEG, for the first time in humans, following 60 repeated hyperoxic exposures, both within 24 hours and two weeks after the last exposure.

HBOT is a well-established treatment modality for non-healing wounds, radiation injuries as well as different hypoxic or ischemic events (such as carbon monoxide toxicity, infections, etc.). In recent years, a growing body of evidence from preclinical as well as clinical trials demonstrates HBOT’s efficacy for neurological indications including post-stroke and post-traumatic brain injury [30–36], idiopathic sudden sensorineural hearing loss [37] central sensitization syndromes such as fibromyalgia [38, 39], age related cognitive decline [10] and animal models of Alzheimer’s disease [40]. For the first time, the current study aimed to evaluate the effects of HBOT on the transcriptome of aging humans without any functional limiting diseases.

With regards to ageing, Peters et al. identified 1497 genes that are differentially expressed as we chronologically age [2], out of which, 240 (16%) were differentially expressed in our study following the last HBOT session. For instance, polymorphism in the FOXO3 gene, which encodes forkhead box O-3 transcription factor has been associated with longevity in different human populations [41–43]. In the current study, FOXO3 gene expression changed by 1.22. RUNX3, a hematopoietic stem and progenitor cell factor whose levels decline with aging [44] increased by 1.29. It is important to note that these age genetic signatures generated by different groups show relatively little overlap with each other.

Whole blood transcriptomes are dynamic per definition and represent the cellular state at a certain point. Therefore, it is expected that most of the DEGs occurred during the repeated intermittent hyperbaric exposures, and gradually returned to their previous state two weeks following the last exposure. Previous studies have shown long-term systemic and cellular effects of HBOT, including angiogenesis, stem cells proliferation and mobilization and mitochondrial biogenesis [45] which may be partially explained by these DEGs. The effects last in the protein, tissue and system level compared with the temporal transcription level. ABCA13 is a member of the ATP binding cassette gene subfamily A (ABCA). High expression of ABCA13 has been demonstrated in prostate cancer, leukemia colorectal cancer, as well as several tumor cell lines in central nervous system [46–48]. Additionally, both schizophrenia and bipolar disorder were associated with ABCA13 high expression [48]. Interestingly, expression increases following an ischemic stroke. Lastly, ABCA13 has been shown to decrease significantly in bone-marrow derived mesenchymal stem cells [49]. It has been previously shown HBOT can induce significant recruitment and migration of bone-marrow derived stem cells, both hematopoietic [50] and mesenchymal types [51], which may partially explain the significant down regulation of ABCA13. ABCA13 was the single DEG which remained significant even two weeks following the last HBOT sessions.

The main limitations of the study are related to the lack of a placebo group, required for definitive causality of the observed transcriptome changes, and the relatively small sample size. Additionally, further study with longer observation time would shed better light on additional factors such as subject-specific expression levels differences and their relation to treatment response. These limitations are partially mitigated by the longitudinal design of the study with both the first and second baseline samples from each individual being combined to detect major changes associated with HBOT, rather than temporal/retest effects.

## CONCLUSIONS

For the first time in humans, the study provides direct evidence of HBOT is associated with transcriptome changes in whole blood samples. Our results have demonstrated significant changes in specific gene expressions of normal aging adults.

## Supplementary Materials

Supplementary Table 1

Supplementary Table 2
