# Supplementary material for: Hyperbaric oxygen therapy induces transcriptome changes in elderly: a prospective trial
Source: Aging (Albany NY). 2021 Nov 24;13(22):24511–23. doi: 10.18632/aging.203709 (PMC8660606; doi:10.18632/aging.203709)
Supplement: Supplementary Table 1 [file aging-13-203709-s001.docx]

**Supplementary Table 1: List of differentially expressed genes following the last HBOT session.**

| **ID** | **Baseline Avg (log2)** | **60th HBOT Avg (log2)** | **Fold Change** | **FDR P-val** | **Gene Symbol** |
| --- | --- | --- | --- | --- | --- |
| TC0700007480.hg.1 | 8.39 | 7.2 | -2.28 | 1.86E-09 | ABCA13 |
| TC0100008620.hg.1 | 11.85 | 10.74 | -2.16 | 3.15E-08 | DNAJC6 |
| TC0500009411.hg.1 | 8.67 | 7.86 | -1.75 | 2.60E-07 | RANBP17 |
| TC1100013133.hg.1 | 15.56 | 14.92 | -1.56 | 1.63E-09 | HBG2; HBG1 |
| TC1600007007.hg.1 | 12.09 | 11.48 | -1.53 | 1.08E-07 | PDXDC1 |
| TC2000009550.hg.1 | 10.65 | 10.1 | -1.47 | 1.79E-06 | AURKA |
| TC0100015784.hg.1 | 10.92 | 10.36 | -1.47 | 0.0025 | SELENBP1 |
| TC0400012018.hg.1 | 10.71 | 10.15 | -1.47 | 0.0033 | GYPB |
| TC1400010775.hg.1 | 7.18 | 6.63 | -1.46 | 0.0002 | EFCAB11 |
| TC0600009080.hg.1 | 7.92 | 7.38 | -1.45 | 2.15E-05 | CEP57L1 |
| TC1000011713.hg.1 | 8.95 | 8.41 | -1.45 | 2.73E-05 | PCGF6 |
| TC1700011364.hg.1 | 6.72 | 6.23 | -1.41 | 1.26E-05 | BRIP1 |
| TC0200012742.hg.1 | 12.11 | 11.61 | -1.41 | 5.39E-05 | XPO1 |
| TC1500008023.hg.1 | 12.44 | 11.95 | -1.41 | 7.79E-05 | ZFAND6 |
| TC1500007318.hg.1 | 6.19 | 5.7 | -1.4 | 1.63E-05 | TEX9 |
| TC2100007465.hg.1 | 11.46 | 10.98 | -1.4 | 0.0003 | PCNT |
| TC1400009481.hg.1 | 9.47 | 8.99 | -1.4 | 0.0006 | PLEK2 |
| TC0200016607.hg.1 | 10.9 | 10.43 | -1.39 | 6.32E-07 | UBE2F |
| TC1100010207.hg.1 | 10.95 | 10.48 | -1.38 | 7.81E-05 | SOX6; MIR6073 |
| TC0400007495.hg.1 | 8.61 | 8.14 | -1.38 | 8.82E-05 | DCUN1D4 |
| TC1600007541.hg.1 | 7.32 | 6.87 | -1.37 | 6.59E-05 | AHSP |
| TC1200007906.hg.1 | 7.68 | 7.23 | -1.37 | 0.0004 | XRCC6BP1 |
| TC0300013684.hg.1 | 10.79 | 10.35 | -1.36 | 4.63E-07 | TFRC |
| TC0500008830.hg.1 | 13.24 | 12.79 | -1.36 | 9.53E-05 | UBE2D2 |
| TC0700007198.hg.1 | 8.18 | 7.74 | -1.36 | 0.0001 | ANLN |
| TC1300010030.hg.1 | 14.16 | 13.73 | -1.35 | 2.53E-06 | N4BP2L2 |
| TC1100010643.hg.1 | 7.72 | 7.3 | -1.34 | 6.83E-05 | TP53I11 |
| TC0200014154.hg.1 | 8.34 | 7.93 | -1.34 | 0.0071 | GYPC |
| TC1300008280.hg.1 | 10.82 | 10.41 | -1.33 | 1.46E-05 | SKA3 |
| TC1400007201.hg.1 | 5.82 | 5.41 | -1.33 | 6.63E-05 | CDKN3 |
| TC0400012015.hg.1 | 11.46 | 11.06 | -1.33 | 0.0002 | GYPE |
| TC0100017084.hg.1 | 9.43 | 9.02 | -1.33 | 0.0008 | CTSE |
| TC1500010857.hg.1 | 12.65 | 12.24 | -1.33 | 0.0019 | EPB42 |
| TC1500010856.hg.1 | 10.86 | 10.46 | -1.32 | 0.0002 | EPB42 |
| TC0200010966.hg.1 | 12.58 | 12.19 | -1.31 | 2.58E-06 | RHBDD1 |
| TSUnmapped00000627.hg.1 | 5.73 | 5.34 | -1.31 | 8.83E-06 | KIF15 |
| TC0200016581.hg.1 | 9.51 | 9.12 | -1.31 | 2.84E-05 | C2orf88 |
| TC0200015194.hg.1 | 7.54 | 7.15 | -1.31 | 0.0002 | TFPI |
| TC1000011516.hg.1 | 8.17 | 7.79 | -1.31 | 0.0004 | BLNK |
| TC0900006559.hg.1 | 7.37 | 6.98 | -1.31 | 0.0021 | CD274 |
| TC1700008123.hg.1 | 9.88 | 9.49 | -1.31 | 0.0041 | MYL4 |
| TC1800007198.hg.1 | 11.37 | 10.98 | -1.31 | 0.0069 | SLC14A1 |
| TC0200015424.hg.1 | 11.79 | 11.41 | -1.3 | 4.00E-06 | ALS2CR12 |
| TC0400011383.hg.1 | 13.94 | 13.56 | -1.3 | 5.79E-05 | TSPAN5 |
| TC0500011712.hg.1 | 8.33 | 7.96 | -1.3 | 0.0001 | EPB41L4A |
| TC1100009431.hg.1 | 6.96 | 6.58 | -1.3 | 0.0004 | PATE4 |
| TC0800010990.hg.1 | 15.51 | 15.13 | -1.3 | 0.0005 | CA1 |
| TC0300013420.hg.1 | 11.43 | 11.05 | -1.3 | 0.0006 | IGF2BP2 |
| TC0700010716.hg.1 | 9.6 | 9.22 | -1.3 | 0.0018 | DPY19L1 |
| TC0900009855.hg.1 | 11.01 | 10.63 | -1.3 | 0.0018 | BAG1 |
| TC1100010044.hg.1 | 4.09 | 3.7 | -1.3 | 0.0035 | ST5 |
| TC0200016454.hg.1 | 10.9 | 10.53 | -1.29 | 1.69E-06 | KIAA1841 |
| TC1300006979.hg.1 | 11.74 | 11.37 | -1.29 | 7.77E-05 | RGCC |
| TC0900011473.hg.1 | 9.4 | 9.03 | -1.29 | 0.0001 | DENND1A |
| TC2000007251.hg.1 | 11.7 | 11.33 | -1.29 | 0.0001 | MYL9 |
| TC0X00010465.hg.1 | 8.81 | 8.44 | -1.29 | 0.0002 | NUP62CL |
| TC2000007817.hg.1 | 11.34 | 10.98 | -1.29 | 0.0002 | FAM210B |
| TC2100008487.hg.1 | 6.64 | 6.28 | -1.29 | 0.0008 | TPTE |
| TC1000008399.hg.1 | 9.23 | 8.87 | -1.29 | 0.0008 | IFIT1B |
| TC0100018531.hg.1 | 9.82 | 9.45 | -1.29 | 0.0009 | PIGC |
| TC0700012959.hg.1 | 8.82 | 8.44 | -1.29 | 0.0016 | EZH2 |
| TC0200008351.hg.1 | 10.59 | 10.22 | -1.29 | 0.0023 | RPIA |
| TC1400008635.hg.1 | 11.32 | 10.95 | -1.29 | 0.01 | RAB2B |
| TC1900012055.hg.1 | 6.23 | 5.87 | -1.28 | 1.63E-05 | VN1R1 |
| TC2000009026.hg.1 | 10.49 | 10.13 | -1.28 | 1.96E-05 | RBL1 |
| TC1000007226.hg.1 | 9.46 | 9.1 | -1.28 | 4.52E-05 | CCDC7 |
| TC1700008186.hg.1 | 7.65 | 7.3 | -1.28 | 8.69E-05 | IGF2BP1 |
| TC0700007031.hg.1 | 11.45 | 11.1 | -1.28 | 0.0004 | JAZF1 |
| TC2000007117.hg.1 | 11.09 | 10.73 | -1.28 | 0.0017 | ASXL1 |
| TC0900008441.hg.1 | 8.29 | 7.93 | -1.28 | 0.0019 | UGCG |
| TC0700008351.hg.1 | 7.69 | 7.34 | -1.28 | 0.0032 | GNG11 |
| TC0X00006957.hg.1 | 9.57 | 9.22 | -1.28 | 0.0039 | XK |
| TC0100012351.hg.1 | 4.47 | 4.12 | -1.28 | 0.0068 | OR2M3 |
| TC0100012329.hg.1 | 11.56 | 11.2 | -1.28 | 0.0079 | OR2W3 |
| TC1200007137.hg.1 | 11.55 | 11.2 | -1.27 | 6.99E-05 | FGFR1OP2 |
| TC1300009505.hg.1 | 9.16 | 8.81 | -1.27 | 7.53E-05 | ABCC4 |
| TC1300008511.hg.1 | 7.45 | 7.1 | -1.27 | 7.65E-05 | KATNAL1 |
| TC1200008669.hg.1 | 7.27 | 6.92 | -1.27 | 0.0002 | TDG |
| TC0700008745.hg.1 | 11.29 | 10.95 | -1.27 | 0.0002 | PRKAR2B |
| TC1000009851.hg.1 | 7.87 | 7.53 | -1.27 | 0.0002 | FRMD4A |
| TC2100006854.hg.1 | 10.2 | 9.85 | -1.27 | 0.0003 | MAP3K7CL |
| TC0400012019.hg.1 | 11.4 | 11.05 | -1.27 | 0.0006 | GYPA |
| TC1200012725.hg.1 | 6.04 | 5.7 | -1.27 | 0.0011 | FAM101A |
| TC0600008272.hg.1 | 11.38 | 11.03 | -1.27 | 0.002 | FBXO9 |
| TC0700009174.hg.1 | 11.43 | 11.09 | -1.27 | 0.0025 | MKLN1 |
| TC2200007069.hg.1 | 11.52 | 11.18 | -1.27 | 0.0079 | OSBP2 |
| TC0800006969.hg.1 | 12.26 | 11.92 | -1.26 | 0.0002 | DMTN |
| TC0500007350.hg.1 | 6.42 | 6.09 | -1.26 | 0.0003 | ISL1 |
| TC1600011314.hg.1 | 9.69 | 9.36 | -1.26 | 0.0004 | HBM |
| TC1700011785.hg.1 | 9.94 | 9.61 | -1.26 | 0.0005 | EXOC7 |
| TC1900010708.hg.1 | 13.19 | 12.85 | -1.26 | 0.0005 | BLVRB |
| TC1200012698.hg.1 | 11.56 | 11.24 | -1.26 | 0.0025 | CHPT1 |
| TC1700011033.hg.1 | 14.17 | 13.85 | -1.26 | 0.0027 | PHOSPHO1 |
| TC0500012567.hg.1 | 14.82 | 14.5 | -1.26 | 0.003 | FAXDC2 |
| TC0900009882.hg.1 | 16.77 | 16.44 | -1.25 | 3.60E-05 | DCAF12 |
| TC0200009189.hg.1 | 15.89 | 15.57 | -1.25 | 4.46E-05 | GYPC |
| TC0100017713.hg.1 | 10.58 | 10.26 | -1.25 | 6.83E-05 | TTC13 |
| TC1500007035.hg.1 | 8.83 | 8.51 | -1.25 | 0.0001 | HAUS2 |
| TC0800010252.hg.1 | 12.01 | 11.69 | -1.25 | 0.0002 | ANK1 |
| TC0800010458.hg.1 | 11.89 | 11.57 | -1.25 | 0.0003 | TCEA1 |
| TC1000008431.hg.1 | 14.66 | 14.34 | -1.25 | 0.0007 | PCGF5 |
| TC0X00009486.hg.1 | 4.86 | 4.54 | -1.25 | 0.0009 | MAOB |
| TC1000006861.hg.1 | 5.59 | 5.26 | -1.25 | 0.001 | MEIG1 |
| TC1100009301.hg.1 | 12.39 | 12.07 | -1.25 | 0.0024 | TBCEL |
| TC0X00007176.hg.1 | 4.36 | 4.04 | -1.25 | 0.0027 | SSX1 |
| TC1300009088.hg.1 | 7.7 | 7.38 | -1.25 | 0.0034 | DIAPH3 |
| TC1600008209.hg.1 | 6.26 | 5.94 | -1.25 | 0.0038 | CDH1 |
| TC0100011325.hg.1 | 8.53 | 8.2 | -1.25 | 0.0081 | TMCC2 |
| TC0X00008794.hg.1 | 12.92 | 12.6 | -1.25 | 0.0089 | SLC6A8 |
| TC0500009598.hg.1 | 7.91 | 7.61 | -1.24 | 6.63E-05 | EIF4E1B |
| TC0300010722.hg.1 | 10.26 | 9.95 | -1.24 | 0.0001 | LRRFIP2 |
| TC0100011855.hg.1 | 13.67 | 13.36 | -1.24 | 0.0001 | GUK1 |
| TC1600010792.hg.1 | 8.87 | 8.56 | -1.24 | 0.0003 | PHLPP2 |
| TC0200010448.hg.1 | 17.68 | 17.37 | -1.24 | 0.0012 | STRADB |
| TC0200015425.hg.1 | 12.62 | 12.31 | -1.24 | 0.0021 | TRAK2 |
| TC1800007448.hg.1 | 6.24 | 5.94 | -1.24 | 0.0031 | GRP |
| TC1800008776.hg.1 | 14.57 | 14.25 | -1.24 | 0.0033 | FECH |
| TC1100009896.hg.1 | 4.7 | 4.4 | -1.24 | 0.0036 | OR51S1 |
| TC0300006791.hg.1 | 14.15 | 13.83 | -1.24 | 0.0037 | KAT2B |
| TC1400008098.hg.1 | 14.63 | 14.32 | -1.24 | 0.005 | GLRX5 |
| TC1400007665.hg.1 | 10.81 | 10.5 | -1.24 | 0.0086 | BBOF1 |
| TC1100009287.hg.1 | 11.55 | 11.24 | -1.24 | 0.0089 | ARHGEF12 |
| TC1200008466.hg.1 | 14.62 | 14.32 | -1.23 | 1.96E-05 | NUDT4 |
| TC0100018549.hg.1 | 8.36 | 8.05 | -1.23 | 6.63E-05 | TNNT2 |
| TC0100009572.hg.1 | 16.03 | 15.73 | -1.23 | 8.52E-05 | FAM46C |
| TC0500008882.hg.1 | 9.24 | 8.94 | -1.23 | 0.0002 | PCDHB11 |
| TC1500009804.hg.1 | 14.04 | 13.73 | -1.23 | 0.0003 | DENND4A |
| TC0800011826.hg.1 | 13.69 | 13.39 | -1.23 | 0.0004 | ASAP1; ASAP1-IT2 |
| TC1000008881.hg.1 | 15.75 | 15.46 | -1.23 | 0.0004 | MXI1 |
| TC1300008342.hg.1 | 8.71 | 8.42 | -1.23 | 0.0007 | C1QTNF9B |
| TC1700012195.hg.1 | 5.95 | 5.65 | -1.23 | 0.0007 | TBC1D26 |
| TC1000009930.hg.1 | 7.5 | 7.2 | -1.23 | 0.0007 | HACD1 |
| TC1200011037.hg.1 | 6.2 | 5.9 | -1.23 | 0.001 | FAM19A2 |
| TC0300010962.hg.1 | 5.89 | 5.59 | -1.23 | 0.0015 | MYL3 |
| TC0100015299.hg.1 | 9.75 | 9.45 | -1.23 | 0.0022 | ST7L |
| TC1100010472.hg.1 | 4.68 | 4.37 | -1.23 | 0.0022 | CCDC73 |
| TC0600013273.hg.1 | 7.64 | 7.34 | -1.23 | 0.0032 | BCLAF1 |
| TC0400008053.hg.1 | 16.44 | 16.15 | -1.22 | 3.45E-08 | AFF1 |
| TC0400011056.hg.1 | 12.54 | 12.25 | -1.22 | 1.87E-06 | NUP54 |
| TC0400011313.hg.1 | 16.82 | 16.54 | -1.22 | 7.73E-05 | SNCA |
| TC0800008726.hg.1 | 10.72 | 10.43 | -1.22 | 8.32E-05 | TBC1D31 |
| TC2000007016.hg.1 | 6.74 | 6.46 | -1.22 | 0.0002 | GINS1 |
| TC0200016464.hg.1 | 9.22 | 8.93 | -1.22 | 0.0002 | APLF |
| TC0400011013.hg.1 | 15.59 | 15.29 | -1.22 | 0.0003 | PPBP |
| TC0X00006585.hg.1 | 4.64 | 4.35 | -1.22 | 0.0004 | SHROOM2 |
| TC1500007967.hg.1 | 10.22 | 9.93 | -1.22 | 0.0005 | DNAJA4 |
| TC0700006928.hg.1 | 10.21 | 9.91 | -1.22 | 0.0007 | CCDC126 |
| TC1900006470.hg.1 | 15.82 | 15.53 | -1.22 | 0.0008 | BSG |
| TC0300013330.hg.1 | 13.04 | 12.76 | -1.22 | 0.0008 | DCUN1D1 |
| TC1300006652.hg.1 | 10.51 | 10.22 | -1.22 | 0.0008 | CDK8 |
| TC1600008165.hg.1 | 8.59 | 8.31 | -1.22 | 0.001 | FAM65A |
| TC0100007333.hg.1 | 14.55 | 14.26 | -1.22 | 0.0011 | PITHD1 |
| TC0600007198.hg.1 | 8.59 | 8.3 | -1.22 | 0.0012 | ALDH5A1 |
| TC0900007129.hg.1 | 6.11 | 5.82 | -1.22 | 0.0017 | MELK |
| TC0600009059.hg.1 | 14.62 | 14.33 | -1.22 | 0.0018 | FOXO3 |
| TC0100014081.hg.1 | 11.66 | 11.38 | -1.22 | 0.0027 | PDZK1IP1 |
| TC0100018440.hg.1 | 6.43 | 6.14 | -1.22 | 0.0032 | STIL |
| TC2000009588.hg.1 | 7.86 | 7.57 | -1.22 | 0.004 | MTRNR2L3 |
| TC1900011495.hg.1 | 4.51 | 4.23 | -1.22 | 0.0047 | NLRP11 |
| TC0300010068.hg.1 | 13.51 | 13.23 | -1.21 | 5.59E-07 | LRCH3 |
| TC0200011720.hg.1 | 12.78 | 12.5 | -1.21 | 0.0003 | ODC1; SNORA80B |
| TC0200013095.hg.1 | 9.13 | 8.85 | -1.21 | 0.0003 | BOLA3 |
| TC1600007141.hg.1 | 8.42 | 8.14 | -1.21 | 0.0003 | LOC81691; AC004381.6; U4atac |
| TC1700012120.hg.1 | 9.41 | 9.13 | -1.21 | 0.0003 | OGFOD3 |
| TC1200012620.hg.1 | 6.13 | 5.85 | -1.21 | 0.0005 | SLC48A1 |
| TC0900011959.hg.1 | 8.1 | 7.82 | -1.21 | 0.0006 | UBAC1 |
| TC0900006583.hg.1 | 10 | 9.72 | -1.21 | 0.0006 | UHRF2 |
| TC1000011454.hg.1 | 8.33 | 8.05 | -1.21 | 0.0008 | FRA10AC1 |
| TC1000010507.hg.1 | 6.51 | 6.24 | -1.21 | 0.0008 | buskee; FAM25D; FAM25E; zawskaw |
| TC0X00010408.hg.1 | 6.06 | 5.79 | -1.21 | 0.0008 | TCEAL5 |
| TC0100010192.hg.1 | 9.61 | 9.33 | -1.21 | 0.0009 | RRNAD1 |
| TC0100016930.hg.1 | 4.87 | 4.59 | -1.21 | 0.0009 | LMOD1 |
| TC1600009422.hg.1 | 15.37 | 15.1 | -1.21 | 0.0009 | GSPT1 |
| TC0300013934.hg.1 | 11.24 | 10.96 | -1.21 | 0.0016 | EMC3 |
| TC0X00007204.hg.1 | 10.26 | 9.98 | -1.21 | 0.0018 | GATA1 |
| TC0100017467.hg.1 | 11.9 | 11.62 | -1.21 | 0.0018 | NVL |
| TC0X00010954.hg.1 | 6.23 | 5.96 | -1.21 | 0.0025 | FGF13; LINC00889 |
| TC0200016626.hg.1 | 11.28 | 11 | -1.21 | 0.0031 | MBOAT2 |
| TC0800008126.hg.1 | 11.34 | 11.07 | -1.21 | 0.0031 | CA2 |
| TC0500013215.hg.1 | 5.5 | 5.22 | -1.21 | 0.0035 | LVRN |
| TC2000006571.hg.1 | 8.48 | 8.2 | -1.21 | 0.0036 | SMOX |
| TC0500013244.hg.1 | 5.11 | 4.83 | -1.21 | 0.0044 | PCDHB16 |
| TC0800006488.hg.1 | 8.29 | 8.02 | -1.21 | 0.0056 | ARHGEF10 |
| TC0500009743.hg.1 | 7.75 | 7.48 | -1.21 | 0.007 | BTNL8 |
| TSUnmapped00000174.hg.1 | 6.6 | 6.32 | -1.21 | 0.0081 | BCL2L14 |
| TC0100018439.hg.1 | 9.27 | 8.99 | -1.21 | 0.0088 | TAL1 |
| TC0400007836.hg.1 | 6.96 | 6.69 | -1.21 | 0.0089 | CXCL8 |
| TC0800006624.hg.1 | 3.87 | 3.59 | -1.21 | 0.0096 | DEFB4A |
| TC1400008683.hg.1 | 13.54 | 13.27 | -1.2 | 4.76E-07 | RBM23 |
| TC1700008240.hg.1 | 10.9 | 10.63 | -1.2 | 6.83E-05 | COL1A1 |
| TC0500007465.hg.1 | 11.79 | 11.53 | -1.2 | 0.0001 | GPBP1 |
| TC0400012964.hg.1 | 11.76 | 11.5 | -1.2 | 0.0002 | SCLT1 |
| TC1600006448.hg.1 | 7.77 | 7.5 | -1.2 | 0.0003 | HBQ1 |
| TC1100009819.hg.1 | 11.73 | 11.46 | -1.2 | 0.0003 | NAP1L4 |
| TC1900009287.hg.1 | 7.6 | 7.34 | -1.2 | 0.0005 | SMIM24 |
| TC0800009637.hg.1 | 4.16 | 3.9 | -1.2 | 0.0006 | USP17L2 |
| TC1800008437.hg.1 | 3.95 | 3.68 | -1.2 | 0.0007 | NOL4 |
| TC1400007354.hg.1 | 14.96 | 14.69 | -1.2 | 0.0008 | PPM1A |
| TC1700011262.hg.1 | 5.05 | 4.78 | -1.2 | 0.0012 | C17orf47 |
| TC2000009802.hg.1 | 6.87 | 6.61 | -1.2 | 0.0013 | NKAIN4 |
| TC0Y00006571.hg.1 | 4.13 | 3.87 | -1.2 | 0.0013 | TSPY3 |
| TC2200008411.hg.1 | 13.65 | 13.39 | -1.2 | 0.0016 | ASCC2 |
| TC0400012856.hg.1 | 7.69 | 7.43 | -1.2 | 0.0017 | RAPGEF2 |
| TC1900007876.hg.1 | 6.74 | 6.48 | -1.2 | 0.0019 | ZBTB32 |
| TC1200010265.hg.1 | 10.65 | 10.39 | -1.2 | 0.0021 | CAPRIN2 |
| TC0500012957.hg.1 | 9.83 | 9.57 | -1.2 | 0.0024 | UIMC1 |
| TC0900010623.hg.1 | 11.48 | 11.21 | -1.2 | 0.0025 | ISCA1 |
| TC0900008150.hg.1 | 11.19 | 10.93 | -1.2 | 0.0028 | TMOD1 |
| TC0600008505.hg.1 | 4.43 | 4.17 | -1.2 | 0.0031 | RIMS1 |
| TC1400009108.hg.1 | 6.66 | 6.39 | -1.2 | 0.0031 | POLE2 |
| TC0800012001.hg.1 | 6.66 | 6.39 | -1.2 | 0.0032 | PTK2 |
| TC0800007309.hg.1 | 11.23 | 10.98 | -1.2 | 0.0032 | PROSC |
| TC0X00007556.hg.1 | 10.4 | 10.14 | -1.2 | 0.0033 | FOXO4 |
| TC0100010518.hg.1 | 11.85 | 11.58 | -1.2 | 0.0056 | DCAF6 |
| TC1100008490.hg.1 | 5.94 | 5.68 | -1.2 | 0.0058 | UVRAG |
| TC0900011710.hg.1 | 13.39 | 13.13 | -1.2 | 0.0059 | C9orf78 |
| TC0300012807.hg.1 | 13.8 | 13.54 | -1.2 | 0.0088 | SIAH2 |
| TC1200008642.hg.1 | 6.73 | 6.47 | -1.2 | 0.0093 | PARPBP |
| TC1900008456.hg.1 | 5.18 | 4.91 | -1.2 | 0.0097 | ELSPBP1 |
| TC1700010298.hg.1 | 13.62 | 13.37 | -1.19 | 4.39E-06 | CRLF3 |
| TC0700013485.hg.1 | 7.29 | 7.04 | -1.19 | 8.91E-05 | LOC155060; ZNF783 |
| TC0300012216.hg.1 | 10.54 | 10.29 | -1.19 | 0.0006 | CCDC14 |
| TC0100008955.hg.1 | 13.28 | 13.04 | -1.19 | 0.0006 | PKN2 |
| TC1500009393.hg.1 | 4.57 | 4.32 | -1.19 | 0.0006 | USP50 |
| TC0200016074.hg.1 | 4.75 | 4.5 | -1.19 | 0.0008 | NGEF |
| TC0700011383.hg.1 | 9.49 | 9.24 | -1.19 | 0.0008 | SBDS |
| TC0900007488.hg.1 | 10.03 | 9.78 | -1.19 | 0.001 | PIP5K1B |
| TC0X00007170.hg.1 | 4.49 | 4.24 | -1.19 | 0.0017 | SPACA5B |
| TC0100013556.hg.1 | 4.77 | 4.52 | -1.19 | 0.002 | NKAIN1 |
| TC0X00011223.hg.1 | 14.57 | 14.32 | -1.19 | 0.0021 | MPP1 |
| TC1000008320.hg.1 | 7.06 | 6.81 | -1.19 | 0.0026 | SNCG |
| TC0300012178.hg.1 | 4.55 | 4.29 | -1.19 | 0.0027 | ILDR1 |
| TC0400011014.hg.1 | 8.85 | 8.6 | -1.19 | 0.0031 | CXCL5 |
| TC0X00009795.hg.1 | 17.16 | 16.91 | -1.19 | 0.0031 | ALAS2 |
| TC0500010680.hg.1 | 7.1 | 6.86 | -1.19 | 0.0038 | PARP8 |
| TC1200012080.hg.1 | 11.52 | 11.28 | -1.19 | 0.0038 | TESC |
| TC0100007188.hg.1 | 5.86 | 5.61 | -1.19 | 0.004 | PLA2G5 |
| TC0100008271.hg.1 | 13.64 | 13.39 | -1.19 | 0.0045 | RNF11 |
| TC0600014300.hg.1 | 10.83 | 10.58 | -1.19 | 0.0051 | GCLC |
| TC0200013912.hg.1 | 5.14 | 4.88 | -1.19 | 0.0071 | CKAP2L |
| TC0500012519.hg.1 | 11.3 | 11.05 | -1.19 | 0.0079 | SPARC |
| TC1600009677.hg.1 | 6.47 | 6.23 | -1.19 | 0.0083 | CRYM |
| TC0100011263.hg.1 | 4.29 | 4.05 | -1.19 | 0.0085 | OPTC |
| TC0100010000.hg.1 | 5.34 | 5.08 | -1.19 | 0.009 | LCE2B |
| TC0100010648.hg.1 | 14.15 | 13.89 | -1.19 | 0.0098 | PRDX6 |
| TC1200009913.hg.1 | 16.66 | 16.42 | -1.18 | 2.74E-05 | YBX3 |
| TC0300009147.hg.1 | 14.86 | 14.62 | -1.18 | 3.39E-05 | RNF13 |
| TC1000010022.hg.1 | 16.45 | 16.21 | -1.18 | 3.85E-05 | PIP4K2A |
| TC1000012519.hg.1 | 13.68 | 13.45 | -1.18 | 8.62E-05 | IL15RA |
| TC0400007839.hg.1 | 13.9 | 13.66 | -1.18 | 0.0002 | PF4V1 |
| TC1500009796.hg.1 | 3.96 | 3.72 | -1.18 | 0.0002 | IGDCC4 |
| TC0100010785.hg.1 | 4.03 | 3.79 | -1.18 | 0.0003 | RP11-12M5.1; FAM163A |
| TC1000012011.hg.1 | 13.42 | 13.18 | -1.18 | 0.0003 | RGS10 |
| TC1900011761.hg.1 | 9.21 | 8.97 | -1.18 | 0.0003 | APOC4 |
| TC0100009760.hg.1 | 14.76 | 14.53 | -1.18 | 0.0003 | NUDT4P1; NUDT4P2; NUDT4 |
| TC0100009833.hg.1 | 14.76 | 14.53 | -1.18 | 0.0003 | NUDT4; NUDT4P1 |
| TC1500009127.hg.1 | 3.96 | 3.73 | -1.18 | 0.0004 | PPP1R14D |
| TC0600012314.hg.1 | 4.21 | 3.96 | -1.18 | 0.0006 | COL12A1 |
| TC1600011320.hg.1 | 7.67 | 7.43 | -1.18 | 0.0006 | WFIKKN1 |
| TC1100010535.hg.1 | 4.25 | 4.01 | -1.18 | 0.0007 | PAMR1 |
| TC0400011012.hg.1 | 13.64 | 13.4 | -1.18 | 0.0008 | PF4 |
| TC0200016718.hg.1 | 4.6 | 4.36 | -1.18 | 0.0008 | TBC1D8 |
| TC1600010467.hg.1 | 6.28 | 6.03 | -1.18 | 0.0012 | CNGB1 |
| TC1900007238.hg.1 | 4.52 | 4.29 | -1.18 | 0.0016 | OR10H3 |
| TC1000010497.hg.1 | 15.87 | 15.63 | -1.18 | 0.0016 | 08-מרץ |
| TC1000008516.hg.1 | 4.39 | 4.15 | -1.18 | 0.0018 | ACSM6 |
| TC1900007053.hg.1 | 6.82 | 6.58 | -1.18 | 0.0026 | ZNF69 |
| TC1200009132.hg.1 | 14.1 | 13.87 | -1.18 | 0.0026 | RNF10 |
| TC0100006907.hg.1 | 5.44 | 5.2 | -1.18 | 0.0027 | PRAMEF12 |
| TC0100006682.hg.1 | 6.97 | 6.72 | -1.18 | 0.0027 | LINC00337 |
| TC0900010959.hg.1 | 15.06 | 14.82 | -1.18 | 0.003 | HEMGN |
| TC1900008213.hg.1 | 3.45 | 3.22 | -1.18 | 0.0031 | PRG1 |
| TC0400007776.hg.1 | 4.93 | 4.69 | -1.18 | 0.0032 | FDCSP |
| TC0600014280.hg.1 | 4.12 | 3.88 | -1.18 | 0.0033 | DAXX |
| TC0800011216.hg.1 | 9.17 | 8.93 | -1.18 | 0.0037 | STK3 |
| TC0600008622.hg.1 | 10 | 9.76 | -1.18 | 0.0039 | SH3BGRL2 |
| TC0600013796.hg.1 | 5.99 | 5.75 | -1.18 | 0.004 | AGPAT4 |
| TC0500010928.hg.1 | 7.73 | 7.5 | -1.18 | 0.004 | CENPK |
| TC1100007630.hg.1 | 3.53 | 3.3 | -1.18 | 0.005 | OR5T3 |
| TC0100018556.hg.1 | 5.22 | 4.97 | -1.18 | 0.0054 | BLACAT1; LEMD1 |
| TC0X00006938.hg.1 | 3.93 | 3.7 | -1.18 | 0.0056 | CFAP47 |
| TC0700010692.hg.1 | 11.41 | 11.17 | -1.18 | 0.006 | NT5C3A |
| TC1100011658.hg.1 | 6.77 | 6.53 | -1.18 | 0.0074 | MAP6 |
| TC1900011762.hg.1 | 6.68 | 6.45 | -1.18 | 0.0079 | APOC2 |
| TC0200016412.hg.1 | 6.56 | 6.32 | -1.18 | 0.0086 | MFSD2B |
| TC0900012161.hg.1 | 8.68 | 8.45 | -1.18 | 0.0087 | DNAJC25-GNG10 |
| TC2000007495.hg.1 | 5.79 | 5.56 | -1.18 | 0.009 | WFDC10A |
| TC0X00009262.hg.1 | 7.56 | 7.34 | -1.17 | 0.0002 | SUPT20HL2 |
| TC0100006920.hg.1 | 4.5 | 4.28 | -1.17 | 0.0003 | PRAMEF9 |
| TC1400008209.hg.1 | 6.78 | 6.56 | -1.17 | 0.0003 | SLC25A47 |
| TC0700012812.hg.1 | 17.05 | 16.83 | -1.17 | 0.0006 | MKRN1 |
| TC1700012370.hg.1 | 6.48 | 6.25 | -1.17 | 0.0007 | TBC1D28 |
| TC1200009558.hg.1 | 12.81 | 12.57 | -1.17 | 0.0008 | NINJ2 |
| TC1700012205.hg.1 | 10.63 | 10.4 | -1.17 | 0.0009 | PRPSAP2 |
| TC1700012247.hg.1 | 9.06 | 8.83 | -1.17 | 0.0009 | TCAP |
| TC0300008933.hg.1 | 4.85 | 4.62 | -1.17 | 0.0013 | CLDN18 |
| TC0600010777.hg.1 | 6.06 | 5.84 | -1.17 | 0.0013 | TFAP2A |
| TC1700006667.hg.1 | 7.8 | 7.58 | -1.17 | 0.002 | USP6 |
| TC1200007787.hg.1 | 4.44 | 4.21 | -1.17 | 0.0022 | OR6C3 |
| TC0300011550.hg.1 | 6.39 | 6.17 | -1.17 | 0.0022 | PDZRN3 |
| TC0500007258.hg.1 | 4.04 | 3.81 | -1.17 | 0.0023 | GHR |
| TC0100016086.hg.1 | 8.23 | 8.01 | -1.17 | 0.0027 | AIM2 |
| TC1100006721.hg.1 | 4.02 | 3.8 | -1.17 | 0.0027 | CNGA4 |
| TC0400012810.hg.1 | 10.15 | 9.93 | -1.17 | 0.0031 | MOB1B |
| TC0700012871.hg.1 | 4.81 | 4.58 | -1.17 | 0.0035 | OR9A2 |
| TC0500007666.hg.1 | 7.61 | 7.38 | -1.17 | 0.0037 | CENPH |
| TC1000012472.hg.1 | 9.16 | 8.93 | -1.17 | 0.0038 | FAM25A |
| TC1600011557.hg.1 | 11.56 | 11.34 | -1.17 | 0.0041 | TERF2 |
| TSUnmapped00000347.hg.1 | 6.06 | 5.83 | -1.17 | 0.0044 | ZNF780A |
| TC0600011999.hg.1 | 5.83 | 5.6 | -1.17 | 0.0046 | RHAG |
| TC2000007859.hg.1 | 14.87 | 14.64 | -1.17 | 0.0048 | RBM38 |
| TC0100011698.hg.1 | 7.61 | 7.38 | -1.17 | 0.0054 | DISP1 |
| TC0600008729.hg.1 | 4.33 | 4.1 | -1.17 | 0.006 | C6orf163 |
| TC1100007005.hg.1 | 5.02 | 4.79 | -1.17 | 0.0066 | LDHC |
| TC0700012813.hg.1 | 6.04 | 5.81 | -1.17 | 0.0071 | DENND2A |
| TC0300012768.hg.1 | 3.91 | 3.68 | -1.17 | 0.0074 | CP |
| TC1500007699.hg.1 | 6.6 | 6.37 | -1.17 | 0.0078 | KIF23 |
| TC1700007057.hg.1 | 7.94 | 7.72 | -1.17 | 0.0079 | NT5M |
| TC0200013878.hg.1 | 6.4 | 6.18 | -1.17 | 0.0081 | BUB1 |
| TC0700012870.hg.1 | 7.65 | 7.42 | -1.17 | 0.0081 | KEL |
| TC2100007166.hg.1 | 5.74 | 5.52 | -1.17 | 0.0081 | SH3BGR |
| TC2100006873.hg.1 | 4.36 | 4.13 | -1.17 | 0.0084 | KRTAP13-1 |
| TC0600011354.hg.1 | 6.76 | 6.54 | -1.17 | 0.0088 | TRIM10 |
| TC0100010416.hg.1 | 7.49 | 7.26 | -1.17 | 0.0089 | PBX1 |
| TC1000008396.hg.1 | 10.61 | 10.39 | -1.17 | 0.0096 | IFIT2 |
| TC1600008940.hg.1 | 8.4 | 8.17 | -1.17 | 0.0099 | AXIN1 |
| TC1800007969.hg.1 | 5.62 | 5.4 | -1.17 | 0.01 | L3MBTL4 |
| TC1700007014.hg.1 | 17.22 | 17 | -1.16 | 0.0001 | UBB |
| TC0200007402.hg.1 | 3.54 | 3.32 | -1.16 | 0.0004 | ABCG8 |
| TC1900007888.hg.1 | 5.72 | 5.51 | -1.16 | 0.0007 | APLP1 |
| TC1900010568.hg.1 | 5.73 | 5.52 | -1.16 | 0.0008 | ZNF569 |
| TC1300009376.hg.1 | 4.71 | 4.5 | -1.16 | 0.0008 | SLITRK1 |
| TC1700012015.hg.1 | 7.2 | 6.98 | -1.16 | 0.0009 | AATK |
| TC1400009698.hg.1 | 4.71 | 4.49 | -1.16 | 0.001 | LTBP2 |
| TC0100016625.hg.1 | 15.66 | 15.45 | -1.16 | 0.0011 | GLUL |
| TC0400011408.hg.1 | 4.51 | 4.29 | -1.16 | 0.0011 | ADH1B |
| TC0X00007522.hg.1 | 6.61 | 6.4 | -1.16 | 0.0012 | FAM155B |
| TC1000009012.hg.1 | 4.42 | 4.21 | -1.16 | 0.0015 | ENO4 |
| TC1900011229.hg.1 | 4.28 | 4.06 | -1.16 | 0.0015 | KLK6 |
| TC1100009434.hg.1 | 4.81 | 4.59 | -1.16 | 0.0024 | DDX25 |
| TC1100010885.hg.1 | 5.13 | 4.91 | -1.16 | 0.0025 | TNKS1BP1 |
| TC1100011983.hg.1 | 4.62 | 4.4 | -1.16 | 0.0029 | VSTM5 |
| TC0100010672.hg.1 | 11.16 | 10.95 | -1.16 | 0.0033 | RABGAP1L |
| TC0200008300.hg.1 | 12.55 | 12.34 | -1.16 | 0.0035 | RMND5A |
| TC2200006630.hg.1 | 12 | 11.79 | -1.16 | 0.0037 | TANGO2 |
| TC1700010241.hg.1 | 6.43 | 6.21 | -1.16 | 0.0038 | NUFIP2; rerdy |
| TC1500006999.hg.1 | 5.85 | 5.63 | -1.16 | 0.0039 | NUSAP1 |
| TC0300011386.hg.1 | 6.79 | 6.58 | -1.16 | 0.0039 | PRICKLE2 |
| TSUnmapped00000127.hg.1 | 5.34 | 5.12 | -1.16 | 0.0046 | KIF15 |
| TC0700013526.hg.1 | 9.63 | 9.41 | -1.16 | 0.0046 | FAM126A |
| TC0X00011082.hg.1 | 5.68 | 5.46 | -1.16 | 0.0051 | HSFX2; HSFX1 |
| TC0X00010990.hg.1 | 6.79 | 6.58 | -1.16 | 0.0053 | SPANXC; SPANXD |
| TC0200010340.hg.1 | 3.68 | 3.46 | -1.16 | 0.0054 | CCDC150 |
| TC0900012113.hg.1 | 4.49 | 4.28 | -1.16 | 0.006 | IFNA8 |
| TC0600008078.hg.1 | 5.07 | 4.86 | -1.16 | 0.0064 | SLC22A7 |
| TC0800009621.hg.1 | 4.77 | 4.55 | -1.16 | 0.0068 | CTSB |
| TC1100009406.hg.1 | 4.44 | 4.23 | -1.16 | 0.0068 | HEPN1 |
| TC0X00007163.hg.1 | 4.74 | 4.52 | -1.16 | 0.0074 | SPACA5 |
| TC1900009350.hg.1 | 4.83 | 4.61 | -1.16 | 0.0078 | PLIN4 |
| TC0900010913.hg.1 | 4.57 | 4.36 | -1.16 | 0.0078 | ZNF367 |
| TC0100012358.hg.1 | 6.06 | 5.84 | -1.16 | 0.0081 | OR2T2 |
| TC0900007321.hg.1 | 5.06 | 4.85 | -1.16 | 0.0082 | SPATA31A5; SPATA31A7 |
| TC1400008349.hg.1 | 6.83 | 6.62 | -1.16 | 0.0083 | TECPR2 |
| TC1000008397.hg.1 | 11.44 | 11.22 | -1.16 | 0.0085 | IFIT3 |
| TC0X00009677.hg.1 | 5.01 | 4.8 | -1.16 | 0.0085 | AKAP4 |
| TC0500008883.hg.1 | 8.53 | 8.32 | -1.16 | 0.0087 | PCDHB12 |
| TC0500009751.hg.1 | 10.01 | 9.8 | -1.16 | 0.0087 | BTNL9 |
| TC0900011256.hg.1 | 6.49 | 6.28 | -1.16 | 0.0092 | RNF183 |
| TC0800012467.hg.1 | 4.52 | 4.31 | -1.16 | 0.0098 | OC90 |
| TC2000007337.hg.1 | 7.07 | 6.86 | -1.16 | 0.0098 | PPP1R16B |
| TC0100018506.hg.1 | 13.87 | 13.68 | -1.15 | 5.47E-05 | YY1AP1 |
| TC0100016971.hg.1 | 17.84 | 17.63 | -1.15 | 7.77E-05 | ADIPOR1 |
| TC1200009724.hg.1 | 6.49 | 6.29 | -1.15 | 0.0002 | SCNN1A |
| TC1100011131.hg.1 | 15.68 | 15.48 | -1.15 | 0.0003 | C11orf95 |
| TC1100009918.hg.1 | 12.39 | 12.2 | -1.15 | 0.0003 | HBD |
| TC1600008910.hg.1 | 10.94 | 10.73 | -1.15 | 0.0006 | GAS8 |
| TC0800007321.hg.1 | 11.44 | 11.24 | -1.15 | 0.0006 | ASH2L |
| TC1200007833.hg.1 | 6.06 | 5.86 | -1.15 | 0.0006 | SLC39A5 |
| TSUnmapped00000203.hg.1 | 9.85 | 9.64 | -1.15 | 0.0012 | KAT6B |
| TC0300008355.hg.1 | 4.66 | 4.45 | -1.15 | 0.0013 | BOC |
| TC0900012146.hg.1 | 6.3 | 6.1 | -1.15 | 0.0013 | SPATA31D1 |
| TC1900011763.hg.1 | 5.73 | 5.53 | -1.15 | 0.0016 | APOC4-APOC2; APOC2 |
| TC1100006888.hg.1 | 4.17 | 3.96 | -1.15 | 0.0016 | TEAD1 |
| TC0800007027.hg.1 | 15.67 | 15.47 | -1.15 | 0.0017 | SLC25A37 |
| TC1400010593.hg.1 | 9.08 | 8.88 | -1.15 | 0.002 | FAM177A1 |
| TC0600011125.hg.1 | 6.36 | 6.16 | -1.15 | 0.0022 | HIST1H2AB |
| TC1900012017.hg.1 | 7.29 | 7.09 | -1.15 | 0.0024 | CLDND2 |
| TC1600010244.hg.1 | 4.35 | 4.15 | -1.15 | 0.0026 | ZNF423 |
| TC0X00006558.hg.1 | 7.21 | 7.01 | -1.15 | 0.0027 | VCX; VCX3A |
| TC1600009656.hg.1 | 6.14 | 5.94 | -1.15 | 0.003 | PDILT |
| TC0100007838.hg.1 | 6.3 | 6.1 | -1.15 | 0.0031 | DNALI1 |
| TC1600011515.hg.1 | 8.36 | 8.16 | -1.15 | 0.0034 | AC009133.12; PAGR1 |
| TC0700013599.hg.1 | 6.37 | 6.17 | -1.15 | 0.0034 | ACHE |
| TC0X00007602.hg.1 | 4.87 | 4.67 | -1.15 | 0.0035 | PABPC1L2B |
| TC1300008086.hg.1 | 5.2 | 5 | -1.15 | 0.0037 | ATP11A |
| TC0200016065.hg.1 | 6.86 | 6.65 | -1.15 | 0.0039 | ECEL1 |
| TC0300012155.hg.1 | 5.31 | 5.11 | -1.15 | 0.0039 | HGD |
| TC0400008103.hg.1 | 6.17 | 5.97 | -1.15 | 0.0039 | RP11-10L7.1; HERC6 |
| TC2200008008.hg.1 | 4.77 | 4.56 | -1.15 | 0.0045 | ARVCF |
| TC1600006645.hg.1 | 7.21 | 7.01 | -1.15 | 0.0046 | FLYWCH1 |
| TC1000009221.hg.1 | 11.58 | 11.38 | -1.15 | 0.0047 | ZRANB1 |
| TC1700010397.hg.1 | 3.9 | 3.69 | -1.15 | 0.0047 | C17orf102 |
| TC1800007155.hg.1 | 10.83 | 10.62 | -1.15 | 0.005 | PIK3C3 |
| TC1100013134.hg.1 | 6.21 | 6 | -1.15 | 0.0051 | HBE1 |
| TC1100006896.hg.1 | 5.4 | 5.2 | -1.15 | 0.0053 | RASSF10 |
| TC0Y00007203.hg.1 | 5.22 | 5.02 | -1.15 | 0.0071 | PRY; PRY2 |
| TC1400006739.hg.1 | 5.91 | 5.71 | -1.15 | 0.0073 | NFATC4 |
| TC1100011485.hg.1 | 6.52 | 6.32 | -1.15 | 0.0073 | SHANK2 |
| TC0600007568.hg.1 | 6.56 | 6.35 | -1.15 | 0.0077 | HCG27 |
| TC0X00008820.hg.1 | 4.77 | 4.57 | -1.15 | 0.0079 | OPN1LW |
| TC0700013254.hg.1 | 5.69 | 5.48 | -1.15 | 0.0081 | PTPRN2 |
| TC0100007748.hg.1 | 5.09 | 4.89 | -1.15 | 0.0084 | GJA4 |
| TC1900008544.hg.1 | 7.44 | 7.23 | -1.15 | 0.0084 | RCN3 |
| TC0100013374.hg.1 | 5.12 | 4.92 | -1.15 | 0.0086 | TRIM63 |
| TC1200009399.hg.1 | 4.78 | 4.58 | -1.15 | 0.009 | PIWIL1 |
| TC0200006476.hg.1 | 6.22 | 6.03 | -1.15 | 0.0096 | SNTG2 |
| TC1000008363.hg.1 | 15.36 | 15.17 | -1.14 | 0.0002 | PTEN |
| TC1100009678.hg.1 | 6.17 | 5.98 | -1.14 | 0.0002 | HRAS |
| TC1500008370.hg.1 | 4.21 | 4.02 | -1.14 | 0.0003 | ST8SIA2 |
| TC1600006530.hg.1 | 3.52 | 3.33 | -1.14 | 0.0009 | TPSD1 |
| TC0600008183.hg.1 | 10.2 | 10.01 | -1.14 | 0.0009 | CD2AP |
| TC0100009029.hg.1 | 9.69 | 9.51 | -1.14 | 0.001 | BRDT |
| TC0600008050.hg.1 | 12.89 | 12.7 | -1.14 | 0.001 | UBR2 |
| TC0200009903.hg.1 | 3.73 | 3.54 | -1.14 | 0.001 | G6PC2 |
| TC0600014329.hg.1 | 7.29 | 7.1 | -1.14 | 0.0013 | OSTM1 |
| TC0100015725.hg.1 | 10.63 | 10.44 | -1.14 | 0.0015 | ANP32E |
| TC1900009668.hg.1 | 7.81 | 7.62 | -1.14 | 0.0017 | SPC24 |
| TC1100007453.hg.1 | 5.81 | 5.63 | -1.14 | 0.0019 | MDK |
| TC0200014276.hg.1 | 4.07 | 3.88 | -1.14 | 0.0023 | CFC1 |
| TC0100007574.hg.1 | 17.03 | 16.84 | -1.14 | 0.0026 | EPB41 |
| TC1100006995.hg.1 | 4.14 | 3.95 | -1.14 | 0.0027 | SAA1 |
| TC0900012275.hg.1 | 7.63 | 7.45 | -1.14 | 0.0034 | GOLGA1 |
| TC1100011622.hg.1 | 3.62 | 3.43 | -1.14 | 0.0041 | CHRDL2 |
| TC1700006860.hg.1 | 4.47 | 4.28 | -1.14 | 0.0042 | GLP2R |
| TC0200013600.hg.1 | 5.87 | 5.68 | -1.14 | 0.0043 | KIAA1211L |
| TC1200007523.hg.1 | 3.5 | 3.32 | -1.14 | 0.0054 | OR8S1 |
| TC0400009431.hg.1 | 4.81 | 4.62 | -1.14 | 0.0057 | TENM3 |
| TC0100018344.hg.1 | 9.8 | 9.62 | -1.14 | 0.0058 | LYPLAL1 |
| TC0800008352.hg.1 | 11.89 | 11.7 | -1.14 | 0.0061 | VPS13B |
| TC1800007298.hg.1 | 3.37 | 3.17 | -1.14 | 0.0067 | LIPG |
| TC0300009139.hg.1 | 5.42 | 5.24 | -1.14 | 0.0073 | TM4SF4 |
| TC0200008447.hg.1 | 5.47 | 5.28 | -1.14 | 0.0078 | TEKT4 |
| TC1400006741.hg.1 | 4.5 | 4.3 | -1.14 | 0.0079 | NYNRIN |
| TC2000009360.hg.1 | 8.89 | 8.71 | -1.14 | 0.008 | PREX1 |
| TC1700011101.hg.1 | 4.85 | 4.66 | -1.14 | 0.0089 | CHAD |
| TC0X00010050.hg.1 | 4.37 | 4.18 | -1.14 | 0.0091 | PABPC1L2A |
| TC1700011652.hg.1 | 8.72 | 8.52 | -1.14 | 0.0091 | FAM104A |
| TC0400011565.hg.1 | 5.39 | 5.2 | -1.14 | 0.0093 | COL25A1 |
| TC1100008557.hg.1 | 8.15 | 7.96 | -1.14 | 0.0096 | USP35 |
| TC1000011023.hg.1 | 6.37 | 6.18 | -1.14 | 0.0097 | MSS51 |
| TC1700011793.hg.1 | 4.48 | 4.3 | -1.14 | 0.0098 | QRICH2 |
| TC0200011268.hg.1 | 8.34 | 8.15 | -1.14 | 0.0098 | RAMP1 |
| TC0100015971.hg.1 | 15.12 | 14.94 | -1.13 | 5.39E-05 | GON4L |
| TC0200009424.hg.1 | 7.4 | 7.22 | -1.13 | 0.0007 | ACMSD |
| TC0X00009632.hg.1 | 9.05 | 8.87 | -1.13 | 0.0013 | PCSK1N |
| TC0100016279.hg.1 | 6.79 | 6.61 | -1.13 | 0.0015 | RP11-9L18.3; FAM78B |
| TC0100007573.hg.1 | 4.59 | 4.42 | -1.13 | 0.0023 | OPRD1 |
| TC1600008735.hg.1 | 7.8 | 7.63 | -1.13 | 0.0027 | FOXF1 |
| TC1900012018.hg.1 | 7.72 | 7.54 | -1.13 | 0.0029 | ZNF577 |
| TC1900011253.hg.1 | 10.11 | 9.93 | -1.13 | 0.0033 | C19orf84 |
| TC0X00008737.hg.1 | 7.87 | 7.69 | -1.13 | 0.0035 | CNGA2 |
| TC1000007905.hg.1 | 8.03 | 7.85 | -1.13 | 0.0036 | C10orf35 |
| TC0700010376.hg.1 | 10.23 | 10.05 | -1.13 | 0.0037 | SNX13 |
| TC2200008799.hg.1 | 12.64 | 12.46 | -1.13 | 0.0039 | ST13 |
| TC1400006713.hg.1 | 4.43 | 4.25 | -1.13 | 0.0039 | LRRC16B |
| TC0100010006.hg.1 | 5.24 | 5.07 | -1.13 | 0.0043 | KPRP |
| TC1500007757.hg.1 | 5.58 | 5.41 | -1.13 | 0.0053 | NR2E3 |
| TC2100008356.hg.1 | 5.3 | 5.12 | -1.13 | 0.0054 | DNMT3L |
| TC1700007536.hg.1 | 4.86 | 4.68 | -1.13 | 0.0055 | SPACA3 |
| TC1900008906.hg.1 | 6.22 | 6.05 | -1.13 | 0.0056 | TMEM190 |
| TC1900010595.hg.1 | 8.2 | 8.02 | -1.13 | 0.0056 | PPP1R14A |
| TC1200012598.hg.1 | 3.46 | 3.29 | -1.13 | 0.0059 | MGST1 |
| TC1200006643.hg.1 | 9 | 8.83 | -1.13 | 0.006 | PTMS |
| TC0900010052.hg.1 | 4.24 | 4.06 | -1.13 | 0.0061 | IGFBPL1 |
| TC0500012463.hg.1 | 6.17 | 6 | -1.13 | 0.0061 | CAMK2A |
| TC0X00008824.hg.1 | 12.31 | 12.13 | -1.13 | 0.0067 | TKTL1 |
| TC2200008022.hg.1 | 6.67 | 6.5 | -1.13 | 0.0067 | CCDC188 |
| TC1200007992.hg.1 | 4.52 | 4.34 | -1.13 | 0.0068 | SRGAP1 |
| TC1600009163.hg.1 | 4.47 | 4.3 | -1.13 | 0.0068 | CCDC64B |
| TC2000007269.hg.1 | 4.12 | 3.95 | -1.13 | 0.0069 | TLDC2 |
| TSUnmapped00000573.hg.1 | 5.05 | 4.87 | -1.13 | 0.0071 | PRAMEF25 |
| TSUnmapped00000657.hg.1 | 4.16 | 3.98 | -1.13 | 0.0073 | PRAMEF25 |
| TSUnmapped00000795.hg.1 | 4.16 | 3.98 | -1.13 | 0.0073 | PRAMEF25 |
| TC1600011494.hg.1 | 12.36 | 12.19 | -1.13 | 0.008 | ARL6IP1 |
| TC1400007298.hg.1 | 9.92 | 9.74 | -1.13 | 0.008 | ACTR10 |
| TC0700008567.hg.1 | 7.34 | 7.17 | -1.13 | 0.009 | TRIP6; MIR6875 |
| TC0600011402.hg.1 | 6.24 | 6.06 | -1.13 | 0.0092 | CDSN |
| TC1000007794.hg.1 | 5.16 | 4.98 | -1.13 | 0.0092 | JMJD1C-AS1 |
| TC1500010752.hg.1 | 4.27 | 4.09 | -1.13 | 0.0093 | TLN2 |
| TC0100018222.hg.1 | 3.78 | 3.61 | -1.13 | 0.0095 | LOC100129924; C1orf50; RP5-994D16.11 |
| TC2000009243.hg.1 | 4.78 | 4.6 | -1.13 | 0.0098 | TNNC2 |
| TC1700006762.hg.1 | 4.57 | 4.4 | -1.13 | 0.0098 | SHBG |
| TC0900010607.hg.1 | 10.35 | 10.17 | -1.13 | 0.0098 | AGTPBP1 |
| TC1500008271.hg.1 | 4.49 | 4.31 | -1.13 | 0.0099 | WDR93 |
| TC2000009882.hg.1 | 16.66 | 16.5 | -1.12 | 0.0003 | PTPRA; VPS16 |
| TC0100009067.hg.1 | 10.51 | 10.34 | -1.12 | 0.0008 | FNBP1L |
| TC2000007141.hg.1 | 5.16 | 4.99 | -1.12 | 0.0016 | BPIFB3 |
| TSUnmapped00000187.hg.1 | 4.11 | 3.95 | -1.12 | 0.0016 | ZNF197 |
| TC0800010490.hg.1 | 9.3 | 9.13 | -1.12 | 0.0023 | TMEM68 |
| TC0200011097.hg.1 | 6.88 | 6.72 | -1.12 | 0.0025 | ALPPL2 |
| TC1900011648.hg.1 | 5 | 4.85 | -1.12 | 0.0039 | CAPS |
| TC0100011453.hg.1 | 6.92 | 6.76 | -1.12 | 0.0039 | G0S2 |
| TC0400006936.hg.1 | 3.18 | 3.01 | -1.12 | 0.0042 | C1QTNF7 |
| TC0400012820.hg.1 | 3.57 | 3.41 | -1.12 | 0.0044 | FGF5 |
| TC1900007851.hg.1 | 8.52 | 8.36 | -1.12 | 0.0046 | FFAR1 |
| TC1100012422.hg.1 | 7.71 | 7.55 | -1.12 | 0.0046 | APOA4 |
| TC1900011482.hg.1 | 5 | 4.84 | -1.12 | 0.0048 | SBK3 |
| TC0900006451.hg.1 | 4.08 | 3.92 | -1.12 | 0.0052 | DMRT1 |
| TSUnmapped00000151.hg.1 | 5.72 | 5.56 | -1.12 | 0.0053 | ADAMTS13 |
| TC0X00011290.hg.1 | 3.99 | 3.83 | -1.12 | 0.0056 | GAGE1 |
| TC1600008867.hg.1 | 5.22 | 5.05 | -1.12 | 0.0064 | LOC400558; RP11-46C24.3 |
| TC0100018514.hg.1 | 4.04 | 3.88 | -1.12 | 0.0065 | VSIG8 |
| TC0900008951.hg.1 | 4.23 | 4.06 | -1.12 | 0.0066 | HMCN2 |
| TC1000012581.hg.1 | 9.74 | 9.58 | -1.12 | 0.0067 | ARHGAP19-SLIT1 |
| TC1900010715.hg.1 | 6.15 | 5.98 | -1.12 | 0.007 | NUMBL |
| TC1600010022.hg.1 | 6.43 | 6.27 | -1.12 | 0.0075 | COX6A2 |
| TC1900009637.hg.1 | 7.55 | 7.39 | -1.12 | 0.0077 | CDKN2D |
| TC0400007938.hg.1 | 14.92 | 14.75 | -1.12 | 0.0077 | BMP2K |
| TC0100010184.hg.1 | 10.39 | 10.22 | -1.12 | 0.008 | TTC24 |
| TC2000010032.hg.1 | 6.09 | 5.93 | -1.12 | 0.0085 | CHRNA4 |
| TC0700011574.hg.1 | 7.53 | 7.36 | -1.12 | 0.0086 | SPDYE16 |
| TC0100006819.hg.1 | 8.04 | 7.88 | -1.12 | 0.0087 | APITD1-CORT; CORT; APITD1 |
| TC1900010963.hg.1 | 4.91 | 4.74 | -1.12 | 0.0088 | NANOS2 |
| TC0700013345.hg.1 | 4.59 | 4.43 | -1.12 | 0.0089 | INMT |
| TC1300006919.hg.1 | 3.36 | 3.2 | -1.12 | 0.0093 | FREM2 |
| TC0100018563.hg.1 | 6.23 | 6.08 | -1.11 | 0.001 | LEFTY1 |
| TC1000009908.hg.1 | 15.18 | 15.03 | -1.11 | 0.0016 | RSU1 |
| TC1000012416.hg.1 | 4.72 | 4.57 | -1.11 | 0.0026 | FRG2B |
| TC0200015773.hg.1 | 5.64 | 5.49 | -1.11 | 0.0034 | PTPRN |
| TC1500008454.hg.1 | 2.78 | 2.63 | -1.11 | 0.0039 | SPATA8 |
| TC0900009351.hg.1 | 5.71 | 5.56 | -1.11 | 0.0041 | C9orf66 |
| TC2100008251.hg.1 | 4.08 | 3.93 | -1.11 | 0.0041 | TFF1 |
| TC0100010188.hg.1 | 3.37 | 3.22 | -1.11 | 0.0047 | BCAN |
| TC0500009566.hg.1 | 5.91 | 5.76 | -1.11 | 0.0051 | CPLX2 |
| TC1000011696.hg.1 | 4.73 | 4.57 | -1.11 | 0.0052 | CYP17A1 |
| TC0500012659.hg.1 | 6.49 | 6.33 | -1.11 | 0.0068 | FABP6 |
| TC1200012648.hg.1 | 13.16 | 13.01 | -1.11 | 0.0071 | MYL6 |
| TC1000007428.hg.1 | 4.95 | 4.8 | -1.11 | 0.0075 | ZNF32-AS3 |
| TC1400007232.hg.1 | 8.07 | 7.92 | -1.11 | 0.0079 | FBXO34 |
| TC0900008741.hg.1 | 5.65 | 5.49 | -1.11 | 0.0086 | WDR38 |
| TC0800008631.hg.1 | 5.54 | 5.39 | -1.11 | 0.0087 | AARD |
| TC0200008801.hg.1 | 4.67 | 4.52 | -1.11 | 0.0096 | SULT1C4 |
| TC1700008914.hg.1 | 8.08 | 7.93 | -1.11 | 0.0098 | SMIM5 |
| TC1300006715.hg.1 | 11.6 | 11.46 | -1.1 | 0.0011 | PAN3; RNU6-82P |
| TC1000009997.hg.1 | 4.55 | 4.41 | -1.1 | 0.0055 | CASC10 |
| TC1800007518.hg.1 | 8.98 | 8.84 | -1.1 | 0.0062 | ZCCHC2 |
| TC1700008984.hg.1 | 14.55 | 14.42 | -1.1 | 0.0076 | SEC14L1; SCARNA16; SNHG20; MIR6516 |
| TC0Y00006569.hg.1 | 3.42 | 3.28 | -1.1 | 0.008 | TSPY3; TSPY4; TSPY8; TSPY1 |
| TC1000011190.hg.1 | 6.69 | 6.55 | -1.1 | 0.0081 | SFTPD |
| TC0X00010837.hg.1 | 17.34 | 17.2 | -1.1 | 0.0085 | MBNL3 |
| TC1000008206.hg.1 | 4.3 | 4.17 | -1.1 | 0.0087 | SFTPA1 |
| TC0100015953.hg.1 | 4.88 | 4.75 | -1.1 | 0.009 | PKLR |
| TC0200016770.hg.1 | 4.14 | 4 | -1.1 | 0.0095 | MIR6809; TNS1 |
| TC1900006896.hg.1 | 15.39 | 15.27 | -1.09 | 0.0001 | HNRNPM |
| TC1600011452.hg.1 | 4.24 | 4.13 | -1.09 | 0.003 | MC1R |
| TC1900011101.hg.1 | 4.12 | 3.99 | -1.09 | 0.0049 | PLEKHA4 |
| TC0300012598.hg.1 | 3.48 | 3.36 | -1.09 | 0.0062 | RBP1 |
| TC0700008389.hg.1 | 4.29 | 4.17 | -1.09 | 0.0087 | ASB4 |
| TC0300012585.hg.1 | 3.21 | 3.09 | -1.09 | 0.0089 | FOXL2 |
| TC1100007266.hg.1 | 8.74 | 8.61 | -1.09 | 0.009 | PDHX |
| TC1500010757.hg.1 | 11.9 | 11.78 | -1.09 | 0.0093 | RAB11A |
| TC0600013193.hg.1 | 9.55 | 9.42 | -1.09 | 0.0095 | STX7 |
| TC1900011997.hg.1 | 4.78 | 4.65 | -1.09 | 0.0096 | CGB7 |
| TC1100009280.hg.1 | 6.86 | 6.73 | -1.09 | 0.0097 | OAF |
| TC1900009352.hg.1 | 4.8 | 4.69 | -1.08 | 0.0044 | SEMA6B |
| TC0100008025.hg.1 | 16.66 | 16.55 | -1.08 | 0.0052 | YBX1 |
| TC0200010952.hg.1 | 3.25 | 3.14 | -1.08 | 0.0094 | NYAP2 |
| TC1000011657.hg.1 | 6.77 | 6.66 | -1.08 | 0.0096 | FGF8 |
| TC1900011486.hg.1 | 4.43 | 4.33 | -1.07 | 0.0086 | ZNF784 |
| TC1900006700.hg.1 | 9.18 | 9.1 | -1.06 | 0.0081 | HDGFRP2 |
| TC1700006719.hg.1 | 17.36 | 17.44 | 1.06 | 0.0031 | RNASEK; C17orf49; RNASEK-C17orf49 |
| TC0X00008829.hg.1 | 18.28 | 18.37 | 1.06 | 0.005 | RPL10; SNORA70 |
| TC0200016476.hg.1 | 12.27 | 12.35 | 1.06 | 0.0071 | WBP1 |
| TC0600012927.hg.1 | 9.61 | 9.7 | 1.06 | 0.009 | HDAC2 |
| TC1100008067.hg.1 | 14.68 | 14.78 | 1.07 | 0.0018 | RAB1B |
| TC0600007605.hg.1 | 13.92 | 14.02 | 1.07 | 0.0037 | LY6G5B; CSNK2B |
| TC1700011038.hg.1 | 12.23 | 12.33 | 1.07 | 0.0042 | ZNF652 |
| TC1000012577.hg.1 | 11.65 | 11.74 | 1.07 | 0.0051 | LIPA |
| TC1700012352.hg.1 | 15.02 | 15.12 | 1.07 | 0.006 | TRAPPC1 |
| TC0500011754.hg.1 | 11.77 | 11.87 | 1.07 | 0.007 | TMED7-TICAM2; TICAM2; TMED7 |
| TC0X00008521.hg.1 | 10.5 | 10.6 | 1.07 | 0.0087 | SLC9A6 |
| TC0600011583.hg.1 | 7.83 | 7.93 | 1.07 | 0.009 | C6orf1 |
| TC0600006855.hg.1 | 10.89 | 10.99 | 1.07 | 0.0096 | RREB1 |
| TC1600011342.hg.1 | 12.32 | 12.42 | 1.08 | 0.002 | NAA60 |
| TC1100007247.hg.1 | 14.18 | 14.28 | 1.08 | 0.0021 | CAPRIN1 |
| TC1700007725.hg.1 | 10.51 | 10.62 | 1.08 | 0.003 | LASP1 |
| TC0100014806.hg.1 | 13.07 | 13.19 | 1.08 | 0.0037 | 15-ספט |
| TC1100011241.hg.1 | 12.12 | 12.23 | 1.08 | 0.0037 | MAP3K11 |
| TC1900009316.hg.1 | 16.31 | 16.43 | 1.08 | 0.005 | EEF2; SNORD37 |
| TC1200007767.hg.1 | 12.02 | 12.13 | 1.08 | 0.0058 | NCKAP1L |
| TC1700009541.hg.1 | 10.79 | 10.9 | 1.08 | 0.0066 | CAMTA2 |
| TC1100010705.hg.1 | 10.88 | 11 | 1.08 | 0.007 | AMBRA1 |
| TC1000010438.hg.1 | 13.01 | 13.12 | 1.08 | 0.0071 | HNRNPF |
| TC1900008384.hg.1 | 13.2 | 13.3 | 1.08 | 0.0078 | CALM3 |
| TC1200008162.hg.1 | 12.08 | 12.19 | 1.08 | 0.0084 | CNOT2 |
| TC2100007797.hg.1 | 14.55 | 14.66 | 1.08 | 0.0085 | ATP5J |
| TC0800010103.hg.1 | 10.45 | 10.56 | 1.08 | 0.0087 | LSM12 |
| TC2000007572.hg.1 | 12.14 | 12.26 | 1.08 | 0.0087 | NCOA3 |
| TC1100008462.hg.1 | 15.79 | 15.9 | 1.08 | 0.0089 | RPS3; SNORD15A |
| TC0100007451.hg.1 | 15.79 | 15.9 | 1.08 | 0.0091 | CD52 |
| TC1100009864.hg.1 | 12.68 | 12.8 | 1.08 | 0.0092 | RHOG |
| TC1500010884.hg.1 | 15.11 | 15.23 | 1.08 | 0.0094 | RPL4; SNORD16; SNORD18A; SNORD18B; SNORD18C |
| TC1700010076.hg.1 | 11.84 | 11.95 | 1.08 | 0.0095 | USP22 |
| TC0500009620.hg.1 | 10.04 | 10.16 | 1.08 | 0.0098 | RGS14 |
| TC1600007440.hg.1 | 15.26 | 15.39 | 1.09 | 0.0003 | ALDOA |
| TC1200009843.hg.1 | 12.82 | 12.95 | 1.09 | 0.0006 | M6PR |
| TC1200010900.hg.1 | 13.36 | 13.48 | 1.09 | 0.0011 | SMARCC2 |
| TC1700012052.hg.1 | 17.02 | 17.15 | 1.09 | 0.0011 | ACTG1 |
| TC1500007856.hg.1 | 10.4 | 10.53 | 1.09 | 0.0012 | CSK |
| TC0100006729.hg.1 | 11.24 | 11.36 | 1.09 | 0.0014 | PARK7 |
| TC0200016416.hg.1 | 9.95 | 10.07 | 1.09 | 0.0015 | SLC35F6 |
| TC1900011713.hg.1 | 12.3 | 12.43 | 1.09 | 0.0015 | PSENEN |
| TC0600014267.hg.1 | 13.09 | 13.22 | 1.09 | 0.0017 | ATF6B |
| TC0200016595.hg.1 | 15.6 | 15.72 | 1.09 | 0.0022 | RPL37A |
| TC1100012735.hg.1 | 13.43 | 13.55 | 1.09 | 0.0022 | SRPR |
| TSUnmapped00000380.hg.1 | 12.09 | 12.22 | 1.09 | 0.0023 | FBL |
| TC1200012716.hg.1 | 12.73 | 12.86 | 1.09 | 0.0026 | COX6A1 |
| TC0700010167.hg.1 | 12.69 | 12.81 | 1.09 | 0.0026 | ACTB |
| TC1700008917.hg.1 | 10.3 | 10.43 | 1.09 | 0.0029 | SAP30BP |
| TC1200012789.hg.1 | 12.14 | 12.27 | 1.09 | 0.0029 | PRKAG1 |
| TC0600014258.hg.1 | 15.49 | 15.61 | 1.09 | 0.0031 | HLA-B |
| TC0600008146.hg.1 | 12.65 | 12.77 | 1.09 | 0.0032 | RUNX2 |
| TC0100018502.hg.1 | 10.58 | 10.71 | 1.09 | 0.0034 | GBA |
| TC0300006694.hg.1 | 11.75 | 11.88 | 1.09 | 0.0038 | NR2C2 |
| TC1300009994.hg.1 | 11.62 | 11.75 | 1.09 | 0.0039 | ERCC5 |
| TC2200007309.hg.1 | 11.96 | 12.08 | 1.09 | 0.0042 | SH3BP1; PDXP |
| TC1100007938.hg.1 | 10.69 | 10.81 | 1.09 | 0.0047 | FERMT3 |
| TC0200015506.hg.1 | 10.59 | 10.72 | 1.09 | 0.0054 | INO80D |
| TC0X00009205.hg.1 | 13.12 | 13.24 | 1.09 | 0.0058 | SH3KBP1 |
| TC0800012374.hg.1 | 9.1 | 9.23 | 1.09 | 0.0059 | GLI4 |
| TC0500013430.hg.1 | 17.1 | 17.22 | 1.09 | 0.0062 | GNB2L1; SNORD95; SNORD96A |
| TC0X00010643.hg.1 | 13.09 | 13.21 | 1.09 | 0.0062 | 06-ספט |
| TC0900011519.hg.1 | 12.36 | 12.48 | 1.09 | 0.0064 | PPP6C |
| TC2200008781.hg.1 | 11.09 | 11.21 | 1.09 | 0.0065 | MKL1 |
| TC1700010447.hg.1 | 17.07 | 17.18 | 1.09 | 0.0065 | CCL5 |
| TC1300008125.hg.1 | 11.11 | 11.23 | 1.09 | 0.0069 | LAMP1 |
| TC0900006970.hg.1 | 10.82 | 10.94 | 1.09 | 0.007 | NFX1 |
| TC1700006654.hg.1 | 9.7 | 9.83 | 1.09 | 0.0073 | RNF167 |
| TC2000008860.hg.1 | 9.12 | 9.24 | 1.09 | 0.0073 | COMMD7 |
| TC1100010712.hg.1 | 12.49 | 12.61 | 1.09 | 0.0076 | ARHGAP1 |
| TC0100010592.hg.1 | 12.87 | 12.99 | 1.09 | 0.0084 | PRRC2C |
| TC1900011767.hg.1 | 9.19 | 9.31 | 1.09 | 0.0084 | BLOC1S3 |
| TC1500007072.hg.1 | 12.36 | 12.49 | 1.09 | 0.0086 | PDIA3 |
| TSUnmapped00000819.hg.1 | 10.09 | 10.22 | 1.09 | 0.0088 | PCYT1A |
| TC1600010476.hg.1 | 10.69 | 10.82 | 1.09 | 0.0088 | CSNK2A2 |
| TC0500007792.hg.1 | 12.56 | 12.68 | 1.09 | 0.0093 | HEXB |
| TC1600007234.hg.1 | 9.52 | 9.65 | 1.09 | 0.0093 | DCTN5 |
| TC1000011683.hg.1 | 10.7 | 10.82 | 1.09 | 0.0094 | ACTR1A |
| TC1900008840.hg.1 | 13.93 | 14.06 | 1.09 | 0.0095 | RPS9 |
| TC1700008216.hg.1 | 9.5 | 9.62 | 1.09 | 0.0096 | KAT7 |
| TC0100013134.hg.1 | 15.36 | 15.49 | 1.1 | 5.29E-05 | CAPZB |
| TC0100015982.hg.1 | 15.32 | 15.46 | 1.1 | 0.0003 | SSR2 |
| TC1900011795.hg.1 | 13.56 | 13.7 | 1.1 | 0.0008 | NDUFA3 |
| TC0100013924.hg.1 | 11.75 | 11.89 | 1.1 | 0.0011 | ELOVL1; MIR6734 |
| TC0100013123.hg.1 | 11.99 | 12.13 | 1.1 | 0.0012 | UBR4 |
| TC0X00011310.hg.1 | 13.12 | 13.27 | 1.1 | 0.0012 | RPL36A-HNRNPH2 |
| TC0600007855.hg.1 | 9.62 | 9.76 | 1.1 | 0.0012 | FGD2 |
| TC2100008562.hg.1 | 13.55 | 13.69 | 1.1 | 0.0015 | RUNX1 |
| TC1100010222.hg.1 | 7.63 | 7.76 | 1.1 | 0.0016 | RPL36A |
| TC1900010659.hg.1 | 15.27 | 15.41 | 1.1 | 0.0016 | RPS16 |
| TC1900011789.hg.1 | 10.25 | 10.38 | 1.1 | 0.0016 | EMC10 |
| TC0100013472.hg.1 | 11.82 | 11.96 | 1.1 | 0.0018 | DNAJC8 |
| TC1700007383.hg.1 | 15.87 | 16.01 | 1.1 | 0.0019 | RPL23A; SNORD4B; SNORD42B; SNORD42A |
| TC2200008434.hg.1 | 12.72 | 12.86 | 1.1 | 0.0022 | SF3A1 |
| TC0300013788.hg.1 | 9.68 | 9.82 | 1.1 | 0.0022 | ARPC4-TTLL3 |
| TC0X00011308.hg.1 | 13.92 | 14.06 | 1.1 | 0.0023 | RPL36A |
| TC0200012961.hg.1 | 11.95 | 12.09 | 1.1 | 0.0023 | AAK1 |
| TC0100009365.hg.1 | 11.22 | 11.37 | 1.1 | 0.0023 | AHCYL1 |
| TC0400012757.hg.1 | 12.53 | 12.67 | 1.1 | 0.0031 | RNF4 |
| TC1100013186.hg.1 | 11.74 | 11.88 | 1.1 | 0.0032 | SYVN1 |
| TC0100018185.hg.1 | 11.09 | 11.23 | 1.1 | 0.0035 | MINOS1 |
| TC1900006509.hg.1 | 6.49 | 6.63 | 1.1 | 0.0035 | HMHA1 |
| TC1200009445.hg.1 | 10.21 | 10.35 | 1.1 | 0.0038 | SFSWAP |
| TC0700007098.hg.1 | 10.79 | 10.93 | 1.1 | 0.0039 | GARS |
| TC1600007354.hg.1 | 13.21 | 13.34 | 1.1 | 0.0039 | EIF3C; EIF3CL |
| TC1000010961.hg.1 | 15.76 | 15.9 | 1.1 | 0.004 | PSAP |
| TC0X00007369.hg.1 | 6.14 | 6.27 | 1.1 | 0.004 | APEX2 |
| TC1200012808.hg.1 | 10.04 | 10.18 | 1.1 | 0.0041 | R3HDM2 |
| TC1600006995.hg.1 | 11.26 | 11.4 | 1.1 | 0.0041 | BFAR |
| TC0200011889.hg.1 | 13.44 | 13.58 | 1.1 | 0.0043 | LAPTM4A |
| TC0100007326.hg.1 | 16.09 | 16.23 | 1.1 | 0.0045 | RPL11 |
| TC1900010743.hg.1 | 13.95 | 14.09 | 1.1 | 0.0048 | TGFB1 |
| TC0300007359.hg.1 | 13.03 | 13.16 | 1.1 | 0.0048 | NDUFAF3 |
| TC0X00007529.hg.1 | 10.54 | 10.68 | 1.1 | 0.0051 | IGBP1 |
| TC1600010633.hg.1 | 13.68 | 13.82 | 1.1 | 0.0053 | ATP6V0D1 |
| TC0500007591.hg.1 | 9.26 | 9.4 | 1.1 | 0.0053 | CWC27 |
| TC0300010310.hg.1 | 16.3 | 16.44 | 1.1 | 0.0054 | RPL32; SNORA7A |
| TC2000009909.hg.1 | 10.92 | 11.07 | 1.1 | 0.0058 | RALY |
| TC1700009036.hg.1 | 10.57 | 10.71 | 1.1 | 0.0063 | TMC8 |
| TC0400009467.hg.1 | 9.66 | 9.8 | 1.1 | 0.0066 | TRAPPC11 |
| TC0600007675.hg.1 | 11.94 | 12.07 | 1.1 | 0.0067 | BRD2 |
| TC1400010778.hg.1 | 8.32 | 8.46 | 1.1 | 0.0067 | TRIP11 |
| TC0100016139.hg.1 | 13.65 | 13.78 | 1.1 | 0.0067 | CD48 |
| TC2000010002.hg.1 | 10.21 | 10.35 | 1.1 | 0.0067 | NCOA6 |
| TC1100008123.hg.1 | 11.58 | 11.72 | 1.1 | 0.0068 | ADRBK1 |
| TC1100013028.hg.1 | 11 | 11.14 | 1.1 | 0.0069 | OTUB1 |
| TC2200007477.hg.1 | 10.95 | 11.09 | 1.1 | 0.007 | ACO2 |
| TC2100008233.hg.1 | 9.78 | 9.92 | 1.1 | 0.0071 | ZBTB21 |
| TC1700010910.hg.1 | 10.52 | 10.65 | 1.1 | 0.0078 | KANSL1 |
| TC0100015819.hg.1 | 14.84 | 14.97 | 1.1 | 0.0078 | S100A10 |
| TC0700008868.hg.1 | 12.57 | 12.71 | 1.1 | 0.008 | TES |
| TC0500009706.hg.1 | 11.7 | 11.84 | 1.1 | 0.008 | SQSTM1 |
| TC1700007638.hg.1 | 10.55 | 10.68 | 1.1 | 0.0085 | GGNBP2 |
| TC1300006633.hg.1 | 10.44 | 10.57 | 1.1 | 0.0087 | PABPC3 |
| TC2000010007.hg.1 | 11.55 | 11.68 | 1.1 | 0.0088 | RBM12; CPNE1 |
| TC0800012212.hg.1 | 10.06 | 10.2 | 1.1 | 0.009 | SLC39A4 |
| TC2000009023.hg.1 | 15.46 | 15.59 | 1.1 | 0.0093 | SAMHD1 |
| TC1900006924.hg.1 | 8.62 | 8.76 | 1.1 | 0.0093 | ZNF317 |
| TC2000006464.hg.1 | 9.72 | 9.86 | 1.1 | 0.0096 | FAM110A |
| TC1400007691.hg.1 | 7.86 | 8 | 1.1 | 0.0098 | DLST |
| TC1900010696.hg.1 | 10.96 | 11.09 | 1.1 | 0.0099 | AKT2 |
| TC0100012778.hg.1 | 14.35 | 14.5 | 1.11 | 7.85E-05 | ENO1 |
| TC1700007890.hg.1 | 11.38 | 11.53 | 1.11 | 0.0001 | ATP6V0A1; MIR5010 |
| TC0500013205.hg.1 | 12.1 | 12.26 | 1.11 | 0.0003 | CAST |
| TC0900007576.hg.1 | 15.49 | 15.64 | 1.11 | 0.0006 | ANXA1 |
| TC0X00008687.hg.1 | 9.39 | 9.54 | 1.11 | 0.0007 | CXorf40A |
| TC1000011050.hg.1 | 11.93 | 12.07 | 1.11 | 0.0007 | AP3M1 |
| TC1000011669.hg.1 | 13.74 | 13.9 | 1.11 | 0.0007 | LDB1 |
| TC0X00007222.hg.1 | 16.35 | 16.5 | 1.11 | 0.0008 | PLP2 |
| TC2000010027.hg.1 | 13.05 | 13.2 | 1.11 | 0.0008 | TMEM189-UBE2V1 |
| TC0300012346.hg.1 | 14.61 | 14.76 | 1.11 | 0.0011 | RPN1 |
| TC0X00011168.hg.1 | 12.12 | 12.27 | 1.11 | 0.0013 | BCAP31 |
| TC0200008230.hg.1 | 14.08 | 14.23 | 1.11 | 0.0013 | TMSB10 |
| TC0100015902.hg.1 | 11.17 | 11.31 | 1.11 | 0.0015 | C1orf43 |
| TC1000008236.hg.1 | 11.79 | 11.94 | 1.11 | 0.0017 | TSPAN14 |
| TC1900008019.hg.1 | 14.44 | 14.59 | 1.11 | 0.0017 | EIF3K |
| TC0200013578.hg.1 | 12.36 | 12.52 | 1.11 | 0.0018 | TMEM131 |
| TC0700007285.hg.1 | 12.02 | 12.17 | 1.11 | 0.0019 | CDK13 |
| TC2100007597.hg.1 | 8.13 | 8.29 | 1.11 | 0.002 | HSPA13 |
| TC0600007544.hg.1 | 13.01 | 13.16 | 1.11 | 0.0021 | TUBB |
| TC0300007495.hg.1 | 11.17 | 11.32 | 1.11 | 0.0022 | SPCS1 |
| TC1900008056.hg.1 | 7.47 | 7.62 | 1.11 | 0.0024 | MED29 |
| TC1600008228.hg.1 | 9.63 | 9.78 | 1.11 | 0.0024 | CYB5B |
| TC1100008505.hg.1 | 10.93 | 11.08 | 1.11 | 0.0026 | EMSY |
| TC1100006491.hg.1 | 16.47 | 16.62 | 1.11 | 0.0026 | RPLP2; SNORA52 |
| TC1000008744.hg.1 | 9.68 | 9.83 | 1.11 | 0.0027 | TRIM8 |
| TC0400010965.hg.1 | 10.94 | 11.09 | 1.11 | 0.0028 | GRSF1 |
| TC0700012596.hg.1 | 9.18 | 9.33 | 1.11 | 0.0029 | TMEM209 |
| TC0X00008437.hg.1 | 9.67 | 9.82 | 1.11 | 0.0029 | STK26 |
| TC0500008008.hg.1 | 12.7 | 12.86 | 1.11 | 0.0031 | COX7C; MIR3607 |
| TSUnmapped00000333.hg.1 | 10.54 | 10.7 | 1.11 | 0.0031 | SURF4 |
| TC1200010839.hg.1 | 11.32 | 11.47 | 1.11 | 0.0031 | ITGA5 |
| TC0100015891.hg.1 | 11.17 | 11.32 | 1.11 | 0.0032 | CRTC2 |
| TC1000008276.hg.1 | 13.15 | 13.3 | 1.11 | 0.0032 | CCSER2 |
| TC1200010618.hg.1 | 13.37 | 13.52 | 1.11 | 0.0033 | TUBA1A |
| TC0700012695.hg.1 | 9.43 | 9.58 | 1.11 | 0.0035 | C7orf49 |
| TC1500009736.hg.1 | 14.77 | 14.92 | 1.11 | 0.0035 | PPIB |
| TC1800008578.hg.1 | 8.97 | 9.12 | 1.11 | 0.0036 | EPG5 |
| TC1600007255.hg.1 | 11.9 | 12.05 | 1.11 | 0.0037 | RBBP6 |
| TC0700010604.hg.1 | 12.01 | 12.17 | 1.11 | 0.0037 | CPVL |
| TC0100016159.hg.1 | 10.75 | 10.9 | 1.11 | 0.0038 | DEDD |
| TC1700008734.hg.1 | 10.2 | 10.35 | 1.11 | 0.0038 | AMZ2 |
| TC1200011400.hg.1 | 10.03 | 10.18 | 1.11 | 0.0039 | CCDC59 |
| TC0600007792.hg.1 | 8.94 | 9.09 | 1.11 | 0.0039 | PPARD |
| TC0100007886.hg.1 | 10.33 | 10.48 | 1.11 | 0.0041 | NDUFS5 |
| TC1700008049.hg.1 | 10.54 | 10.69 | 1.11 | 0.0041 | FMNL1 |
| TC0200009938.hg.1 | 10.92 | 11.07 | 1.11 | 0.0041 | GORASP2 |
| TC0200016252.hg.1 | 9.54 | 9.69 | 1.11 | 0.0042 | HDAC4 |
| TC0700007986.hg.1 | 8.18 | 8.33 | 1.11 | 0.0044 | WBSCR22 |
| TC0200006674.hg.1 | 8.8 | 8.96 | 1.11 | 0.0045 | KLF11 |
| TC1900008505.hg.1 | 9.72 | 9.87 | 1.11 | 0.0045 | BAX |
| TC1100009983.hg.1 | 8.3 | 8.45 | 1.11 | 0.0046 | MRPL17 |
| TC0100015866.hg.1 | 12.05 | 12.19 | 1.11 | 0.0049 | S100A4 |
| TC1700011523.hg.1 | 9.05 | 9.2 | 1.11 | 0.0049 | PSMD12 |
| TC1600010011.hg.1 | 11.66 | 11.81 | 1.11 | 0.0053 | PYCARD |
| TC1600010475.hg.1 | 10.05 | 10.2 | 1.11 | 0.0054 | CFAP20 |
| TC1100011539.hg.1 | 10.33 | 10.49 | 1.11 | 0.0054 | NUMA1 |
| TSUnmapped00000061.hg.1 | 12.77 | 12.92 | 1.11 | 0.0054 | FBL |
| TC1900012025.hg.1 | 8.11 | 8.26 | 1.11 | 0.0056 | ZNF28 |
| TSUnmapped00000183.hg.1 | 9.27 | 9.42 | 1.11 | 0.0058 | VPS11 |
| TC1400009714.hg.1 | 12.03 | 12.18 | 1.11 | 0.006 | TMED10 |
| TC1900009432.hg.1 | 9.4 | 9.55 | 1.11 | 0.006 | DENND1C |
| TC0100007716.hg.1 | 8.57 | 8.73 | 1.11 | 0.0061 | ZNF362 |
| TC0900009268.hg.1 | 8.43 | 8.57 | 1.11 | 0.0062 | NDOR1 |
| TC1900008121.hg.1 | 10.84 | 10.98 | 1.11 | 0.0063 | SNRPA |
| TC0800006989.hg.1 | 8.42 | 8.56 | 1.11 | 0.0063 | CCAR2 |
| TC1100013176.hg.1 | 10.33 | 10.48 | 1.11 | 0.0065 | HNRNPUL2 |
| TC0800007435.hg.1 | 9.29 | 9.44 | 1.11 | 0.0065 | IKBKB |
| TC1700010849.hg.1 | 10.11 | 10.26 | 1.11 | 0.0066 | EFTUD2 |
| TC0700012701.hg.1 | 9.3 | 9.46 | 1.11 | 0.0066 | CNOT4 |
| TC0100018300.hg.1 | 6.98 | 7.13 | 1.11 | 0.0067 | ADAM15 |
| TC0900008959.hg.1 | 8.2 | 8.34 | 1.11 | 0.0069 | FUBP3 |
| TC1900011654.hg.1 | 11.53 | 11.69 | 1.11 | 0.0071 | PNPLA6 |
| TC1400010716.hg.1 | 8.1 | 8.25 | 1.11 | 0.0074 | HOMEZ |
| TC0100007765.hg.1 | 9.3 | 9.45 | 1.11 | 0.0076 | ZMYM4 |
| TC0600009142.hg.1 | 9.01 | 9.16 | 1.11 | 0.008 | RPF2 |
| TC1900008297.hg.1 | 9.29 | 9.44 | 1.11 | 0.0081 | CLPTM1 |
| TC0X00008831.hg.1 | 9.9 | 10.05 | 1.11 | 0.0081 | ATP6AP1 |
| TC0100014226.hg.1 | 12.02 | 12.17 | 1.11 | 0.0082 | C1orf123 |
| TC0X00007012.hg.1 | 12.07 | 12.23 | 1.11 | 0.0084 | ATP6AP2 |
| TC1000012589.hg.1 | 8.87 | 9.02 | 1.11 | 0.0084 | MRPL43 |
| TC2000006446.hg.1 | 8.67 | 8.82 | 1.11 | 0.0086 | RBCK1 |
| TC1100011092.hg.1 | 12.36 | 12.52 | 1.11 | 0.0086 | GANAB |
| TC1700011743.hg.1 | 9.12 | 9.28 | 1.11 | 0.0086 | GGA3 |
| TC0400011978.hg.1 | 7.87 | 8.01 | 1.11 | 0.0087 | TBC1D9 |
| TC0200016727.hg.1 | 9.05 | 9.2 | 1.11 | 0.0087 | IWS1 |
| TC0800008546.hg.1 | 9.96 | 10.11 | 1.11 | 0.0088 | ENY2 |
| TC0900009308.hg.1 | 8.21 | 8.36 | 1.11 | 0.0089 | EHMT1 |
| TC0200016648.hg.1 | 10.27 | 10.43 | 1.11 | 0.009 | CDC42EP3 |
| TC0500013232.hg.1 | 11.25 | 11.41 | 1.11 | 0.0091 | SMAD5 |
| TC1600007973.hg.1 | 8.52 | 8.67 | 1.11 | 0.0093 | NUP93 |
| TC1900011932.hg.1 | 7.46 | 7.61 | 1.11 | 0.0093 | ALKBH6 |
| TC1900009487.hg.1 | 7.12 | 7.27 | 1.11 | 0.0094 | XAB2 |
| TC0X00011194.hg.1 | 13.26 | 13.41 | 1.11 | 0.0099 | FLNA |
| TC0700007811.hg.1 | 9.65 | 9.79 | 1.11 | 0.0099 | INTS4P2 |
| TC0900010462.hg.1 | 8.06 | 8.21 | 1.11 | 0.01 | RFK |
| TC1200006629.hg.1 | 16.88 | 17.05 | 1.12 | 2.84E-05 | GAPDH |
| TC1900007839.hg.1 | 15.54 | 15.7 | 1.12 | 6.83E-05 | FXYD5 |
| TC1000006891.hg.1 | 15.21 | 15.37 | 1.12 | 7.91E-05 | VIM |
| TC0600013361.hg.1 | 11.83 | 11.99 | 1.12 | 0.0002 | CITED2 |
| TC1100011089.hg.1 | 11.97 | 12.14 | 1.12 | 0.0002 | MTA2 |
| TC0200013516.hg.1 | 13.85 | 14.01 | 1.12 | 0.0004 | TMEM127 |
| TC0900012175.hg.1 | 11.72 | 11.89 | 1.12 | 0.0006 | CDK9 |
| TC1000010310.hg.1 | 8.67 | 8.84 | 1.12 | 0.0007 | CUL2 |
| TC0900011918.hg.1 | 14.44 | 14.6 | 1.12 | 0.0008 | FCN1 |
| TC1500009636.hg.1 | 15.2 | 15.37 | 1.12 | 0.0008 | ANXA2 |
| TSUnmapped00000477.hg.1 | 10.58 | 10.74 | 1.12 | 0.0008 | SURF4 |
| TC2000007105.hg.1 | 12.86 | 13.02 | 1.12 | 0.0009 | TM9SF4 |
| TC2000006899.hg.1 | 12.17 | 12.34 | 1.12 | 0.0009 | XRN2 |
| TC1700010599.hg.1 | 9.95 | 10.11 | 1.12 | 0.001 | MED24; MIR6884 |
| TC0300010240.hg.1 | 8.29 | 8.45 | 1.12 | 0.0012 | SEC13 |
| TC1600010794.hg.1 | 7.43 | 7.59 | 1.12 | 0.0012 | AP1G1 |
| TC0600008066.hg.1 | 9.66 | 9.82 | 1.12 | 0.0012 | KLHDC3 |
| TC0600007495.hg.1 | 14.72 | 14.88 | 1.12 | 0.0013 | HLA-A |
| TC0800008295.hg.1 | 9.46 | 9.62 | 1.12 | 0.0013 | PTDSS1 |
| TC0700007997.hg.1 | 7.59 | 7.76 | 1.12 | 0.0014 | LIMK1 |
| TC1000011988.hg.1 | 12.31 | 12.48 | 1.12 | 0.0014 | EIF3A |
| TC2100008392.hg.1 | 14 | 14.15 | 1.12 | 0.0015 | ITGB2 |
| TC1700007777.hg.1 | 8.8 | 8.96 | 1.12 | 0.0015 | THRA |
| TC1100008012.hg.1 | 11.72 | 11.88 | 1.12 | 0.0015 | DPF2 |
| TC0400007593.hg.1 | 12.51 | 12.67 | 1.12 | 0.0015 | SRP72 |
| TC2200009161.hg.1 | 7.8 | 7.97 | 1.12 | 0.0016 | LMF2 |
| TC1100013004.hg.1 | 7.91 | 8.07 | 1.12 | 0.0016 | CTNND1 |
| TC1100013190.hg.1 | 15.65 | 15.82 | 1.12 | 0.0017 | CFL1 |
| TC1700010831.hg.1 | 11.55 | 11.72 | 1.12 | 0.0017 | GPATCH8 |
| TC0X00007148.hg.1 | 12.57 | 12.73 | 1.12 | 0.0017 | ARAF |
| TC1100013003.hg.1 | 10.17 | 10.34 | 1.12 | 0.0018 | TMX2; C11orf31 |
| TC0100015951.hg.1 | 10.25 | 10.41 | 1.12 | 0.0018 | SCAMP3 |
| TC1100008342.hg.1 | 9.75 | 9.91 | 1.12 | 0.0019 | INPPL1 |
| TC0100013696.hg.1 | 10.29 | 10.45 | 1.12 | 0.0019 | PSMB2 |
| TC1400009784.hg.1 | 9.73 | 9.89 | 1.12 | 0.002 | VIPAS39 |
| TC0500013401.hg.1 | 14.73 | 14.9 | 1.12 | 0.0021 | LCP2 |
| TC1700011917.hg.1 | 8.31 | 8.47 | 1.12 | 0.0022 | USP36 |
| TC0500013277.hg.1 | 9 | 9.17 | 1.12 | 0.0022 | MAML1 |
| TC1700008028.hg.1 | 9.57 | 9.73 | 1.12 | 0.0024 | NMT1 |
| TC0600014129.hg.1 | 8.28 | 8.44 | 1.12 | 0.0024 | RRP36 |
| TC0200007058.hg.1 | 11.36 | 11.52 | 1.12 | 0.0024 | ATRAID |
| TC1900009757.hg.1 | 11.08 | 11.24 | 1.12 | 0.0025 | FARSA |
| TC1600011368.hg.1 | 11.25 | 11.42 | 1.12 | 0.0025 | LAT |
| TC0X00011173.hg.1 | 13.27 | 13.43 | 1.12 | 0.0025 | IDH3G |
| TC1600011386.hg.1 | 8 | 8.16 | 1.12 | 0.0026 | ORAI3 |
| TC1100006726.hg.1 | 7.04 | 7.21 | 1.12 | 0.0026 | SMPD1 |
| TC1100008174.hg.1 | 10.43 | 10.59 | 1.12 | 0.0027 | ALDH3B1 |
| TC1100011794.hg.1 | 10.97 | 11.13 | 1.12 | 0.0028 | PRCP |
| TC0200015741.hg.1 | 7.33 | 7.49 | 1.12 | 0.0028 | RNF25 |
| TC1900010851.hg.1 | 8.02 | 8.19 | 1.12 | 0.0028 | ETHE1 |
| TC2200008687.hg.1 | 9.24 | 9.41 | 1.12 | 0.0028 | TMEM184B |
| TC2000009401.hg.1 | 8.21 | 8.37 | 1.12 | 0.0029 | SPATA2 |
| TC0800012240.hg.1 | 16.35 | 16.52 | 1.12 | 0.003 | RPL8; MIR6850 |
| TC0300012208.hg.1 | 11.96 | 12.13 | 1.12 | 0.003 | HACD2 |
| TC1700007381.hg.1 | 11.93 | 12.1 | 1.12 | 0.003 | SUPT6H |
| TC0100012490.hg.1 | 6.52 | 6.69 | 1.12 | 0.0031 | C1orf233 |
| TC2200008744.hg.1 | 17.07 | 17.23 | 1.12 | 0.0031 | RPL3; SNORD83B; SNORD43 |
| TC2200008966.hg.1 | 10.66 | 10.83 | 1.12 | 0.0031 | KIAA0930 |
| TC0600009712.hg.1 | 12.74 | 12.9 | 1.12 | 0.0032 | UTRN |
| TC1000011596.hg.1 | 7.58 | 7.74 | 1.12 | 0.0033 | DNMBP |
| TC0700006562.hg.1 | 8.89 | 9.06 | 1.12 | 0.0033 | LFNG; MIR4648 |
| TC0100013455.hg.1 | 8.34 | 8.51 | 1.12 | 0.0034 | RPA2 |
| TC0900011819.hg.1 | 9.93 | 10.1 | 1.12 | 0.0035 | TSC1 |
| TC0100014014.hg.1 | 10.97 | 11.12 | 1.12 | 0.0035 | PRDX1 |
| TC1900007396.hg.1 | 12.01 | 12.17 | 1.12 | 0.0036 | KXD1 |
| TC1700008563.hg.1 | 11.31 | 11.47 | 1.12 | 0.0037 | DCAF7 |
| TC2000010026.hg.1 | 10.62 | 10.78 | 1.12 | 0.0038 | TMEM189 |
| TC0800012451.hg.1 | 14.36 | 14.52 | 1.12 | 0.0039 | EIF3E |
| TC1700012076.hg.1 | 11.82 | 11.99 | 1.12 | 0.004 | ARHGDIA |
| TC0700008762.hg.1 | 12.81 | 12.97 | 1.12 | 0.004 | CBLL1 |
| TC0200016772.hg.1 | 8.17 | 8.33 | 1.12 | 0.0042 | NHEJ1 |
| TC0200006647.hg.1 | 8.1 | 8.26 | 1.12 | 0.0042 | CPSF3 |
| TC11_KI270721v1_random00006432.hg.1 | 7.45 | 7.61 | 1.12 | 0.0044 | MRPL23; LOC102724828; AC004556.1 |
| TC0X00007195.hg.1 | 11.77 | 11.94 | 1.12 | 0.0047 | RBM3 |
| TC1500007554.hg.1 | 9.53 | 9.69 | 1.12 | 0.005 | ZNF609 |
| TC2200007894.hg.1 | 14.44 | 14.61 | 1.12 | 0.005 | CECR1 |
| TC1000011323.hg.1 | 11 | 11.16 | 1.12 | 0.0051 | GLUD1 |
| TC2100008000.hg.1 | 8.85 | 9.01 | 1.12 | 0.0051 | GART |
| TSUnmapped00000445.hg.1 | 12.21 | 12.37 | 1.12 | 0.0051 | FBL |
| TC0700008360.hg.1 | 10.68 | 10.85 | 1.12 | 0.0052 | CASD1 |
| TC0500013423.hg.1 | 13.21 | 13.37 | 1.12 | 0.0054 | HNRNPH1 |
| TC0600014360.hg.1 | 12.24 | 12.4 | 1.12 | 0.0059 | IPCEF1 |
| TC1700011451.hg.1 | 8.52 | 8.68 | 1.12 | 0.0059 | SMURF2 |
| TC1900008560.hg.1 | 7.73 | 7.9 | 1.12 | 0.006 | PTOV1; MIR4749 |
| TC1600009856.hg.1 | 12.16 | 12.33 | 1.12 | 0.006 | EIF3C; EIF3CL |
| TC0600011809.hg.1 | 13.81 | 13.98 | 1.12 | 0.006 | CCND3 |
| TC0500012611.hg.1 | 11.61 | 11.77 | 1.12 | 0.0062 | CLINT1 |
| TC0600013409.hg.1 | 9.46 | 9.62 | 1.12 | 0.0062 | HIVEP2 |
| TC1700012304.hg.1 | 9.15 | 9.31 | 1.12 | 0.0062 | COG1 |
| TC0400008271.hg.1 | 10.75 | 10.91 | 1.12 | 0.007 | NFKB1 |
| TC1700007658.hg.1 | 10.64 | 10.8 | 1.12 | 0.007 | AATF |
| TC1900011941.hg.1 | 10.47 | 10.64 | 1.12 | 0.0071 | HNRNPL |
| TC1200009461.hg.1 | 10.73 | 10.9 | 1.12 | 0.0071 | EP400; SNORA49 |
| TC1900010074.hg.1 | 10.44 | 10.61 | 1.12 | 0.0073 | GMIP |
| TC1400008963.hg.1 | 8.94 | 9.1 | 1.12 | 0.0073 | MBIP |
| TC0500012942.hg.1 | 8.6 | 8.76 | 1.12 | 0.0073 | RNF44 |
| TC1700009498.hg.1 | 12.56 | 12.72 | 1.12 | 0.0075 | ATP2A3 |
| TC0600007664.hg.1 | 10.18 | 10.34 | 1.12 | 0.0075 | HLA-DQA2 |
| TC1700009650.hg.1 | 6.38 | 6.55 | 1.12 | 0.0076 | SAT2 |
| TC0X00009256.hg.1 | 8.58 | 8.75 | 1.12 | 0.0078 | KLHL15 |
| TC0100013760.hg.1 | 9.64 | 9.8 | 1.12 | 0.0078 | SF3A3 |
| TC0700008306.hg.1 | 9.33 | 9.49 | 1.12 | 0.0079 | FZD1 |
| TC1600009956.hg.1 | 9.81 | 9.97 | 1.12 | 0.0079 | BOLA2B; BOLA2 |
| TC0X00009427.hg.1 | 8.78 | 8.94 | 1.12 | 0.008 | BCOR |
| TC1000012575.hg.1 | 10.4 | 10.56 | 1.12 | 0.0083 | ATAD1 |
| TC1200011961.hg.1 | 7.6 | 7.76 | 1.12 | 0.0086 | TMEM116 |
| TC0100007038.hg.1 | 9.68 | 9.84 | 1.12 | 0.0086 | NECAP2 |
| TC1200009748.hg.1 | 9.47 | 9.63 | 1.12 | 0.0087 | ZNF384 |
| TC0X00007224.hg.1 | 6.44 | 6.61 | 1.12 | 0.0087 | CCDC22 |
| TC1100008612.hg.1 | 16.25 | 16.41 | 1.12 | 0.0087 | RPS28 |
| TC0400012934.hg.1 | 10.54 | 10.71 | 1.12 | 0.0088 | SDAD1 |
| TC0700011518.hg.1 | 8.4 | 8.57 | 1.12 | 0.0089 | GTF2IRD2; GTF2IRD2B |
| TC1600008220.hg.1 | 9.82 | 9.97 | 1.12 | 0.0089 | UTP4 |
| TC1700012349.hg.1 | 7.47 | 7.63 | 1.12 | 0.009 | NAA38 |
| TC1700012324.hg.1 | 8.36 | 8.52 | 1.12 | 0.009 | ABR |
| TC1900007382.hg.1 | 7.26 | 7.42 | 1.12 | 0.009 | PGPEP1 |
| TC0600011531.hg.1 | 8.55 | 8.71 | 1.12 | 0.0091 | VPS52 |
| TC0900007494.hg.1 | 7.83 | 7.99 | 1.12 | 0.0092 | FXN |
| TC0100007283.hg.1 | 8.04 | 8.21 | 1.12 | 0.0092 | ZBTB40 |
| TC2000007386.hg.1 | 7.76 | 7.92 | 1.12 | 0.0093 | PLCG1; RPL23AP81 |
| TC1200012574.hg.1 | 8.57 | 8.73 | 1.12 | 0.0094 | TULP3 |
| TC2000009800.hg.1 | 10.55 | 10.71 | 1.12 | 0.0095 | YTHDF1 |
| TC0100016135.hg.1 | 13.95 | 14.11 | 1.12 | 0.0097 | SLAMF6 |
| TC0600013001.hg.1 | 8.18 | 8.35 | 1.12 | 0.0098 | CEP85L |
| TC0600014088.hg.1 | 7.73 | 7.9 | 1.12 | 0.0098 | ZSCAN26 |
| TC0X00008874.hg.1 | 12.29 | 12.47 | 1.13 | 5.39E-05 | F8A2 |
| TC1600007448.hg.1 | 16.68 | 16.86 | 1.13 | 6.38E-05 | CORO1A |
| TC0500012210.hg.1 | 12.68 | 12.86 | 1.13 | 6.70E-05 | TMEM173 |
| TC1100007273.hg.1 | 12.38 | 12.56 | 1.13 | 7.91E-05 | CD44 |
| TC0100014065.hg.1 | 11.41 | 11.58 | 1.13 | 0.0001 | EFCAB14 |
| TC1000006703.hg.1 | 12.27 | 12.44 | 1.13 | 0.0002 | ATP5C1 |
| TC2200008611.hg.1 | 11.74 | 11.92 | 1.13 | 0.0003 | EIF3D |
| TC1900011889.hg.1 | 10.36 | 10.53 | 1.13 | 0.0003 | MAN2B1 |
| TC1200008568.hg.1 | 10.09 | 10.26 | 1.13 | 0.0005 | TMPO |
| TC1500010047.hg.1 | 12.31 | 12.49 | 1.13 | 0.0005 | SCAMP2 |
| TC0300010213.hg.1 | 10.72 | 10.9 | 1.13 | 0.0006 | RPUSD3 |
| TC1500007700.hg.1 | 14.77 | 14.94 | 1.13 | 0.0006 | RPLP1 |
| TC1200010281.hg.1 | 10.62 | 10.79 | 1.13 | 0.0007 | FAM60A |
| TC0400011744.hg.1 | 12.95 | 13.12 | 1.13 | 0.0007 | ANXA5 |
| TC2000008845.hg.1 | 8.55 | 8.73 | 1.13 | 0.0008 | PLAGL2 |
| TC0900011597.hg.1 | 10.87 | 11.05 | 1.13 | 0.0008 | SH2D3C |
| TC0100015728.hg.1 | 11.94 | 12.12 | 1.13 | 0.0009 | APH1A |
| TC0800011561.hg.1 | 14.3 | 14.48 | 1.13 | 0.0009 | EIF3H |
| TC1900007313.hg.1 | 10.01 | 10.19 | 1.13 | 0.0009 | USE1 |
| TC1100013185.hg.1 | 11.21 | 11.39 | 1.13 | 0.0011 | FAU |
| TC1900008862.hg.1 | 10.27 | 10.45 | 1.13 | 0.0011 | LAIR2 |
| TC0100016917.hg.1 | 10.6 | 10.78 | 1.13 | 0.0011 | CSRP1 |
| TC1200010616.hg.1 | 16.5 | 16.69 | 1.13 | 0.0011 | TUBA1B |
| TC0500007138.hg.1 | 16.72 | 16.9 | 1.13 | 0.0012 | IL7R |
| TC2200009028.hg.1 | 9.36 | 9.53 | 1.13 | 0.0012 | CERK |
| TC2000008055.hg.1 | 10.93 | 11.11 | 1.13 | 0.0013 | ARFGAP1; MIR4326 |
| TC0200016679.hg.1 | 8.1 | 8.28 | 1.13 | 0.0015 | SERTAD2 |
| TC0600014154.hg.1 | 8.32 | 8.49 | 1.13 | 0.0015 | SLC35A1 |
| TC1200012202.hg.1 | 10.42 | 10.6 | 1.13 | 0.0016 | ANAPC5 |
| TC1300008855.hg.1 | 8.84 | 9.01 | 1.13 | 0.0017 | ESD |
| TC2000009204.hg.1 | 8.23 | 8.41 | 1.13 | 0.0017 | TOMM34 |
| TC1600007113.hg.1 | 10.52 | 10.7 | 1.13 | 0.0017 | C16orf62 |
| TC0100011203.hg.1 | 10.62 | 10.8 | 1.13 | 0.0018 | RNPEP |
| TC2200006678.hg.1 | 11.27 | 11.44 | 1.13 | 0.0019 | SNAP29 |
| TC0500012979.hg.1 | 10.86 | 11.03 | 1.13 | 0.0019 | DDX41 |
| TC0100013305.hg.1 | 8.75 | 8.93 | 1.13 | 0.0019 | FUCA1 |
| TSUnmapped00000801.hg.1 | 10.91 | 11.09 | 1.13 | 0.0019 | VPS11 |
| TC0700007505.hg.1 | 12.73 | 12.91 | 1.13 | 0.0021 | IKZF1 |
| TC1600008128.hg.1 | 9.45 | 9.63 | 1.13 | 0.0021 | CMTM3 |
| TC0100018273.hg.1 | 12.49 | 12.66 | 1.13 | 0.0021 | ATP1A1 |
| TC0200008632.hg.1 | 14.37 | 14.55 | 1.13 | 0.0022 | RPL31 |
| TC1100007390.hg.1 | 7.78 | 7.96 | 1.13 | 0.0022 | EXT2 |
| TC0100011767.hg.1 | 8.92 | 9.1 | 1.13 | 0.0022 | SRP9 |
| TC0200008084.hg.1 | 7.74 | 7.91 | 1.13 | 0.0023 | TTC31 |
| TC0200016611.hg.1 | 5.53 | 5.71 | 1.13 | 0.0024 | GPR35 |
| TC2200008482.hg.1 | 9.5 | 9.68 | 1.13 | 0.0026 | EIF4ENIF1 |
| TC0800011239.hg.1 | 9.14 | 9.31 | 1.13 | 0.0028 | COX6C |
| TSUnmapped00000336.hg.1 | 11.35 | 11.53 | 1.13 | 0.0028 | RCC2 |
| TC0400010784.hg.1 | 9.97 | 10.15 | 1.13 | 0.0028 | NOA1 |
| TC1100009202.hg.1 | 12.35 | 12.53 | 1.13 | 0.0029 | CD3G |
| TC0100012895.hg.1 | 7.43 | 7.61 | 1.13 | 0.003 | MTHFR |
| TC1900011777.hg.1 | 9.57 | 9.74 | 1.13 | 0.003 | CYTH2 |
| TC0300010775.hg.1 | 15.88 | 16.05 | 1.13 | 0.003 | CX3CR1 |
| TC1600011225.hg.1 | 8.25 | 8.42 | 1.13 | 0.0031 | RNF166 |
| TC1600007893.hg.1 | 10.8 | 10.98 | 1.13 | 0.0032 | FTO |
| TC0700013420.hg.1 | 12.78 | 12.96 | 1.13 | 0.0034 | ARPC1B |
| TC0100009944.hg.1 | 10.65 | 10.83 | 1.13 | 0.0034 | PIP5K1A |
| TC1900008372.hg.1 | 10.4 | 10.58 | 1.13 | 0.0034 | PPP5C |
| TC1100009981.hg.1 | 12.14 | 12.32 | 1.13 | 0.0034 | TPP1 |
| TC1400009104.hg.1 | 13.56 | 13.74 | 1.13 | 0.0034 | RPL36AL |
| TC0600011517.hg.1 | 14.34 | 14.51 | 1.13 | 0.0034 | HLA-DPA1 |
| TC1900011071.hg.1 | 11.05 | 11.22 | 1.13 | 0.0034 | KDELR1 |
| TC1500008317.hg.1 | 9.84 | 10.01 | 1.13 | 0.0037 | CRTC3 |
| TC1800009245.hg.1 | 9.6 | 9.78 | 1.13 | 0.0038 | SERPINB8 |
| TC1900011707.hg.1 | 10 | 10.17 | 1.13 | 0.0038 | GPI |
| TC1300008614.hg.1 | 9.2 | 9.37 | 1.13 | 0.0038 | SPG20 |
| TC0200006687.hg.1 | 8.86 | 9.04 | 1.13 | 0.0038 | HPCAL1 |
| TC1100010946.hg.1 | 10.98 | 11.16 | 1.13 | 0.004 | LPXN |
| TC0500013426.hg.1 | 9.53 | 9.7 | 1.13 | 0.004 | TBC1D9B |
| TC1100013175.hg.1 | 8.77 | 8.95 | 1.13 | 0.004 | HNRNPUL2-BSCL2 |
| TC1600007484.hg.1 | 11.28 | 11.45 | 1.13 | 0.0042 | PRR14 |
| TC2200008609.hg.1 | 8.08 | 8.26 | 1.13 | 0.0042 | TXN2 |
| TC0100016035.hg.1 | 10.13 | 10.31 | 1.13 | 0.0042 | ETV3 |
| TC0200009281.hg.1 | 9.53 | 9.7 | 1.13 | 0.0042 | MZT2B |
| TC0600012550.hg.1 | 8.99 | 9.16 | 1.13 | 0.0043 | LYRM2 |
| TC0300007294.hg.1 | 5.88 | 6.05 | 1.13 | 0.0044 | PTPN23 |
| TC0300013104.hg.1 | 7.4 | 7.57 | 1.13 | 0.0046 | EIF5A2 |
| TC0100012870.hg.1 | 8.05 | 8.22 | 1.13 | 0.0047 | EXOSC10 |
| TC2000009259.hg.1 | 8.45 | 8.63 | 1.13 | 0.0047 | NCOA5 |
| TC0200009402.hg.1 | 7.96 | 8.13 | 1.13 | 0.0047 | MGAT5 |
| TC0100013184.hg.1 | 7.84 | 8.02 | 1.13 | 0.0048 | MUL1 |
| TC1700012401.hg.1 | 11.24 | 11.42 | 1.13 | 0.0048 | SMARCE1 |
| TC0100018418.hg.1 | 7.1 | 7.28 | 1.13 | 0.0048 | IFFO2 |
| TC1700008895.hg.1 | 7.17 | 7.34 | 1.13 | 0.005 | NUP85 |
| TC2200007404.hg.1 | 7.46 | 7.64 | 1.13 | 0.0051 | MIEF1 |
| TC1200008942.hg.1 | 8.78 | 8.96 | 1.13 | 0.0051 | PLBD2 |
| TC2000007620.hg.1 | 10.41 | 10.58 | 1.13 | 0.0051 | ARFGEF2 |
| TC0200012489.hg.1 | 6.58 | 6.76 | 1.13 | 0.0053 | PIGF |
| TC1900011392.hg.1 | 7.15 | 7.33 | 1.13 | 0.0053 | TFPT |
| TC1600011402.hg.1 | 9.72 | 9.9 | 1.13 | 0.0054 | POLR2C |
| TC0300009610.hg.1 | 10.35 | 10.53 | 1.13 | 0.0054 | NDUFB5 |
| TC1900007265.hg.1 | 8.8 | 8.97 | 1.13 | 0.0054 | FAM32A |
| TC0500013153.hg.1 | 9.53 | 9.7 | 1.13 | 0.0056 | MTRR |
| TC0900007547.hg.1 | 8.76 | 8.94 | 1.13 | 0.0056 | C9orf85 |
| TC1700009939.hg.1 | 5.77 | 5.95 | 1.13 | 0.0056 | PEMT |
| TC1600006539.hg.1 | 7.05 | 7.22 | 1.13 | 0.0058 | GNPTG |
| TC2000006576.hg.1 | 11.23 | 11.41 | 1.13 | 0.006 | PRNP |
| TC0X00011311.hg.1 | 5.49 | 5.66 | 1.13 | 0.006 | ARMCX5-GPRASP2 |
| TC0600013528.hg.1 | 11.94 | 12.13 | 1.13 | 0.0061 | LATS1 |
| TC0700008857.hg.1 | 9.81 | 9.99 | 1.13 | 0.0061 | MDFIC |
| TC2100007318.hg.1 | 8.34 | 8.52 | 1.13 | 0.0063 | RRP1B |
| TC1700008844.hg.1 | 8.81 | 8.99 | 1.13 | 0.0063 | TTYH2 |
| TC0300007256.hg.1 | 12.94 | 13.12 | 1.13 | 0.0067 | CCR2 |
| TC1900011141.hg.1 | 5.2 | 5.37 | 1.13 | 0.0067 | PIH1D1 |
| TC0900012168.hg.1 | 8.89 | 9.06 | 1.13 | 0.0067 | MRRF |
| TC0300007161.hg.1 | 8.45 | 8.63 | 1.13 | 0.0068 | VIPR1 |
| TC2100007474.hg.1 | 11.21 | 11.38 | 1.13 | 0.0068 | PRMT2 |
| TC1200007810.hg.1 | 9.05 | 9.22 | 1.13 | 0.0069 | ORMDL2 |
| TC1300008688.hg.1 | 8.21 | 8.39 | 1.13 | 0.0069 | FOXO1 |
| TC1800008128.hg.1 | 9.49 | 9.66 | 1.13 | 0.007 | PTPN2 |
| TC1100013091.hg.1 | 9.45 | 9.63 | 1.13 | 0.0073 | SDHD |
| TC1600011234.hg.1 | 6.59 | 6.77 | 1.13 | 0.0078 | APRT |
| TC1900007754.hg.1 | 7.43 | 7.61 | 1.13 | 0.0079 | NUDT19 |
| TC0200010796.hg.1 | 10.87 | 11.04 | 1.13 | 0.0081 | CTDSP1 |
| TC1900007695.hg.1 | 11.11 | 11.28 | 1.13 | 0.0084 | URI1 |
| TC0100015875.hg.1 | 12.09 | 12.27 | 1.13 | 0.0084 | ILF2 |
| TC0700009676.hg.1 | 14.55 | 14.73 | 1.13 | 0.0087 | GIMAP7 |
| TC2200007464.hg.1 | 7.42 | 7.6 | 1.13 | 0.0089 | L3MBTL2 |
| TC1700011736.hg.1 | 7.01 | 7.19 | 1.13 | 0.009 | NT5C |
| TC0X00008246.hg.1 | 6.76 | 6.93 | 1.13 | 0.0093 | SLC25A43 |
| TC2100007140.hg.1 | 9.83 | 10.01 | 1.13 | 0.0093 | ETS2 |
| TC1900007410.hg.1 | 8.14 | 8.32 | 1.13 | 0.0093 | UPF1 |
| TC0900011510.hg.1 | 14.5 | 14.67 | 1.13 | 0.0093 | RPL35 |
| TC1600009731.hg.1 | 9.14 | 9.31 | 1.13 | 0.0095 | COG7 |
| TC0900012179.hg.1 | 10.53 | 10.71 | 1.13 | 0.0098 | SPTAN1 |
| TC0200007591.hg.1 | 9.38 | 9.55 | 1.13 | 0.0098 | ERLEC1 |
| TC2200008099.hg.1 | 5.34 | 5.51 | 1.13 | 0.0099 | YDJC |
| TC0300006846.hg.1 | 15.88 | 16.06 | 1.14 | 6.76E-06 | RPL15 |
| TC1100013170.hg.1 | 17.17 | 17.36 | 1.14 | 1.79E-05 | EEF1G; MIR3654 |
| TC0100018199.hg.1 | 14.02 | 14.21 | 1.14 | 2.73E-05 | SRRM1 |
| TC1900008020.hg.1 | 13.63 | 13.82 | 1.14 | 2.73E-05 | ACTN4 |
| TC1700011418.hg.1 | 8.11 | 8.31 | 1.14 | 3.08E-05 | LIMD2 |
| TC1600007362.hg.1 | 13.61 | 13.8 | 1.14 | 3.81E-05 | ATXN2L |
| TC1700008180.hg.1 | 10.51 | 10.69 | 1.14 | 3.99E-05 | UBE2Z |
| TC0600011377.hg.1 | 11.2 | 11.39 | 1.14 | 8.52E-05 | NRM |
| TC1900006532.hg.1 | 17.05 | 17.25 | 1.14 | 9.54E-05 | RPS15 |
| TC2000009317.hg.1 | 12.88 | 13.07 | 1.14 | 9.78E-05 | SULF2 |
| TC0200012072.hg.1 | 12.01 | 12.21 | 1.14 | 0.0001 | MPV17 |
| TC2100008314.hg.1 | 12.04 | 12.23 | 1.14 | 0.0001 | CSTB |
| TC1200012640.hg.1 | 12.27 | 12.46 | 1.14 | 0.0002 | BLOC1S1 |
| TC0300013380.hg.1 | 12.11 | 12.3 | 1.14 | 0.0003 | ALG3 |
| TC0200014260.hg.1 | 8.64 | 8.83 | 1.14 | 0.0003 | CCDC115 |
| TC2000009014.hg.1 | 11.11 | 11.3 | 1.14 | 0.0003 | NDRG3 |
| TC1700011424.hg.1 | 11.31 | 11.49 | 1.14 | 0.0003 | SMARCD2 |
| TC1500010778.hg.1 | 10.57 | 10.75 | 1.14 | 0.0004 | IL16 |
| TC0600007599.hg.1 | 11.04 | 11.22 | 1.14 | 0.0004 | PRRC2A |
| TC0100011692.hg.1 | 11.24 | 11.43 | 1.14 | 0.0004 | MIA3 |
| TC1400009329.hg.1 | 8.89 | 9.08 | 1.14 | 0.0004 | RTN1 |
| TC1500009079.hg.1 | 12.38 | 12.57 | 1.14 | 0.0004 | SRP14 |
| TC1700006758.hg.1 | 10.97 | 11.15 | 1.14 | 0.0004 | MPDU1 |
| TC1000008574.hg.1 | 11.26 | 11.45 | 1.14 | 0.0004 | PGAM1 |
| TC1100009485.hg.1 | 14.04 | 14.23 | 1.14 | 0.0005 | FLI1 |
| TC0300012281.hg.1 | 9.77 | 9.96 | 1.14 | 0.0005 | ZXDC |
| TC1700007907.hg.1 | 7.41 | 7.59 | 1.14 | 0.0006 | TUBG2 |
| TC0100007659.hg.1 | 10.91 | 11.1 | 1.14 | 0.0006 | KHDRBS1 |
| TC1700009040.hg.1 | 11.58 | 11.77 | 1.14 | 0.0006 | SYNGR2 |
| TC1400008689.hg.1 | 5.4 | 5.58 | 1.14 | 0.0006 | PSMB5 |
| TC0400006689.hg.1 | 9.28 | 9.47 | 1.14 | 0.0008 | MAN2B2 |
| TC0600014276.hg.1 | 13.23 | 13.43 | 1.14 | 0.0008 | HLA-DMB |
| TC0800012392.hg.1 | 11.51 | 11.7 | 1.14 | 0.0008 | CNOT7 |
| TC1000012570.hg.1 | 8.83 | 9.02 | 1.14 | 0.0008 | NDST2 |
| TC0700008190.hg.1 | 13.3 | 13.49 | 1.14 | 0.0009 | CD36 |
| TC0100011192.hg.1 | 10.17 | 10.36 | 1.14 | 0.001 | IPO9 |
| TC1000008182.hg.1 | 10.29 | 10.48 | 1.14 | 0.001 | ZMIZ1 |
| TC0100009901.hg.1 | 12.23 | 12.42 | 1.14 | 0.0011 | MRPS21 |
| TC0X00011401.hg.1 | 12.48 | 12.66 | 1.14 | 0.0011 | RBMX; SNORD61 |
| TC0X00009441.hg.1 | 10.99 | 11.18 | 1.14 | 0.0012 | MED14 |
| TC1700012187.hg.1 | 10.64 | 10.83 | 1.14 | 0.0013 | TNFSF12-TNFSF13 |
| TC1400008341.hg.1 | 10.59 | 10.79 | 1.14 | 0.0014 | DYNC1H1 |
| TC0200014978.hg.1 | 10.9 | 11.09 | 1.14 | 0.0016 | OLA1 |
| TC0700009675.hg.1 | 9.68 | 9.87 | 1.14 | 0.0017 | GIMAP8 |
| TC1500010893.hg.1 | 8.75 | 8.93 | 1.14 | 0.0018 | PTPN9 |
| TC0700010035.hg.1 | 7.39 | 7.59 | 1.14 | 0.0018 | MAD1L1 |
| TC0500008558.hg.1 | 11.01 | 11.2 | 1.14 | 0.0018 | PRRC1 |
| TC1200010016.hg.1 | 11.91 | 12.1 | 1.14 | 0.0018 | WBP11 |
| TC0600014259.hg.1 | 11.4 | 11.59 | 1.14 | 0.0019 | ATP6V1G2-DDX39B |
| TC0500012247.hg.1 | 13.91 | 14.1 | 1.14 | 0.0019 | CD14 |
| TC1400008746.hg.1 | 10.82 | 11.01 | 1.14 | 0.0021 | TINF2 |
| TC1400008677.hg.1 | 11.52 | 11.71 | 1.14 | 0.0023 | SLC7A7 |
| TC0200013517.hg.1 | 11.76 | 11.95 | 1.14 | 0.0025 | SNRNP200 |
| TC1100011187.hg.1 | 8.22 | 8.4 | 1.14 | 0.0026 | MEN1 |
| TC1500010050.hg.1 | 10.46 | 10.64 | 1.14 | 0.0027 | COX5A |
| TC0800012280.hg.1 | 9.55 | 9.73 | 1.14 | 0.0027 | PDLIM2 |
| TC1400008752.hg.1 | 11.74 | 11.93 | 1.14 | 0.0027 | CIDEB |
| TC1600006574.hg.1 | 9.23 | 9.41 | 1.14 | 0.0028 | NDUFB10 |
| TSUnmapped00000095.hg.1 | 6.56 | 6.75 | 1.14 | 0.0029 | NDUFA6 |
| TC1100008330.hg.1 | 10.89 | 11.07 | 1.14 | 0.003 | IL18BP |
| TC0X00007424.hg.1 | 4.79 | 4.98 | 1.14 | 0.0031 | ZXDB |
| TC0200014192.hg.1 | 10.85 | 11.03 | 1.14 | 0.0033 | SAP130 |
| TC1000009527.hg.1 | 7.99 | 8.18 | 1.14 | 0.0033 | LARP4B |
| TC0700007409.hg.1 | 10.56 | 10.75 | 1.14 | 0.0036 | CCM2 |
| TC0700012917.hg.1 | 8.52 | 8.71 | 1.14 | 0.0036 | TPK1 |
| TC0X00011399.hg.1 | 7.47 | 7.66 | 1.14 | 0.0037 | FAM127B |
| TC1600007814.hg.1 | 9.84 | 10.02 | 1.14 | 0.0038 | ADCY7 |
| TC0500007048.hg.1 | 7.34 | 7.52 | 1.14 | 0.0039 | C5orf22 |
| TC1200006627.hg.1 | 7.54 | 7.73 | 1.14 | 0.0039 | NCAPD2 |
| TC1900007431.hg.1 | 8.93 | 9.12 | 1.14 | 0.0039 | MAU2 |
| TC0600013570.hg.1 | 8.43 | 8.61 | 1.14 | 0.004 | ZBTB2 |
| TC2000006561.hg.1 | 8.47 | 8.66 | 1.14 | 0.004 | MAVS |
| TC1700010811.hg.1 | 8.34 | 8.53 | 1.14 | 0.0041 | HDAC5 |
| TC0800012285.hg.1 | 8.57 | 8.77 | 1.14 | 0.0041 | HMBOX1 |
| TC1400009391.hg.1 | 8.23 | 8.42 | 1.14 | 0.0041 | WDR89 |
| TC2200008829.hg.1 | 7.21 | 7.39 | 1.14 | 0.0041 | POLR3H |
| TC1100010905.hg.1 | 6.48 | 6.67 | 1.14 | 0.0041 | MED19 |
| TC0300011222.hg.1 | 6.34 | 6.53 | 1.14 | 0.0042 | ACTR8 |
| TC1100009241.hg.1 | 9.17 | 9.35 | 1.14 | 0.0047 | HINFP |
| TC0200011237.hg.1 | 9.59 | 9.78 | 1.14 | 0.0048 | COPS8 |
| TC1400010022.hg.1 | 9.72 | 9.92 | 1.14 | 0.0048 | BTBD7 |
| TC0700011488.hg.1 | 8.43 | 8.62 | 1.14 | 0.005 | NSUN5 |
| TC0600013125.hg.1 | 11.3 | 11.48 | 1.14 | 0.0051 | THEMIS |
| TC1100011280.hg.1 | 7.4 | 7.59 | 1.14 | 0.0052 | YIF1A |
| TC0X00008136.hg.1 | 8.86 | 9.05 | 1.14 | 0.0052 | ALG13 |
| TC0300012384.hg.1 | 4.72 | 4.91 | 1.14 | 0.0053 | H1FX |
| TC0X00009762.hg.1 | 7.21 | 7.4 | 1.14 | 0.0054 | HSD17B10 |
| TC0600011375.hg.1 | 8.13 | 8.31 | 1.14 | 0.0054 | DHX16 |
| TC0300011038.hg.1 | 7.82 | 8.01 | 1.14 | 0.0057 | SLC25A20 |
| TC2200008370.hg.1 | 10.57 | 10.75 | 1.14 | 0.0057 | XBP1 |
| TC2100007331.hg.1 | 8 | 8.19 | 1.14 | 0.0057 | AGPAT3 |
| TC0X00008073.hg.1 | 10.07 | 10.25 | 1.14 | 0.0058 | PRPS1 |
| TC0700007807.hg.1 | 9.9 | 10.08 | 1.14 | 0.0058 | ZNF92 |
| TC1200009457.hg.1 | 8.11 | 8.3 | 1.14 | 0.0058 | ULK1 |
| TC1200007687.hg.1 | 14.41 | 14.59 | 1.14 | 0.0061 | EIF4B |
| TC0900008887.hg.1 | 8.64 | 8.83 | 1.14 | 0.0062 | TBC1D13 |
| TC0900011192.hg.1 | 9.05 | 9.24 | 1.14 | 0.0066 | LPAR1 |
| TC1100007940.hg.1 | 8.26 | 8.45 | 1.14 | 0.0067 | VEGFB |
| TC1600008888.hg.1 | 14.11 | 14.3 | 1.14 | 0.0067 | RPL13; SNORD68 |
| TC1100009168.hg.1 | 10.88 | 11.06 | 1.14 | 0.0068 | SIDT2 |
| TC0300012315.hg.1 | 7.92 | 8.11 | 1.14 | 0.0069 | TPRA1 |
| TC2200009272.hg.1 | 10.61 | 10.8 | 1.14 | 0.007 | APOBEC3D |
| TC0100015591.hg.1 | 8.61 | 8.8 | 1.14 | 0.007 | PIAS3 |
| TC0300011859.hg.1 | 8.8 | 8.99 | 1.14 | 0.007 | ZBTB11 |
| TC1100009674.hg.1 | 9.59 | 9.78 | 1.14 | 0.007 | RNH1 |
| TC0400009747.hg.1 | 6.32 | 6.51 | 1.14 | 0.0071 | TMEM129 |
| TC1700007899.hg.1 | 9.41 | 9.6 | 1.14 | 0.0072 | MLX |
| TC0500006803.hg.1 | 6.16 | 6.35 | 1.14 | 0.0077 | TRIO |
| TC0100012454.hg.1 | 9.62 | 9.81 | 1.14 | 0.0078 | SDF4 |
| TC0300012048.hg.1 | 11.4 | 11.59 | 1.14 | 0.008 | ZBTB20; MIR568 |
| TC0300008768.hg.1 | 8.08 | 8.27 | 1.14 | 0.008 | HMCES |
| TC1700010879.hg.1 | 8.21 | 8.39 | 1.14 | 0.0084 | MAP3K14 |
| TC1700009538.hg.1 | 10.59 | 10.78 | 1.14 | 0.0086 | SLC25A11 |
| TC1900008180.hg.1 | 8.37 | 8.55 | 1.14 | 0.0088 | ZNF526 |
| TC1200010655.hg.1 | 7.96 | 8.14 | 1.14 | 0.0089 | LIMA1 |
| TC1700008283.hg.1 | 7.46 | 7.65 | 1.14 | 0.0089 | UTP18 |
| TC1100008042.hg.1 | 5.14 | 5.33 | 1.14 | 0.0089 | CCDC85B |
| TC0500009061.hg.1 | 6.84 | 7.03 | 1.14 | 0.0093 | PCYOX1L |
| TC1500010045.hg.1 | 6.26 | 6.45 | 1.14 | 0.0093 | ULK3 |
| TC1700010732.hg.1 | 10.07 | 10.25 | 1.14 | 0.0096 | FAM134C |
| TC0200012642.hg.1 | 7.92 | 8.11 | 1.14 | 0.0097 | MTIF2 |
| TC1100008139.hg.1 | 9.1 | 9.28 | 1.14 | 0.0097 | AIP |
| TC0200008260.hg.1 | 10.37 | 10.56 | 1.14 | 0.0098 | VAMP8 |
| TC1100011338.hg.1 | 8.54 | 8.73 | 1.14 | 0.0099 | CDK2AP2 |
| TC1900007908.hg.1 | 15.42 | 15.62 | 1.15 | 9.09E-07 | CAPNS1 |
| TC0800012206.hg.1 | 11.86 | 12.06 | 1.15 | 6.59E-05 | DGAT1; MIR6848 |
| TC0100007334.hg.1 | 11.18 | 11.38 | 1.15 | 8.63E-05 | LYPLA2 |
| TC0100012465.hg.1 | 8.51 | 8.71 | 1.15 | 8.82E-05 | CPSF3L; MIR6727 |
| TC1700012190.hg.1 | 12.7 | 12.9 | 1.15 | 9.78E-05 | EIF4A1; SNORD10; SNORA67; SNORA48 |
| TC0100010066.hg.1 | 12.27 | 12.47 | 1.15 | 0.0001 | UBAP2L |
| TC1900011909.hg.1 | 12.71 | 12.91 | 1.15 | 0.0001 | COPE |
| TC2200008866.hg.1 | 8.07 | 8.26 | 1.15 | 0.0002 | TCF20 |
| TC0100010284.hg.1 | 10.93 | 11.13 | 1.15 | 0.0002 | PEA15 |
| TC0100013629.hg.1 | 7.53 | 7.73 | 1.15 | 0.0002 | YARS |
| TC0300013991.hg.1 | 9.64 | 9.85 | 1.15 | 0.0002 | TWF2 |
| TC1900006956.hg.1 | 8.37 | 8.57 | 1.15 | 0.0003 | PIN1 |
| TC1700009539.hg.1 | 16.14 | 16.35 | 1.15 | 0.0003 | PFN1 |
| TC1500010422.hg.1 | 9.59 | 9.79 | 1.15 | 0.0003 | IDH2 |
| TC0300010982.hg.1 | 9.71 | 9.91 | 1.15 | 0.0004 | SCAP |
| TC0200010502.hg.1 | 12.71 | 12.91 | 1.15 | 0.0004 | FAM117B |
| TC0300011109.hg.1 | 8.18 | 8.38 | 1.15 | 0.0004 | TMEM115 |
| TC1700009651.hg.1 | 11.01 | 11.21 | 1.15 | 0.0004 | TP53 |
| TC1700010199.hg.1 | 10.34 | 10.54 | 1.15 | 0.0004 | PIGS |
| TC0600011536.hg.1 | 13.67 | 13.86 | 1.15 | 0.0005 | TAPBP |
| TC1100008147.hg.1 | 9.22 | 9.43 | 1.15 | 0.0005 | NDUFV1 |
| TC1200007759.hg.1 | 11.57 | 11.77 | 1.15 | 0.0005 | COPZ1 |
| TC0900009164.hg.1 | 9.99 | 10.2 | 1.15 | 0.0005 | MRPS2 |
| TC0X00011414.hg.1 | 8.22 | 8.41 | 1.15 | 0.0006 | ARHGAP4 |
| TC0700009493.hg.1 | 12.27 | 12.47 | 1.15 | 0.0006 | ZYX |
| TC1700008923.hg.1 | 8.14 | 8.34 | 1.15 | 0.0006 | UNK |
| TC1200009416.hg.1 | 9.89 | 10.09 | 1.15 | 0.0006 | RAN |
| TC0200011386.hg.1 | 10.34 | 10.54 | 1.15 | 0.0007 | PPP1R7 |
| TC0100018393.hg.1 | 7.05 | 7.25 | 1.15 | 0.0007 | FAAP20 |
| TC2200009273.hg.1 | 9.93 | 10.13 | 1.15 | 0.0007 | APOBEC3F |
| TC1200012801.hg.1 | 12.81 | 13 | 1.15 | 0.0008 | CS |
| TC1000009111.hg.1 | 9.25 | 9.45 | 1.15 | 0.0009 | WDR11 |
| TC0100010123.hg.1 | 9.03 | 9.23 | 1.15 | 0.001 | FDPS |
| TC1400008749.hg.1 | 8.81 | 9.01 | 1.15 | 0.001 | RABGGTA |
| TC0200014288.hg.1 | 9.42 | 9.62 | 1.15 | 0.0011 | FAM168B |
| TC0300007164.hg.1 | 7.74 | 7.94 | 1.15 | 0.0011 | SS18L2 |
| TC1900006622.hg.1 | 8.09 | 8.29 | 1.15 | 0.0012 | NCLN |
| TC2000008915.hg.1 | 7.04 | 7.25 | 1.15 | 0.0012 | AHCY |
| TC2200008186.hg.1 | 9.29 | 9.49 | 1.15 | 0.0012 | CHCHD10 |
| TC0800008150.hg.1 | 9.3 | 9.49 | 1.15 | 0.0017 | CPNE3 |
| TC0300008467.hg.1 | 8.78 | 8.99 | 1.15 | 0.0017 | ARHGAP31 |
| TC0100007489.hg.1 | 7.45 | 7.66 | 1.15 | 0.0017 | NUDC |
| TC1900006890.hg.1 | 15.79 | 15.99 | 1.15 | 0.0018 | RPS28 |
| TC1700010982.hg.1 | 8.87 | 9.07 | 1.15 | 0.0018 | SKAP1 |
| TC0100018497.hg.1 | 9.81 | 10.01 | 1.15 | 0.0019 | SHC1 |
| TC0100017947.hg.1 | 7.19 | 7.4 | 1.15 | 0.002 | OPN3 |
| TC2200007971.hg.1 | 10.56 | 10.77 | 1.15 | 0.002 | DGCR14 |
| TC1200011936.hg.1 | 11.43 | 11.63 | 1.15 | 0.0022 | ATXN2 |
| TC1600008164.hg.1 | 10.32 | 10.52 | 1.15 | 0.0022 | FAM65A |
| TC0300013639.hg.1 | 10.36 | 10.56 | 1.15 | 0.0023 | PPP1R2 |
| TC1100013025.hg.1 | 8.49 | 8.69 | 1.15 | 0.0023 | TTC9C |
| TC0300006968.hg.1 | 11.14 | 11.35 | 1.15 | 0.0023 | CMTM7 |
| TC1300009273.hg.1 | 11.56 | 11.76 | 1.15 | 0.0024 | COMMD6 |
| TC1700010582.hg.1 | 7.2 | 7.41 | 1.15 | 0.0024 | PGAP3 |
| TSUnmapped00000349.hg.1 | 10.71 | 10.92 | 1.15 | 0.0026 | FBL |
| TC0100014660.hg.1 | 9.92 | 10.12 | 1.15 | 0.0026 | FUBP1 |
| TC2100008385.hg.1 | 9.86 | 10.06 | 1.15 | 0.0026 | SUMO3 |
| TC0100013548.hg.1 | 10.96 | 11.17 | 1.15 | 0.0027 | PUM1 |
| TC1100008041.hg.1 | 14.07 | 14.27 | 1.15 | 0.0029 | CTSW |
| TC1100010887.hg.1 | 8.99 | 9.19 | 1.15 | 0.003 | SSRP1 |
| TC1100011288.hg.1 | 7.39 | 7.59 | 1.15 | 0.003 | MRPL11 |
| TC0300011044.hg.1 | 7.97 | 8.16 | 1.15 | 0.0031 | DALRD3 |
| TC1900010391.hg.1 | 9.36 | 9.56 | 1.15 | 0.0031 | PEPD |
| TC0700012180.hg.1 | 7.25 | 7.44 | 1.15 | 0.0032 | SYPL1 |
| TC0X00007267.hg.1 | 7.19 | 7.4 | 1.15 | 0.0033 | MAGED1 |
| TC0900011259.hg.1 | 7.43 | 7.63 | 1.15 | 0.0035 | HDHD3 |
| TC0100012889.hg.1 | 8.29 | 8.48 | 1.15 | 0.0035 | MAD2L2 |
| TC0500012059.hg.1 | 10.35 | 10.55 | 1.15 | 0.0036 | VDAC1 |
| TC2000008211.hg.1 | 6.88 | 7.07 | 1.15 | 0.0037 | UBOX5; FASTKD5 |
| TC0500013064.hg.1 | 6.52 | 6.73 | 1.15 | 0.0037 | MGAT4B |
| TC1200007816.hg.1 | 9.03 | 9.24 | 1.15 | 0.0039 | DGKA |
| TC1900009872.hg.1 | 10.56 | 10.76 | 1.15 | 0.0039 | RASAL3 |
| TC0300012007.hg.1 | 7.15 | 7.35 | 1.15 | 0.004 | CD200R1 |
| TC0X00009750.hg.1 | 9.58 | 9.79 | 1.15 | 0.004 | KDM5C |
| TC0900007153.hg.1 | 8.65 | 8.86 | 1.15 | 0.0041 | GRHPR |
| TC1000012139.hg.1 | 7.8 | 8 | 1.15 | 0.0041 | CTBP2 |
| TC1900009408.hg.1 | 9.46 | 9.66 | 1.15 | 0.0042 | RANBP3 |
| TC0100015200.hg.1 | 5.49 | 5.69 | 1.15 | 0.0043 | AMIGO1 |
| TC0X00007923.hg.1 | 5.48 | 5.68 | 1.15 | 0.005 | CSTF2 |
| TC0100007303.hg.1 | 8.33 | 8.54 | 1.15 | 0.0052 | KDM1A; MIR3115 |
| TC1100008002.hg.1 | 8.92 | 9.12 | 1.15 | 0.0054 | MRPL49 |
| TC0700013055.hg.1 | 8.87 | 9.07 | 1.15 | 0.0056 | CDK5 |
| TC0900011302.hg.1 | 9.89 | 10.09 | 1.15 | 0.0061 | TNFSF8 |
| TC1900011752.hg.1 | 6.37 | 6.57 | 1.15 | 0.0061 | ZNF223 |
| TC0900007038.hg.1 | 6.44 | 6.64 | 1.15 | 0.0063 | NUDT2 |
| TSUnmapped00000054.hg.1 | 6.88 | 7.08 | 1.15 | 0.0063 | ZNF780B |
| TSUnmapped00000177.hg.1 | 6.88 | 7.08 | 1.15 | 0.0063 | ZNF780B |
| TC0200011219.hg.1 | 7.49 | 7.69 | 1.15 | 0.0063 | ACKR3 |
| TC0700011166.hg.1 | 12.57 | 12.77 | 1.15 | 0.0064 | CHCHD2 |
| TC1700009707.hg.1 | 7.09 | 7.29 | 1.15 | 0.0064 | PIK3R6 |
| TC0400009132.hg.1 | 6.99 | 7.2 | 1.15 | 0.0065 | ETFDH |
| TC0700010918.hg.1 | 7.85 | 8.04 | 1.15 | 0.0065 | NUDCD3 |
| TC1200010977.hg.1 | 9 | 9.2 | 1.15 | 0.0066 | CDK4 |
| TC2000006444.hg.1 | 6.14 | 6.34 | 1.15 | 0.0069 | TRIB3 |
| TC1700011435.hg.1 | 10.55 | 10.75 | 1.15 | 0.007 | ICAM2 |
| TC0700013601.hg.1 | 6.31 | 6.51 | 1.15 | 0.007 | RASA4B; RASA4 |
| TC0300014065.hg.1 | 8.26 | 8.46 | 1.15 | 0.0071 | SERP1 |
| TC0300008290.hg.1 | 12.4 | 12.6 | 1.15 | 0.0071 | TRAT1 |
| TC0100015783.hg.1 | 9.23 | 9.43 | 1.15 | 0.0072 | RFX5 |
| TC0100010193.hg.1 | 10.79 | 10.99 | 1.15 | 0.0078 | PRCC |
| TC0100011267.hg.1 | 12.23 | 12.43 | 1.15 | 0.0081 | ATP2B4 |
| TC1500006870.hg.1 | 7.51 | 7.71 | 1.15 | 0.0084 | C15orf41 |
| TC1600010647.hg.1 | 8.46 | 8.66 | 1.15 | 0.0084 | ACD |
| TC0200014870.hg.1 | 7.72 | 7.93 | 1.15 | 0.0086 | METTL5 |
| TC0100008126.hg.1 | 7.31 | 7.51 | 1.15 | 0.0087 | AKR1A1 |
| TC1000012066.hg.1 | 7.87 | 8.08 | 1.15 | 0.0088 | NSMCE4A |
| TC1900006537.hg.1 | 6.61 | 6.81 | 1.15 | 0.0089 | REEP6 |
| TC0200016702.hg.1 | 7.17 | 7.37 | 1.15 | 0.0091 | POLR1A |
| TC1900011798.hg.1 | 9.87 | 10.08 | 1.15 | 0.0095 | LILRA1 |
| TC0500010999.hg.1 | 10.2 | 10.41 | 1.16 | 6.76E-06 | TAF9; AK6 |
| TC1100013032.hg.1 | 10.98 | 11.19 | 1.16 | 3.33E-05 | DNAJC4 |
| TC0300009702.hg.1 | 9.52 | 9.73 | 1.16 | 4.87E-05 | EIF4G1 |
| TC1900009272.hg.1 | 14.48 | 14.7 | 1.16 | 5.39E-05 | AES |
| TC1200008573.hg.1 | 11.2 | 11.42 | 1.16 | 5.39E-05 | SLC25A3; SNORA53 |
| TC1900007906.hg.1 | 10.4 | 10.61 | 1.16 | 5.78E-05 | TBCB |
| TC1900009747.hg.1 | 11.06 | 11.27 | 1.16 | 6.83E-05 | C19orf43 |
| TC0700009485.hg.1 | 11.65 | 11.87 | 1.16 | 7.33E-05 | GSTK1 |
| TC1400007222.hg.1 | 11.48 | 11.69 | 1.16 | 7.77E-05 | MAPK1IP1L |
| TC1700008178.hg.1 | 12.62 | 12.83 | 1.16 | 8.62E-05 | ATP5G1 |
| TSUnmapped00000363.hg.1 | 9.83 | 10.05 | 1.16 | 0.0001 | EIF3F |
| TC0X00008860.hg.1 | 12.67 | 12.88 | 1.16 | 0.0001 | F8A2; F8A3; F8A1 |
| TC0300007312.hg.1 | 8.26 | 8.48 | 1.16 | 0.0001 | DHX30 |
| TC1100012514.hg.1 | 10.66 | 10.87 | 1.16 | 0.0001 | DPAGT1 |
| TC1900010941.hg.1 | 8.26 | 8.48 | 1.16 | 0.0001 | ERCC1 |
| TC0100015715.hg.1 | 13.25 | 13.47 | 1.16 | 0.0001 | SF3B4 |
| TC1700012474.hg.1 | 10.05 | 10.27 | 1.16 | 0.0002 | EXOC7; MIR6868 |
| TC0900012184.hg.1 | 8.24 | 8.45 | 1.16 | 0.0002 | PRRC2B |
| TC1700011970.hg.1 | 8.73 | 8.94 | 1.16 | 0.0002 | SGSH |
| TC1600011527.hg.1 | 12.5 | 12.71 | 1.16 | 0.0002 | VKORC1 |
| TC1900009806.hg.1 | 10.79 | 11 | 1.16 | 0.0002 | PRKACA |
| TC0200013265.hg.1 | 9.49 | 9.71 | 1.16 | 0.0003 | CAPG |
| TC0300010664.hg.1 | 11.39 | 11.61 | 1.16 | 0.0003 | GLB1; TMPPE |
| TC0100012901.hg.1 | 8.25 | 8.47 | 1.16 | 0.0003 | KIAA2013 |
| TC1900007270.hg.1 | 14.35 | 14.57 | 1.16 | 0.0003 | KLF2 |
| TC0100018498.hg.1 | 12.05 | 12.25 | 1.16 | 0.0004 | KRTCAP2 |
| TC0100010104.hg.1 | 11.18 | 11.4 | 1.16 | 0.0004 | ZBTB7B |
| TSUnmapped00000264.hg.1 | 14.45 | 14.66 | 1.16 | 0.0005 | RPL7A |
| TC0100015596.hg.1 | 9.95 | 10.17 | 1.16 | 0.0005 | LIX1L |
| TC0500011490.hg.1 | 9.1 | 9.31 | 1.16 | 0.0006 | TTC37 |
| TC0800008370.hg.1 | 9.27 | 9.48 | 1.16 | 0.0006 | POLR2K |
| TC0500012248.hg.1 | 8.98 | 9.19 | 1.16 | 0.0007 | NDUFA2 |
| TC1100013039.hg.1 | 6.75 | 6.97 | 1.16 | 0.0007 | ARL2-SNX15 |
| TC0300008604.hg.1 | 5.96 | 6.17 | 1.16 | 0.0008 | UMPS |
| TC1200008535.hg.1 | 8.13 | 8.35 | 1.16 | 0.0008 | ELK3 |
| TC0300014043.hg.1 | 9.11 | 9.33 | 1.16 | 0.0009 | ISY1 |
| TC0700012165.hg.1 | 9.02 | 9.24 | 1.16 | 0.0009 | ATXN7L1 |
| TC0200013515.hg.1 | 12.56 | 12.77 | 1.16 | 0.001 | STARD7 |
| TC0700007633.hg.1 | 9.48 | 9.7 | 1.16 | 0.001 | SUMF2 |
| TC1900007331.hg.1 | 10.31 | 10.52 | 1.16 | 0.001 | COLGALT1 |
| TC1900010076.hg.1 | 10.07 | 10.29 | 1.16 | 0.0011 | ATP13A1 |
| TC1100011035.hg.1 | 10.35 | 10.57 | 1.16 | 0.0012 | CPSF7 |
| TC0700006567.hg.1 | 9.8 | 10 | 1.16 | 0.0012 | TTYH3 |
| TC0900012011.hg.1 | 9.61 | 9.82 | 1.16 | 0.0013 | EDF1 |
| TC0100013087.hg.1 | 9.86 | 10.08 | 1.16 | 0.0013 | RCC2 |
| TC2100007341.hg.1 | 6.04 | 6.26 | 1.16 | 0.0014 | PWP2 |
| TC0800007419.hg.1 | 8.45 | 8.67 | 1.16 | 0.0014 | GPAT4 |
| TC1900007864.hg.1 | 8.51 | 8.72 | 1.16 | 0.0015 | TMEM147 |
| TC1700007976.hg.1 | 10.72 | 10.94 | 1.16 | 0.0016 | G6PC3 |
| TC1900008084.hg.1 | 8.78 | 8.99 | 1.16 | 0.0016 | PSMC4 |
| TC1100010546.hg.1 | 7.87 | 8.09 | 1.16 | 0.0018 | COMMD9 |
| TC1100011161.hg.1 | 10.62 | 10.83 | 1.16 | 0.0019 | TRMT112 |
| TSUnmapped00000776.hg.1 | 11.16 | 11.38 | 1.16 | 0.0019 | FBL |
| TC0700013500.hg.1 | 8.15 | 8.36 | 1.16 | 0.0019 | ADAP1 |
| TC0200016366.hg.1 | 7.56 | 7.78 | 1.16 | 0.0019 | THAP4 |
| TC1800008584.hg.1 | 10.38 | 10.59 | 1.16 | 0.002 | ATP5A1 |
| TC0300009789.hg.1 | 11.85 | 12.06 | 1.16 | 0.002 | ST6GAL1 |
| TC1700012191.hg.1 | 13.99 | 14.19 | 1.16 | 0.0021 | CD68 |
| TSUnmapped00000162.hg.1 | 13.04 | 13.25 | 1.16 | 0.0021 | FBL |
| TC0200013746.hg.1 | 7.91 | 8.13 | 1.16 | 0.0022 | TGFBRAP1 |
| TC1100011010.hg.1 | 8.66 | 8.87 | 1.16 | 0.0023 | PRPF19 |
| TC1700008564.hg.1 | 6.92 | 7.14 | 1.16 | 0.0025 | TACO1 |
| TC1100009200.hg.1 | 15.05 | 15.27 | 1.16 | 0.0026 | CD3E |
| TC1100013230.hg.1 | 11.27 | 11.48 | 1.16 | 0.0026 | BCL9L |
| TC0200015329.hg.1 | 7.13 | 7.35 | 1.16 | 0.0026 | GTF3C3 |
| TC2200008903.hg.1 | 7.19 | 7.41 | 1.16 | 0.0027 | MCAT |
| TC0700012734.hg.1 | 7.91 | 8.12 | 1.16 | 0.0027 | CREB3L2 |
| TC0400012078.hg.1 | 7.48 | 7.7 | 1.16 | 0.0027 | NR3C2 |
| TC1000008221.hg.1 | 7.61 | 7.81 | 1.16 | 0.0028 | TMEM254 |
| TC0100015952.hg.1 | 9.79 | 10 | 1.16 | 0.0029 | CLK2 |
| TC1900007436.hg.1 | 9.87 | 10.08 | 1.16 | 0.0029 | GATAD2A; MIR640 |
| TC2200009157.hg.1 | 7.68 | 7.89 | 1.16 | 0.0033 | SBF1 |
| TC1200011870.hg.1 | 8.73 | 8.94 | 1.16 | 0.0034 | KCTD10 |
| TC0900007098.hg.1 | 7.56 | 7.78 | 1.16 | 0.0036 | RGP1 |
| TC1500006972.hg.1 | 9.16 | 9.37 | 1.16 | 0.0039 | SPINT1 |
| TC2000009458.hg.1 | 11.08 | 11.3 | 1.16 | 0.0039 | NFATC2 |
| TC2000008218.hg.1 | 8.48 | 8.69 | 1.16 | 0.004 | C20orf194 |
| TC0100013153.hg.1 | 6.14 | 6.35 | 1.16 | 0.0041 | TMCO4 |
| TC1000011592.hg.1 | 7.81 | 8.02 | 1.16 | 0.0041 | COX15 |
| TC1200006640.hg.1 | 6.81 | 7.03 | 1.16 | 0.0043 | COPS7A |
| TC0100010314.hg.1 | 8.97 | 9.18 | 1.16 | 0.0046 | LY9 |
| TC1900009600.hg.1 | 6.64 | 6.85 | 1.16 | 0.0046 | FBXL12 |
| TC2200007776.hg.1 | 7.72 | 7.94 | 1.16 | 0.0047 | ZBED4 |
| TC0300006483.hg.1 | 12.02 | 12.23 | 1.16 | 0.0047 | BHLHE40 |
| TC2200009282.hg.1 | 9.37 | 9.58 | 1.16 | 0.005 | SAMM50 |
| TC1000007701.hg.1 | 9.24 | 9.46 | 1.16 | 0.0051 | TFAM |
| TC0700013469.hg.1 | 9.9 | 10.11 | 1.16 | 0.0054 | SSBP1 |
| TC1400009766.hg.1 | 5.86 | 6.08 | 1.16 | 0.0054 | IRF2BPL |
| TC1700008907.hg.1 | 6.45 | 6.65 | 1.16 | 0.0055 | TMEM94; MIR6785 |
| TC1000008769.hg.1 | 6.72 | 6.94 | 1.16 | 0.0056 | TAF5 |
| TC0700011499.hg.1 | 5.13 | 5.34 | 1.16 | 0.0056 | DNAJC30 |
| TC1200010109.hg.1 | 13.33 | 13.55 | 1.16 | 0.0057 | LDHB |
| TC1200009587.hg.1 | 7.3 | 7.52 | 1.16 | 0.0057 | CACNA2D4 |
| TC0600008099.hg.1 | 7.63 | 7.84 | 1.16 | 0.0058 | POLH |
| TC0300007383.hg.1 | 7.21 | 7.42 | 1.16 | 0.0064 | DAG1 |
| TC0400009919.hg.1 | 10.67 | 10.89 | 1.16 | 0.0064 | MRFAP1L1 |
| TC1900011632.hg.1 | 6.93 | 7.14 | 1.16 | 0.0067 | ZBTB45 |
| TC1600011373.hg.1 | 7.56 | 7.77 | 1.16 | 0.0068 | SLX1A-SULT1A3 |
| TC0900010866.hg.1 | 8.3 | 8.52 | 1.16 | 0.007 | FBP1 |
| TC0600011870.hg.1 | 6.53 | 6.74 | 1.16 | 0.0073 | DNPH1 |
| TC0200010157.hg.1 | 8.79 | 9 | 1.16 | 0.0074 | ITGA4 |
| TC0800010783.hg.1 | 7.67 | 7.89 | 1.16 | 0.0076 | MSC |
| TC0200016474.hg.1 | 14.98 | 15.19 | 1.16 | 0.0078 | NAGK |
| TC2000008951.hg.1 | 6.76 | 6.96 | 1.16 | 0.0085 | GSS |
| TC0900008380.hg.1 | 7.32 | 7.54 | 1.16 | 0.0086 | FAM206A |
| TC1100011258.hg.1 | 7.9 | 8.11 | 1.16 | 0.0089 | FIBP |
| TC1700009623.hg.1 | 7.98 | 8.18 | 1.16 | 0.0091 | PHF23 |
| TC1900007975.hg.1 | 7.04 | 7.26 | 1.16 | 0.0091 | ZNF570 |
| TC1500010725.hg.1 | 6.62 | 6.83 | 1.16 | 0.0093 | CAPN3 |
| TC1100011331.hg.1 | 14.98 | 15.21 | 1.17 | 9.29E-08 | PPP1CA |
| TC0200012030.hg.1 | 12.45 | 12.68 | 1.17 | 6.57E-06 | HADHA |
| TC2200006623.hg.1 | 10.21 | 10.43 | 1.17 | 1.74E-05 | COMT; MIR4761 |
| TC1600007470.hg.1 | 11.97 | 12.2 | 1.17 | 3.11E-05 | ITGAL |
| TC0700011550.hg.1 | 13.28 | 13.51 | 1.17 | 6.50E-05 | POM121C |
| TC1600011220.hg.1 | 12.79 | 13.01 | 1.17 | 7.06E-05 | CYBA |
| TC0500012182.hg.1 | 10.68 | 10.91 | 1.17 | 0.0001 | HSPA9 |
| TC1200010798.hg.1 | 10.96 | 11.18 | 1.17 | 0.0001 | AAAS |
| TC0600011443.hg.1 | 7.99 | 8.22 | 1.17 | 0.0001 | GPANK1 |
| TC2200008856.hg.1 | 10.31 | 10.54 | 1.17 | 0.0002 | NAGA |
| TC1900011981.hg.1 | 7.35 | 7.58 | 1.17 | 0.0002 | ERCC2 |
| TC0X00009580.hg.1 | 10.13 | 10.36 | 1.17 | 0.0003 | CFP |
| TC2000007488.hg.1 | 9.49 | 9.71 | 1.17 | 0.0003 | PIGT |
| TC2100007355.hg.1 | 9.72 | 9.94 | 1.17 | 0.0003 | PFKL |
| TC0100016145.hg.1 | 9.12 | 9.34 | 1.17 | 0.0004 | CD244 |
| TC1400007695.hg.1 | 9.08 | 9.3 | 1.17 | 0.0004 | EIF2B2 |
| TC1700010214.hg.1 | 5.52 | 5.75 | 1.17 | 0.0005 | FAM222B |
| TC2100008561.hg.1 | 12.33 | 12.56 | 1.17 | 0.0005 | ATP5O |
| TC1000010725.hg.1 | 13.22 | 13.45 | 1.17 | 0.0005 | CCDC6 |
| TC1300009933.hg.1 | 10.53 | 10.76 | 1.17 | 0.0005 | RASA3 |
| TC2000010010.hg.1 | 9.07 | 9.29 | 1.17 | 0.0006 | BLCAP |
| TC1900009128.hg.1 | 9.78 | 10 | 1.17 | 0.0006 | TMEM259 |
| TC1700008867.hg.1 | 12.25 | 12.47 | 1.17 | 0.0006 | SLC9A3R1; MIR3615 |
| TC0100014050.hg.1 | 9.01 | 9.24 | 1.17 | 0.0006 | LRRC41 |
| TC1900011675.hg.1 | 11.1 | 11.32 | 1.17 | 0.0007 | C19orf53 |
| TC1700011045.hg.1 | 11.28 | 11.51 | 1.17 | 0.0007 | PHB |
| TC1500008286.hg.1 | 9.27 | 9.49 | 1.17 | 0.0007 | ZNF710 |
| TC1100007935.hg.1 | 10.42 | 10.64 | 1.17 | 0.0007 | STIP1 |
| TC2200008661.hg.1 | 8.81 | 9.03 | 1.17 | 0.0007 | LGALS2 |
| TC0100011374.hg.1 | 8.82 | 9.05 | 1.17 | 0.001 | IKBKE |
| TC1400009548.hg.1 | 9.1 | 9.32 | 1.17 | 0.001 | ERH |
| TC0300006491.hg.1 | 9.49 | 9.71 | 1.17 | 0.0011 | EDEM1 |
| TC0500013015.hg.1 | 8.53 | 8.76 | 1.17 | 0.0011 | PHYKPL |
| TC0700011575.hg.1 | 9.67 | 9.89 | 1.17 | 0.0012 | POMZP3 |
| TC0800007316.hg.1 | 7.99 | 8.21 | 1.17 | 0.0012 | EIF4EBP1 |
| TC0600006836.hg.1 | 12.64 | 12.87 | 1.17 | 0.0012 | LY86 |
| TC1900011150.hg.1 | 8.67 | 8.9 | 1.17 | 0.0013 | NOSIP |
| TC0300013989.hg.1 | 9.11 | 9.34 | 1.17 | 0.0013 | ABHD14B |
| TC1200009172.hg.1 | 8.94 | 9.17 | 1.17 | 0.0014 | ORAI1 |
| TC1900006866.hg.1 | 7.16 | 7.39 | 1.17 | 0.0015 | SNAPC2 |
| TC0300006993.hg.1 | 14.01 | 14.24 | 1.17 | 0.0016 | CRTAP |
| TC0X00008911.hg.1 | 11.09 | 11.32 | 1.17 | 0.0017 | P2RY8 |
| TC0Y00006887.hg.1 | 11.09 | 11.32 | 1.17 | 0.0017 | P2RY8 |
| TC0500012498.hg.1 | 11.69 | 11.91 | 1.17 | 0.0017 | ANXA6 |
| TC0700006650.hg.1 | 8.48 | 8.7 | 1.17 | 0.0018 | C7orf26 |
| TC1000009844.hg.1 | 7.73 | 7.97 | 1.17 | 0.0018 | SEPHS1 |
| TC1200009467.hg.1 | 7.9 | 8.12 | 1.17 | 0.0019 | NOC4L |
| TC1100006634.hg.1 | 6.29 | 6.52 | 1.17 | 0.002 | PGAP2 |
| TC1100007168.hg.1 | 9.49 | 9.72 | 1.17 | 0.002 | ARL14EP |
| TC2200007206.hg.1 | 7.9 | 8.13 | 1.17 | 0.0021 | MCM5 |
| TC1900009320.hg.1 | 10.33 | 10.55 | 1.17 | 0.0022 | ZBTB7A |
| TC1700006733.hg.1 | 7.92 | 8.15 | 1.17 | 0.0022 | ELP5 |
| TC0200016680.hg.1 | 6.47 | 6.7 | 1.17 | 0.0022 | SERTAD2 |
| TC0100008938.hg.1 | 8.05 | 8.28 | 1.17 | 0.0023 | LMO4 |
| TC2200008837.hg.1 | 10.62 | 10.84 | 1.17 | 0.0024 | SNU13 |
| TC0700008524.hg.1 | 4.88 | 5.1 | 1.17 | 0.0024 | CNPY4 |
| TC0800012176.hg.1 | 8.71 | 8.94 | 1.17 | 0.0026 | PLEC |
| TC0100013072.hg.1 | 7.5 | 7.73 | 1.17 | 0.0027 | ATP13A2 |
| TC1700010436.hg.1 | 6.96 | 7.19 | 1.17 | 0.0027 | PEX12 |
| TC0900011603.hg.1 | 10.88 | 11.11 | 1.17 | 0.0028 | ENG |
| TC1600010604.hg.1 | 7.79 | 8.02 | 1.17 | 0.003 | FAM96B |
| TC1200012597.hg.1 | 9.91 | 10.13 | 1.17 | 0.003 | MGST1 |
| TC2000008104.hg.1 | 7.9 | 8.12 | 1.17 | 0.003 | PRPF6 |
| TC1900008435.hg.1 | 8.24 | 8.47 | 1.17 | 0.003 | SEPW1 |
| TC0200012261.hg.1 | 8.58 | 8.81 | 1.17 | 0.0031 | PRKD3 |
| TC1100010893.hg.1 | 7.3 | 7.53 | 1.17 | 0.0033 | SLC43A1 |
| TC0200007049.hg.1 | 9.4 | 9.62 | 1.17 | 0.0035 | TMEM214 |
| TC1700012260.hg.1 | 9.14 | 9.37 | 1.17 | 0.0035 | COASY |
| TC1300009288.hg.1 | 6.96 | 7.19 | 1.17 | 0.0035 | KCTD12 |
| TC0100008383.hg.1 | 10.01 | 10.24 | 1.17 | 0.0036 | MRPL37 |
| TC0100013278.hg.1 | 6.65 | 6.88 | 1.17 | 0.0037 | TCEA3 |
| TC1500007633.hg.1 | 9.68 | 9.91 | 1.17 | 0.0037 | SMAD3 |
| TC1900011155.hg.1 | 4.4 | 4.62 | 1.17 | 0.0037 | IRF3 |
| TC1900009766.hg.1 | 8.52 | 8.75 | 1.17 | 0.0039 | TRMT1 |
| TC1600009381.hg.1 | 8.09 | 8.32 | 1.17 | 0.0039 | DEXI |
| TC0100010798.hg.1 | 10.22 | 10.44 | 1.17 | 0.004 | QSOX1 |
| TC0300010035.hg.1 | 7.2 | 7.43 | 1.17 | 0.0041 | SENP5 |
| TC0200011990.hg.1 | 9.21 | 9.44 | 1.17 | 0.0044 | PTRHD1 |
| TC0100010661.hg.1 | 7.98 | 8.21 | 1.17 | 0.0044 | DARS2 |
| TC0400008348.hg.1 | 7.41 | 7.64 | 1.17 | 0.0046 | HADH |
| TC1900011840.hg.1 | 7.18 | 7.4 | 1.17 | 0.0046 | TIMM13 |
| TC2200007490.hg.1 | 7.41 | 7.63 | 1.17 | 0.0057 | MEI1 |
| TC1200009157.hg.1 | 8.27 | 8.5 | 1.17 | 0.0062 | P2RX7 |
| TC0600007687.hg.1 | 6.38 | 6.61 | 1.17 | 0.0065 | RING1 |
| TC1500007601.hg.1 | 8.33 | 8.56 | 1.17 | 0.0065 | HACD3 |
| TC2200009234.hg.1 | 9.34 | 9.57 | 1.17 | 0.007 | SPECC1L-ADORA2A |
| TC1900006796.hg.1 | 5.46 | 5.69 | 1.17 | 0.0071 | ALKBH7 |
| TC0X00008716.hg.1 | 6.4 | 6.62 | 1.17 | 0.0071 | MTM1 |
| TC0200008259.hg.1 | 11.17 | 11.4 | 1.17 | 0.0073 | MAT2A |
| TC1700010630.hg.1 | 15.14 | 15.36 | 1.17 | 0.0076 | CCR7 |
| TC0900008969.hg.1 | 7.05 | 7.28 | 1.17 | 0.0081 | ABL1 |
| TC1200008525.hg.1 | 5.79 | 6.02 | 1.17 | 0.0084 | SNRPF |
| TC0600011808.hg.1 | 6.32 | 6.56 | 1.17 | 0.0084 | MED20 |
| TC2000008200.hg.1 | 8.05 | 8.27 | 1.17 | 0.0094 | PCED1A |
| TC1900007358.hg.1 | 8.47 | 8.7 | 1.17 | 0.0099 | ARRDC2 |
| TC2200008654.hg.1 | 12.16 | 12.4 | 1.18 | 1.10E-06 | MFNG |
| TC1200012798.hg.1 | 13.78 | 14.02 | 1.18 | 2.31E-06 | ATP5G2 |
| TC0300008514.hg.1 | 10.12 | 10.37 | 1.18 | 1.01E-05 | NDUFB4 |
| TC0800009247.hg.1 | 8.25 | 8.5 | 1.18 | 1.23E-05 | HSF1 |
| TC1700012329.hg.1 | 6.96 | 7.21 | 1.18 | 2.73E-05 | METTL16 |
| TC2200007963.hg.1 | 8.84 | 9.08 | 1.18 | 4.02E-05 | DGCR2; DGCR11 |
| TC0400012310.hg.1 | 11.15 | 11.39 | 1.18 | 4.32E-05 | 01-מרץ |
| TC0700006603.hg.1 | 8.8 | 9.04 | 1.18 | 7.73E-05 | FOXK1 |
| TC0500008736.hg.1 | 12.38 | 12.62 | 1.18 | 7.77E-05 | TGFBI |
| TC1200012151.hg.1 | 13.01 | 13.25 | 1.18 | 8.62E-05 | RPLP0 |
| TC0100006852.hg.1 | 10.92 | 11.15 | 1.18 | 9.49E-05 | UBIAD1 |
| TC1400006519.hg.1 | 11.84 | 12.08 | 1.18 | 0.0001 | APEX1 |
| TC1400008193.hg.1 | 11.98 | 12.22 | 1.18 | 0.0001 | EVL |
| TC1700012109.hg.1 | 9.17 | 9.41 | 1.18 | 0.0001 | CD7 |
| TC1900011990.hg.1 | 16.18 | 16.42 | 1.18 | 0.0002 | RPL18 |
| TC1100013165.hg.1 | 10.65 | 10.9 | 1.18 | 0.0002 | SLC43A3 |
| TC1000007396.hg.1 | 7.71 | 7.95 | 1.18 | 0.0002 | BMS1 |
| TC1900006976.hg.1 | 6.17 | 6.42 | 1.18 | 0.0002 | MRPL4 |
| TC1200006645.hg.1 | 12.72 | 12.96 | 1.18 | 0.0002 | CD4 |
| TC0900011980.hg.1 | 7.05 | 7.29 | 1.18 | 0.0003 | SEC16A |
| TC1700006480.hg.1 | 9.43 | 9.67 | 1.18 | 0.0003 | TIMM22 |
| TC0100008103.hg.1 | 14.11 | 14.35 | 1.18 | 0.0003 | RPS8; SNORD55; SNORD46; SNORD38A; SNORD38B |
| TC1700007399.hg.1 | 7.21 | 7.45 | 1.18 | 0.0003 | ERAL1 |
| TC0800007086.hg.1 | 11.65 | 11.89 | 1.18 | 0.0004 | DPYSL2 |
| TC2100008555.hg.1 | 8.38 | 8.61 | 1.18 | 0.0004 | TIAM1 |
| TC0X00011181.hg.1 | 9.39 | 9.63 | 1.18 | 0.0004 | HCFC1 |
| TC0200015012.hg.1 | 8.8 | 9.04 | 1.18 | 0.0004 | ATP5G3 |
| TC0900012212.hg.1 | 8.56 | 8.8 | 1.18 | 0.0005 | PLIN2 |
| TC1600007514.hg.1 | 8.87 | 9.11 | 1.18 | 0.0005 | KAT8 |
| TC1100011395.hg.1 | 8.23 | 8.47 | 1.18 | 0.0006 | CPT1A |
| TC1600008712.hg.1 | 10.54 | 10.78 | 1.18 | 0.0006 | IRF8 |
| TC1100011737.hg.1 | 9.63 | 9.87 | 1.18 | 0.0007 | NDUFC2-KCTD14; NDUFC2; KCTD14 |
| TC0600007677.hg.1 | 16.33 | 16.57 | 1.18 | 0.0007 | HLA-DPB1 |
| TC0300011029.hg.1 | 9.26 | 9.49 | 1.18 | 0.0007 | UQCRC1 |
| TC1700011084.hg.1 | 7.66 | 7.91 | 1.18 | 0.0008 | PPP1R9B |
| TC0500012163.hg.1 | 6.65 | 6.89 | 1.18 | 0.0008 | CDC23 |
| TC0600011546.hg.1 | 10.7 | 10.93 | 1.18 | 0.0009 | CUTA |
| TC0100015810.hg.1 | 9.81 | 10.05 | 1.18 | 0.0012 | MRPL9 |
| TC1000007761.hg.1 | 11.72 | 11.96 | 1.18 | 0.0013 | ARID5B |
| TC1700007997.hg.1 | 13.81 | 14.05 | 1.18 | 0.0013 | GRN |
| TC0800009988.hg.1 | 8.24 | 8.48 | 1.18 | 0.0014 | INTS9 |
| TC0200016589.hg.1 | 6.22 | 6.45 | 1.18 | 0.0015 | C2orf69 |
| TC0200016787.hg.1 | 8.59 | 8.83 | 1.18 | 0.0019 | NDUFA10 |
| TC1600009420.hg.1 | 12.41 | 12.65 | 1.18 | 0.0019 | RSL1D1 |
| TC0500013316.hg.1 | 12.18 | 12.42 | 1.18 | 0.0022 | CD180 |
| TC0100006771.hg.1 | 8.9 | 9.14 | 1.18 | 0.0023 | H6PD |
| TC0100017110.hg.1 | 14.98 | 15.23 | 1.18 | 0.0023 | FCMR |
| TC1600008189.hg.1 | 8.33 | 8.56 | 1.18 | 0.0023 | PLA2G15 |
| TC0100018299.hg.1 | 7.93 | 8.17 | 1.18 | 0.0026 | SLC27A3 |
| TC0X00010136.hg.1 | 7.81 | 8.05 | 1.18 | 0.0028 | TAF9B |
| TC0X00011277.hg.1 | 12.3 | 12.54 | 1.18 | 0.0029 | CA5B |
| TC0100011770.hg.1 | 6.4 | 6.64 | 1.18 | 0.003 | EPHX1 |
| TC0900009260.hg.1 | 5.65 | 5.89 | 1.18 | 0.0031 | MAN1B1 |
| TC1400010156.hg.1 | 6.99 | 7.23 | 1.18 | 0.0031 | BCL11B |
| TC1000012587.hg.1 | 9.57 | 9.81 | 1.18 | 0.0032 | NDUFB8 |
| TC0500007542.hg.1 | 6.59 | 6.83 | 1.18 | 0.0033 | NDUFAF2 |
| TC1900007679.hg.1 | 8.3 | 8.54 | 1.18 | 0.0039 | PLEKHF1 |
| TC1700009809.hg.1 | 6.93 | 7.17 | 1.18 | 0.0041 | HS3ST3A1 |
| TC0400008170.hg.1 | 7.77 | 8.01 | 1.18 | 0.0044 | SMARCAD1 |
| TC0100016027.hg.1 | 7.09 | 7.33 | 1.18 | 0.0046 | ARHGEF11 |
| TC1000008712.hg.1 | 7.24 | 7.48 | 1.18 | 0.0053 | PPRC1 |
| TC0300013866.hg.1 | 7.44 | 7.67 | 1.18 | 0.0054 | ALG1L2 |
| TC0X00010172.hg.1 | 5.65 | 5.9 | 1.18 | 0.0062 | HMGN5 |
| TC1600006587.hg.1 | 7.27 | 7.5 | 1.18 | 0.0067 | TSC2 |
| TC1000010727.hg.1 | 9.09 | 9.33 | 1.18 | 0.0069 | ANK3 |
| TC1700010488.hg.1 | 6.69 | 6.94 | 1.18 | 0.0073 | ACACA |
| TC1600008973.hg.1 | 7.28 | 7.52 | 1.18 | 0.0074 | FBXL16 |
| TC0200007195.hg.1 | 6.65 | 6.89 | 1.18 | 0.0076 | TTC27; MIR4765 |
| TC0600007540.hg.1 | 8.99 | 9.23 | 1.18 | 0.008 | C6orf136 |
| TC0400007311.hg.1 | 9.65 | 9.89 | 1.18 | 0.0081 | RHOH |
| TC0X00009254.hg.1 | 5.94 | 6.18 | 1.18 | 0.0084 | APOO |
| TC1600009837.hg.1 | 5.86 | 6.1 | 1.18 | 0.009 | GTF3C1 |
| TC2200008055.hg.1 | 9.81 | 10.05 | 1.18 | 0.009 | PI4KA |
| TC1000009589.hg.1 | 7.88 | 8.11 | 1.18 | 0.0092 | PITRM1 |
| TC2000008473.hg.1 | 6.51 | 6.76 | 1.18 | 0.0097 | KIF16B |
| TC0300011167.hg.1 | 6.35 | 6.59 | 1.18 | 0.0098 | BAP1 |
| TC0100009555.hg.1 | 14.21 | 14.45 | 1.18 | 0.0099 | CD2 |
| TC1900011677.hg.1 | 10.22 | 10.47 | 1.19 | 4.74E-06 | IL27RA |
| TC0700010941.hg.1 | 11.66 | 11.91 | 1.19 | 5.30E-06 | MYO1G |
| TC1200010633.hg.1 | 9.43 | 9.68 | 1.19 | 6.10E-06 | MCRS1 |
| TC0700007949.hg.1 | 10.85 | 11.11 | 1.19 | 7.70E-06 | POM121 |
| TC1100009666.hg.1 | 9.6 | 9.85 | 1.19 | 1.79E-05 | SIGIRR |
| TC0100006675.hg.1 | 11.93 | 12.18 | 1.19 | 1.96E-05 | KCNAB2 |
| TC1700007772.hg.1 | 9.62 | 9.86 | 1.19 | 2.24E-05 | PSMD3 |
| TC1700007740.hg.1 | 13.84 | 14.08 | 1.19 | 4.02E-05 | RPL19 |
| TC0200016360.hg.1 | 8.9 | 9.15 | 1.19 | 4.32E-05 | STK25 |
| TC1900007869.hg.1 | 9.61 | 9.87 | 1.19 | 4.43E-05 | RBM42 |
| TC1900009198.hg.1 | 8.71 | 8.95 | 1.19 | 6.45E-05 | BTBD2 |
| TC0X00008388.hg.1 | 9.75 | 10.01 | 1.19 | 6.72E-05 | SASH3 |
| TC0600010343.hg.1 | 9.66 | 9.92 | 1.19 | 7.49E-05 | TBP |
| TC1600008144.hg.1 | 9.55 | 9.81 | 1.19 | 8.07E-05 | C16orf70 |
| TC1600011374.hg.1 | 12.27 | 12.53 | 1.19 | 8.32E-05 | SPN |
| TC1200007866.hg.1 | 11.5 | 11.75 | 1.19 | 8.32E-05 | SHMT2 |
| TC0500012485.hg.1 | 11.72 | 11.96 | 1.19 | 0.0001 | RBM22 |
| TC1900009133.hg.1 | 10.33 | 10.59 | 1.19 | 0.0001 | POLR2E |
| TC1600010734.hg.1 | 8.98 | 9.23 | 1.19 | 0.0002 | NOB1 |
| TC1600007504.hg.1 | 9.74 | 9.99 | 1.19 | 0.0002 | SETD1A |
| TC0600008972.hg.1 | 13.4 | 13.65 | 1.19 | 0.0002 | PRDM1 |
| TC1600009944.hg.1 | 8.3 | 8.56 | 1.19 | 0.0002 | KCTD13 |
| TC2000008678.hg.1 | 14.1 | 14.35 | 1.19 | 0.0002 | CST3 |
| TC1100013047.hg.1 | 7.63 | 7.88 | 1.19 | 0.0003 | SF3B2 |
| TC1900010675.hg.1 | 12.02 | 12.27 | 1.19 | 0.0003 | FBL |
| TC1200008213.hg.1 | 8.09 | 8.34 | 1.19 | 0.0003 | ATXN7L3B |
| TC0200013894.hg.1 | 8.32 | 8.57 | 1.19 | 0.0003 | ANAPC1 |
| TSUnmapped00000427.hg.1 | 11.13 | 11.39 | 1.19 | 0.0003 | FBL |
| TC1100013129.hg.1 | 13.88 | 14.13 | 1.19 | 0.0003 | CTSD |
| TC0700010453.hg.1 | 12.54 | 12.79 | 1.19 | 0.0006 | TOMM7 |
| TC0500008855.hg.1 | 11.5 | 11.75 | 1.19 | 0.0006 | SLC35A4 |
| TC1700008995.hg.1 | 9.88 | 10.13 | 1.19 | 0.0006 | 09-ספט |
| TC1600008657.hg.1 | 10.56 | 10.81 | 1.19 | 0.0007 | USP10 |
| TC1700010202.hg.1 | 10 | 10.25 | 1.19 | 0.0008 | KIAA0100 |
| TC1100012478.hg.1 | 11.3 | 11.56 | 1.19 | 0.0008 | CD3D |
| TC0600014273.hg.1 | 15.06 | 15.31 | 1.19 | 0.0009 | HLA-DRB1 |
| TC1900007386.hg.1 | 7.6 | 7.85 | 1.19 | 0.0009 | SSBP4 |
| TC1100013050.hg.1 | 9.36 | 9.6 | 1.19 | 0.0009 | RBM14 |
| TC2000010001.hg.1 | 7.3 | 7.55 | 1.19 | 0.001 | PIGU |
| TC1100013054.hg.1 | 8.32 | 8.58 | 1.19 | 0.0011 | RAD9A |
| TC0X00008080.hg.1 | 11.05 | 11.3 | 1.19 | 0.0013 | VSIG1 |
| TC1200007647.hg.1 | 7.33 | 7.58 | 1.19 | 0.0014 | ACVR1B |
| TC1900011414.hg.1 | 10.13 | 10.39 | 1.19 | 0.0014 | LAIR1 |
| TC0200008458.hg.1 | 5.35 | 5.6 | 1.19 | 0.0015 | ZNF2 |
| TC1700011699.hg.1 | 8.79 | 9.04 | 1.19 | 0.0015 | CD300C |
| TC1900010863.hg.1 | 6.7 | 6.95 | 1.19 | 0.0015 | SMG9 |
| TC1700009612.hg.1 | 6.47 | 6.72 | 1.19 | 0.0016 | CLEC10A |
| TC1700008194.hg.1 | 6.47 | 6.72 | 1.19 | 0.0016 | ABI3 |
| TC0900011659.hg.1 | 5.66 | 5.92 | 1.19 | 0.0017 | C9orf114 |
| TC1100011097.hg.1 | 6.77 | 7.02 | 1.19 | 0.0017 | ZBTB3 |
| TC0600014238.hg.1 | 6.67 | 6.92 | 1.19 | 0.0017 | BLOC1S5 |
| TC0400011548.hg.1 | 13.42 | 13.68 | 1.19 | 0.0019 | LEF1 |
| TC1600009102.hg.1 | 10.66 | 10.91 | 1.19 | 0.0019 | RNPS1 |
| TC1100011375.hg.1 | 8.13 | 8.38 | 1.19 | 0.002 | C11orf24 |
| TC1100009040.hg.1 | 9.97 | 10.22 | 1.19 | 0.002 | C11orf1 |
| TC1900009208.hg.1 | 7.31 | 7.56 | 1.19 | 0.0021 | AP3D1 |
| TC1400009697.hg.1 | 10.86 | 11.11 | 1.19 | 0.0022 | NPC2; MIR4709 |
| TC1100011514.hg.1 | 6.43 | 6.69 | 1.19 | 0.0022 | DHCR7 |
| TC1000007461.hg.1 | 8.36 | 8.61 | 1.19 | 0.0025 | RASSF4 |
| TC0600009322.hg.1 | 8.19 | 8.44 | 1.19 | 0.0025 | HSF2 |
| TC0700007859.hg.1 | 6.23 | 6.49 | 1.19 | 0.0028 | TYW1 |
| TC0900012124.hg.1 | 7.64 | 7.88 | 1.19 | 0.0029 | POLR1E |
| TC0300009944.hg.1 | 5.06 | 5.32 | 1.19 | 0.0029 | FAM43A |
| TC1100006576.hg.1 | 11.29 | 11.54 | 1.19 | 0.0031 | CD81 |
| TC2000007246.hg.1 | 6.43 | 6.68 | 1.19 | 0.0036 | DLGAP4 |
| TC0700006634.hg.1 | 6.66 | 6.91 | 1.19 | 0.0042 | AIMP2 |
| TC1100007787.hg.1 | 12.72 | 12.97 | 1.19 | 0.0045 | CD6 |
| TC1100013041.hg.1 | 7.44 | 7.7 | 1.19 | 0.0052 | VPS51 |
| TC0100012020.hg.1 | 6.72 | 6.97 | 1.19 | 0.0053 | COA6 |
| TC0600007196.hg.1 | 5.79 | 6.04 | 1.19 | 0.0055 | MRS2 |
| TC0700013426.hg.1 | 9.85 | 10.1 | 1.19 | 0.0061 | PVRIG |
| TC0600009772.hg.1 | 5.44 | 5.69 | 1.19 | 0.0068 | SASH1 |
| TC0200010713.hg.1 | 7.9 | 8.15 | 1.19 | 0.0069 | ATIC |
| TC0X00007039.hg.1 | 6.92 | 7.17 | 1.19 | 0.0075 | GPR34 |
| TC0500008632.hg.1 | 5.43 | 5.68 | 1.19 | 0.0078 | SLC22A5 |
| TC0400012989.hg.1 | 6.97 | 7.22 | 1.19 | 0.0078 | TMEM192 |
| TC0100016024.hg.1 | 9.69 | 9.94 | 1.19 | 0.0079 | SH2D2A |
| TC1200010556.hg.1 | 7.16 | 7.4 | 1.19 | 0.0089 | HDAC7 |
| TC0600010008.hg.1 | 7.18 | 7.43 | 1.19 | 0.0093 | GTF2H5 |
| TC1100013037.hg.1 | 9.74 | 10 | 1.2 | 2.31E-06 | ARL2 |
| TC1100006771.hg.1 | 12.58 | 12.84 | 1.2 | 3.96E-06 | EIF3F |
| TC1100008025.hg.1 | 7.84 | 8.1 | 1.2 | 7.82E-06 | SCYL1 |
| TC0700009067.hg.1 | 12.37 | 12.63 | 1.2 | 9.14E-06 | ATP6V1F |
| TC2200008641.hg.1 | 15.24 | 15.51 | 1.2 | 1.01E-05 | RAC2 |
| TC0300013344.hg.1 | 8.61 | 8.87 | 1.2 | 1.44E-05 | KLHL6 |
| TC0X00008908.hg.1 | 14.83 | 15.09 | 1.2 | 1.79E-05 | SLC25A6 |
| TSUnmapped00000397.hg.1 | 9.79 | 10.06 | 1.2 | 2.90E-05 | EIF3F |
| TC0400012933.hg.1 | 10.4 | 10.66 | 1.2 | 3.08E-05 | NAAA |
| TC1200012646.hg.1 | 11.1 | 11.37 | 1.2 | 3.42E-05 | ESYT1 |
| TC0100018510.hg.1 | 8.4 | 8.67 | 1.2 | 5.02E-05 | GLMP |
| TC1600011367.hg.1 | 9.61 | 9.87 | 1.2 | 5.03E-05 | SPNS1 |
| TC2200009262.hg.1 | 12.6 | 12.86 | 1.2 | 5.74E-05 | EIF3L |
| TC1900007181.hg.1 | 11.79 | 12.06 | 1.2 | 6.19E-05 | TECR; MIR639 |
| TC1100007785.hg.1 | 12.48 | 12.75 | 1.2 | 6.78E-05 | TMEM109 |
| TC1200008677.hg.1 | 14.57 | 14.84 | 1.2 | 6.83E-05 | RPL18A |
| TC1900008013.hg.1 | 10.59 | 10.86 | 1.2 | 7.12E-05 | PSMD8 |
| TC0X00007209.hg.1 | 9.32 | 9.58 | 1.2 | 8.07E-05 | PQBP1 |
| TC0700008081.hg.1 | 11.52 | 11.78 | 1.2 | 8.62E-05 | MDH2 |
| TC0100009417.hg.1 | 13.22 | 13.48 | 1.2 | 8.63E-05 | C1orf162 |
| TSUnmapped00000677.hg.1 | 13.01 | 13.28 | 1.2 | 8.76E-05 | RPL7A |
| TC1500010890.hg.1 | 9.25 | 9.51 | 1.2 | 0.0002 | HEXA |
| TC0300009167.hg.1 | 8.41 | 8.67 | 1.2 | 0.0003 | EIF2A |
| TC1600011378.hg.1 | 10.15 | 10.42 | 1.2 | 0.0003 | MVP; PAGR1 |
| TC0X00008495.hg.1 | 9.53 | 9.8 | 1.2 | 0.0003 | FAM127A |
| TC0500013106.hg.1 | 8.11 | 8.37 | 1.2 | 0.0003 | MGAT1 |
| TC0300007386.hg.1 | 8.99 | 9.25 | 1.2 | 0.0003 | APEH |
| TC1900011857.hg.1 | 8.02 | 8.28 | 1.2 | 0.0003 | NDUFA7 |
| TC1200012859.hg.1 | 10.75 | 11.01 | 1.2 | 0.0004 | RHOF |
| TC0100017500.hg.1 | 10.22 | 10.48 | 1.2 | 0.0005 | TMEM63A |
| TC1100011222.hg.1 | 7.87 | 8.14 | 1.2 | 0.0006 | SLC25A45 |
| TC2200007361.hg.1 | 8.88 | 9.14 | 1.2 | 0.0006 | TOMM22 |
| TC1700009637.hg.1 | 10.06 | 10.32 | 1.2 | 0.0006 | PLSCR3; TMEM256; TMEM256-PLSCR3 |
| TC0200008063.hg.1 | 8.62 | 8.88 | 1.2 | 0.0007 | DGUOK |
| TC1200007899.hg.1 | 6.95 | 7.22 | 1.2 | 0.0007 | 09-מרץ |
| TC0800007127.hg.1 | 9.76 | 10.01 | 1.2 | 0.0008 | ELP3 |
| TC1300008181.hg.1 | 9.75 | 10.01 | 1.2 | 0.0008 | CHAMP1 |
| TC2200007547.hg.1 | 8.72 | 8.98 | 1.2 | 0.0008 | TSPO |
| TC0600011859.hg.1 | 6.82 | 7.08 | 1.2 | 0.001 | MRPL2 |
| TC1800008680.hg.1 | 8.04 | 8.3 | 1.2 | 0.001 | CXXC1 |
| TC0100010347.hg.1 | 8.98 | 9.25 | 1.2 | 0.001 | SDHC |
| TC0400012245.hg.1 | 10.85 | 11.11 | 1.2 | 0.0011 | FAM198B |
| TC0300008715.hg.1 | 7.39 | 7.65 | 1.2 | 0.0013 | EEFSEC |
| TC0900009912.hg.1 | 9.4 | 9.65 | 1.2 | 0.0013 | SIGMAR1 |
| TC0700012684.hg.1 | 8.96 | 9.22 | 1.2 | 0.0014 | AKR1B1 |
| TC2000008379.hg.1 | 6.39 | 6.65 | 1.2 | 0.0015 | MKKS |
| TC1100013051.hg.1 | 9.08 | 9.35 | 1.2 | 0.0016 | RBM14-RBM4 |
| TC0100013408.hg.1 | 7.21 | 7.48 | 1.2 | 0.0016 | GPN2 |
| TC0800007013.hg.1 | 9.12 | 9.38 | 1.2 | 0.0022 | CHMP7 |
| TC1900007340.hg.1 | 10.16 | 10.43 | 1.2 | 0.0025 | MAP1S |
| TC1900010792.hg.1 | 8.58 | 8.84 | 1.2 | 0.0026 | DEDD2 |
| TC0200008452.hg.1 | 10.84 | 11.11 | 1.2 | 0.0031 | MAL |
| TC0300006961.hg.1 | 4.92 | 5.19 | 1.2 | 0.0033 | CMTM8 |
| TC0500012781.hg.1 | 10.78 | 11.04 | 1.2 | 0.0034 | DOCK2 |
| TC1400010763.hg.1 | 7.11 | 7.38 | 1.2 | 0.0034 | TMEM229B |
| TC1900009240.hg.1 | 7.79 | 8.05 | 1.2 | 0.0035 | GNG7 |
| TC0X00008392.hg.1 | 7.35 | 7.61 | 1.2 | 0.0041 | UTP14A |
| TC1200010407.hg.1 | 7.28 | 7.54 | 1.2 | 0.0051 | KIF21A |
| TC1900009310.hg.1 | 7 | 7.26 | 1.2 | 0.0054 | MATK |
| TC1100009171.hg.1 | 6.5 | 6.77 | 1.2 | 0.0059 | RNF214 |
| TC1200012723.hg.1 | 6.54 | 6.8 | 1.2 | 0.0062 | ZNF664 |
| TC1000007990.hg.1 | 7.55 | 7.82 | 1.2 | 0.007 | DDIT4 |
| TC0X00011232.hg.1 | 4.7 | 4.96 | 1.2 | 0.0071 | CMC4; MTCP1 |
| TC0800012279.hg.1 | 4.98 | 5.24 | 1.2 | 0.0079 | SORBS3 |
| TC0100015079.hg.1 | 4.04 | 4.3 | 1.2 | 0.0079 | EXTL2 |
| TC1900010597.hg.1 | 10.38 | 10.64 | 1.21 | 2.17E-06 | YIF1B |
| TC0100017793.hg.1 | 8.74 | 9.02 | 1.21 | 5.82E-06 | IRF2BP2 |
| TC0700006795.hg.1 | 13.13 | 13.41 | 1.21 | 7.70E-06 | AHR |
| TC0500009614.hg.1 | 14.19 | 14.46 | 1.21 | 1.20E-05 | PRELID1 |
| TC0200012073.hg.1 | 9.45 | 9.72 | 1.21 | 1.25E-05 | GTF3C2 |
| TC0900008902.hg.1 | 8.53 | 8.8 | 1.21 | 3.41E-05 | PPP2R4 |
| TC0300013920.hg.1 | 7.6 | 7.88 | 1.21 | 4.33E-05 | NRROS |
| TC1600010028.hg.1 | 8.86 | 9.13 | 1.21 | 6.95E-05 | C16orf58 |
| TC1900011744.hg.1 | 8.99 | 9.26 | 1.21 | 7.37E-05 | BCKDHA |
| TC1100013052.hg.1 | 10.69 | 10.96 | 1.21 | 7.38E-05 | RBM4 |
| TC1200006701.hg.1 | 8.83 | 9.1 | 1.21 | 8.07E-05 | NECAP1 |
| TC1700006808.hg.1 | 7.26 | 7.54 | 1.21 | 8.07E-05 | RANGRF |
| TC1900012007.hg.1 | 9.38 | 9.65 | 1.21 | 8.52E-05 | NUP62; IL4I1 |
| TC1900011781.hg.1 | 9.58 | 9.86 | 1.21 | 0.0001 | PRMT1 |
| TC0600007800.hg.1 | 12.79 | 13.07 | 1.21 | 0.0002 | RPL10A |
| TC1600006886.hg.1 | 9.13 | 9.41 | 1.21 | 0.0002 | NUBP1 |
| TC2200008475.hg.1 | 8.63 | 8.91 | 1.21 | 0.0002 | PIK3IP1 |
| TC0100018200.hg.1 | 7.58 | 7.85 | 1.21 | 0.0003 | SEPN1 |
| TC0300010329.hg.1 | 9.86 | 10.14 | 1.21 | 0.0004 | NUP210 |
| TC0900012021.hg.1 | 9.13 | 9.4 | 1.21 | 0.0004 | FBXW5 |
| TC1900011729.hg.1 | 9.13 | 9.41 | 1.21 | 0.0004 | TIMM50 |
| TC1400010704.hg.1 | 9.71 | 9.99 | 1.21 | 0.0005 | CRIP1 |
| TC0100007690.hg.1 | 9.26 | 9.54 | 1.21 | 0.0006 | RBBP4 |
| TC1100006791.hg.1 | 11.91 | 12.19 | 1.21 | 0.0006 | RPL27A; SNORA3A; SNORA3B |
| TC1200010850.hg.1 | 12.39 | 12.67 | 1.21 | 0.0007 | TESPA1 |
| TC0100016337.hg.1 | 8.17 | 8.44 | 1.21 | 0.0007 | XCL2 |
| TC0700013491.hg.1 | 12.41 | 12.69 | 1.21 | 0.0008 | GIMAP1-GIMAP5; GIMAP5; GIMAP1 |
| TC0100016021.hg.1 | 7.38 | 7.65 | 1.21 | 0.0008 | MRPL24 |
| TC0X00010793.hg.1 | 6.59 | 6.87 | 1.21 | 0.0009 | ZDHHC9 |
| TC0600014111.hg.1 | 8.26 | 8.53 | 1.21 | 0.0009 | SYNGAP1; MIR5004 |
| TC2200007467.hg.1 | 7.92 | 8.19 | 1.21 | 0.001 | ZC3H7B |
| TC0100009441.hg.1 | 5.04 | 5.32 | 1.21 | 0.001 | CTTNBP2NL |
| TC0300013835.hg.1 | 6.64 | 6.91 | 1.21 | 0.0012 | TEX264 |
| TC2200009152.hg.1 | 7.74 | 8.01 | 1.21 | 0.0014 | PLXNB2 |
| TC0200008501.hg.1 | 8.2 | 8.48 | 1.21 | 0.0027 | ITPRIPL1 |
| TC0300014094.hg.1 | 4.79 | 5.07 | 1.21 | 0.0029 | TCTEX1D2 |
| TC1600011115.hg.1 | 4.48 | 4.76 | 1.21 | 0.0031 | EMC8 |
| TC1600010450.hg.1 | 5.97 | 6.24 | 1.21 | 0.0042 | CIAPIN1 |
| TC1200010490.hg.1 | 9.76 | 10.04 | 1.21 | 0.005 | NELL2 |
| TC0100016290.hg.1 | 6.76 | 7.04 | 1.21 | 0.0072 | TADA1 |
| TC1900006488.hg.1 | 12.87 | 13.16 | 1.22 | 3.64E-07 | PTBP1; MIR4745 |
| TC0500012455.hg.1 | 11.18 | 11.46 | 1.22 | 9.09E-07 | CSF1R |
| TC0Y00006882.hg.1 | 14.81 | 15.1 | 1.22 | 5.58E-06 | SLC25A6 |
| TC1600010711.hg.1 | 10.48 | 10.76 | 1.22 | 6.57E-06 | CHTF8 |
| TC0300007470.hg.1 | 8.51 | 8.8 | 1.22 | 2.05E-05 | PPM1M |
| TC0800009819.hg.1 | 12.58 | 12.87 | 1.22 | 2.14E-05 | DOK2 |
| TC0700013043.hg.1 | 10.4 | 10.69 | 1.22 | 3.11E-05 | GIMAP6 |
| TC0600013433.hg.1 | 7.17 | 7.46 | 1.22 | 3.37E-05 | SF3B5 |
| TC1900007320.hg.1 | 10.64 | 10.93 | 1.22 | 4.26E-05 | DDA1 |
| TC1100007790.hg.1 | 11.38 | 11.67 | 1.22 | 4.32E-05 | CD5 |
| TC1600009935.hg.1 | 11.87 | 12.16 | 1.22 | 5.04E-05 | SPN |
| TC0X00011163.hg.1 | 7.59 | 7.88 | 1.22 | 7.73E-05 | FAM58A |
| TC0100013761.hg.1 | 10.49 | 10.78 | 1.22 | 8.52E-05 | FHL3 |
| TC1900009045.hg.1 | 11.72 | 12 | 1.22 | 8.71E-05 | RPS5 |
| TC1900008924.hg.1 | 9.7 | 9.99 | 1.22 | 9.54E-05 | U2AF2 |
| TC0200014512.hg.1 | 10.05 | 10.33 | 1.22 | 0.0001 | ZEB2 |
| TC2200008477.hg.1 | 8.54 | 8.83 | 1.22 | 0.0002 | PATZ1 |
| TC1700009277.hg.1 | 8.09 | 8.38 | 1.22 | 0.0002 | TBCD |
| TC0100007660.hg.1 | 7.33 | 7.62 | 1.22 | 0.0003 | TMEM39B |
| TC1900008164.hg.1 | 14.46 | 14.75 | 1.22 | 0.0006 | RPS19 |
| TC1900010612.hg.1 | 9.07 | 9.35 | 1.22 | 0.0006 | MAP4K1 |
| TC1100007941.hg.1 | 7.89 | 8.17 | 1.22 | 0.0012 | FKBP2 |
| TC1400009732.hg.1 | 11.07 | 11.36 | 1.22 | 0.0017 | C14orf1 |
| TC1200008933.hg.1 | 8.4 | 8.68 | 1.22 | 0.0021 | TPCN1 |
| TC1900011469.hg.1 | 6.98 | 7.26 | 1.22 | 0.0025 | TMEM238 |
| TC0300013751.hg.1 | 5 | 5.29 | 1.22 | 0.0026 | BDH1 |
| TC1600011513.hg.1 | 5.86 | 6.14 | 1.22 | 0.0032 | BOLA2; BOLA2B |
| TC0100008752.hg.1 | 7.8 | 8.09 | 1.22 | 0.0052 | TYW3 |
| TC1400007039.hg.1 | 4.47 | 4.75 | 1.22 | 0.0078 | FANCM |
| TC2000007512.hg.1 | 10.63 | 10.93 | 1.23 | 9.23E-06 | PCIF1 |
| TC1400006656.hg.1 | 13.26 | 13.56 | 1.23 | 1.44E-05 | OXA1L |
| TC1600007348.hg.1 | 9.05 | 9.35 | 1.23 | 1.93E-05 | SGF29 |
| TC1600010772.hg.1 | 8.35 | 8.65 | 1.23 | 2.24E-05 | VAC14 |
| TC1000011718.hg.1 | 11.01 | 11.31 | 1.23 | 2.35E-05 | CALHM2 |
| TC1100011083.hg.1 | 10.76 | 11.07 | 1.23 | 3.11E-05 | AHNAK |
| TC0800012444.hg.1 | 15.23 | 15.52 | 1.23 | 5.48E-05 | PABPC1; MIR7705 |
| TC1200012583.hg.1 | 12.08 | 12.38 | 1.23 | 6.15E-05 | CD27 |
| TC1100008175.hg.1 | 8.25 | 8.55 | 1.23 | 0.0002 | NDUFS8; MIR4691; MIR7113 |
| TC1100009707.hg.1 | 9.16 | 9.46 | 1.23 | 0.0003 | POLR2L |
| TC1500007645.hg.1 | 6.17 | 6.47 | 1.23 | 0.0003 | MAP2K5 |
| TC1600006888.hg.1 | 10.37 | 10.68 | 1.23 | 0.0004 | CIITA |
| TC1700006646.hg.1 | 7.37 | 7.66 | 1.23 | 0.0007 | MINK1 |
| TC1500010887.hg.1 | 7.69 | 7.99 | 1.23 | 0.0008 | CLN6 |
| TC0100013796.hg.1 | 9.98 | 10.28 | 1.23 | 0.0009 | PABPC4 |
| TC1100006547.hg.1 | 5.48 | 5.78 | 1.23 | 0.0011 | MRPL23 |
| TC0300013834.hg.1 | 6.91 | 7.21 | 1.23 | 0.0013 | RAD54L2 |
| TC0X00010670.hg.1 | 5.33 | 5.63 | 1.23 | 0.0015 | TMEM255A |
| TC2000007760.hg.1 | 6.73 | 7.04 | 1.23 | 0.0016 | TSHZ2 |
| TC0900009860.hg.1 | 7.17 | 7.48 | 1.23 | 0.0019 | AQP3 |
| TC1900011775.hg.1 | 8.77 | 9.06 | 1.23 | 0.0019 | GRWD1 |
| TC0600007657.hg.1 | 12.55 | 12.85 | 1.23 | 0.0027 | HLA-DQA1 |
| TC0500009422.hg.1 | 12.04 | 12.34 | 1.23 | 0.0031 | NPM1 |
| TC2200009277.hg.1 | 5.7 | 5.99 | 1.23 | 0.0038 | XPNPEP3 |
| TC1500010720.hg.1 | 6.51 | 6.8 | 1.23 | 0.0055 | IVD |
| TC2000008001.hg.1 | 8.09 | 8.4 | 1.24 | 3.73E-06 | ADRM1 |
| TC1900008103.hg.1 | 10.58 | 10.89 | 1.24 | 4.46E-06 | PLD3 |
| TC0100018246.hg.1 | 11.61 | 11.93 | 1.24 | 1.08E-05 | LRRC8C |
| TC0100007672.hg.1 | 8.71 | 9.03 | 1.24 | 1.63E-05 | EIF3I |
| TC1900008689.hg.1 | 9.53 | 9.83 | 1.24 | 2.84E-05 | PPP2R1A |
| TC1000007954.hg.1 | 7.66 | 7.97 | 1.24 | 4.52E-05 | SLC29A3 |
| TC1400009690.hg.1 | 6.19 | 6.5 | 1.24 | 6.83E-05 | ABCD4 |
| TC1200008900.hg.1 | 7.88 | 8.19 | 1.24 | 6.83E-05 | ERP29 |
| TC0200010567.hg.1 | 12.19 | 12.5 | 1.24 | 0.0001 | EEF1B2; SNORA41 |
| TC0500008684.hg.1 | 11.22 | 11.53 | 1.24 | 0.0001 | TCF7 |
| TC1000009090.hg.1 | 7.68 | 7.99 | 1.24 | 0.0001 | BAG3 |
| TC1700006946.hg.1 | 8.94 | 9.26 | 1.24 | 0.0001 | HS3ST3B1 |
| TC1300008660.hg.1 | 9.15 | 9.46 | 1.24 | 0.0002 | PROSER1 |
| TC2000009198.hg.1 | 5.05 | 5.35 | 1.24 | 0.0002 | ADA |
| TC0900010969.hg.1 | 6.28 | 6.59 | 1.24 | 0.0002 | CORO2A |
| TC1900008113.hg.1 | 6.69 | 7 | 1.24 | 0.0004 | LTBP4 |
| TC0300013827.hg.1 | 5.89 | 6.21 | 1.24 | 0.0006 | WDR6 |
| TC1000009691.hg.1 | 8.32 | 8.63 | 1.24 | 0.0007 | IL2RA |
| TC1200006656.hg.1 | 9.99 | 10.3 | 1.24 | 0.0008 | C12orf57; RNU7-1 |
| TC1600011520.hg.1 | 11.9 | 12.21 | 1.24 | 0.0011 | 01-ספט |
| TC1100009450.hg.1 | 8.31 | 8.63 | 1.24 | 0.0018 | DCPS |
| TC1700012226.hg.1 | 7.2 | 7.51 | 1.24 | 0.0033 | ADAP2 |
| TC0X00006625.hg.1 | 9.76 | 10.07 | 1.24 | 0.0037 | TLR7 |
| TC1600006550.hg.1 | 6.84 | 7.15 | 1.24 | 0.0073 | TMEM204 |
| TC1200007536.hg.1 | 6.41 | 6.73 | 1.24 | 0.0095 | CCDC65 |
| TC1100008144.hg.1 | 12.49 | 12.81 | 1.25 | 2.24E-07 | GSTP1 |
| TC0700008072.hg.1 | 8.82 | 9.14 | 1.25 | 1.08E-05 | RHBDD2 |
| TC0100009344.hg.1 | 8.56 | 8.88 | 1.25 | 5.47E-05 | SARS |
| TC1900011851.hg.1 | 9.16 | 9.48 | 1.25 | 6.63E-05 | NDUFA11 |
| TC1900008141.hg.1 | 9.05 | 9.38 | 1.25 | 6.92E-05 | CYP2S1 |
| TC0700008552.hg.1 | 7.36 | 7.68 | 1.25 | 8.82E-05 | AGFG2 |
| TC0100010252.hg.1 | 10.01 | 10.33 | 1.25 | 8.91E-05 | FCER1A |
| TC1000011679.hg.1 | 6.23 | 6.56 | 1.25 | 9.43E-05 | CUEDC2 |
| TC1100008049.hg.1 | 12.63 | 12.96 | 1.25 | 0.0002 | BANF1 |
| TC0300008713.hg.1 | 8.49 | 8.81 | 1.25 | 0.0002 | SEC61A1 |
| TC2100006982.hg.1 | 8.91 | 9.23 | 1.25 | 0.0004 | LINC00649 |
| TC1700010859.hg.1 | 10.39 | 10.72 | 1.25 | 0.0004 | DCAKD |
| TC2200007204.hg.1 | 6.9 | 7.22 | 1.25 | 0.0005 | HMOX1 |
| TC0700010925.hg.1 | 7.47 | 7.79 | 1.25 | 0.0005 | DDX56 |
| TC0600014095.hg.1 | 7.3 | 7.62 | 1.25 | 0.0006 | RPP21 |
| TC0800008845.hg.1 | 10.65 | 10.97 | 1.25 | 0.0006 | MYC |
| TC2200008637.hg.1 | 11.48 | 11.8 | 1.25 | 0.0008 | IL2RB |
| TC1000011427.hg.1 | 7.26 | 7.58 | 1.25 | 0.0013 | IDE |
| TC0200015779.hg.1 | 7.26 | 7.58 | 1.25 | 0.002 | DNPEP |
| TC0300012397.hg.1 | 6.93 | 7.25 | 1.25 | 0.0034 | PLXND1 |
| TC0100017844.hg.1 | 7.52 | 7.85 | 1.26 | 1.52E-05 | NID1 |
| TC1900007319.hg.1 | 9.15 | 9.48 | 1.26 | 1.52E-05 | MRPL34 |
| TC1900007329.hg.1 | 7.82 | 8.15 | 1.26 | 2.45E-05 | PGLS |
| TC1200007861.hg.1 | 11.24 | 11.57 | 1.26 | 2.90E-05 | LRP1 |
| TC1600008709.hg.1 | 8.47 | 8.8 | 1.26 | 4.02E-05 | COX4I1 |
| TC0800009511.hg.1 | 5.73 | 6.06 | 1.26 | 0.0003 | SGK223 |
| TC0300011259.hg.1 | 9.32 | 9.65 | 1.26 | 0.0008 | ARHGEF3 |
| TC0900010971.hg.1 | 6.94 | 7.27 | 1.26 | 0.0011 | TBC1D2 |
| TC2000009267.hg.1 | 10.48 | 10.82 | 1.27 | 3.46E-08 | SLC35C2 |
| TC2000008230.hg.1 | 8.56 | 8.91 | 1.27 | 2.55E-07 | C20orf27 |
| TC0900007085.hg.1 | 11.29 | 11.64 | 1.27 | 3.64E-07 | TESK1; MIR4667 |
| TC1900008633.hg.1 | 7.86 | 8.2 | 1.27 | 4.00E-06 | CD33 |
| TC0300007432.hg.1 | 10.85 | 11.19 | 1.27 | 4.52E-05 | MAPKAPK3 |
| TC1900008433.hg.1 | 10.48 | 10.82 | 1.27 | 7.79E-05 | GLTSCR2; SNORD23 |
| TC1100010730.hg.1 | 7.23 | 7.57 | 1.27 | 0.0006 | ACP2 |
| TC1900009633.hg.1 | 8.87 | 9.21 | 1.27 | 0.0012 | S1PR5 |
| TC1700007620.hg.1 | 7.25 | 7.6 | 1.27 | 0.0015 | CCL4L2; CCL4; CCL4L1 |
| TC1900011929.hg.1 | 6.85 | 7.19 | 1.27 | 0.003 | IGFLR1 |
| TC0200009299.hg.1 | 7.31 | 7.65 | 1.27 | 0.0034 | IMP4 |
| TC0100007370.hg.1 | 8.93 | 9.28 | 1.27 | 0.0046 | RCAN3 |
| TC0700013591.hg.1 | 12.34 | 12.69 | 1.28 | 7.55E-07 | ATP5J2 |
| TC1900011733.hg.1 | 9.86 | 10.22 | 1.28 | 1.06E-06 | RAB4B; MIA-RAB4B; RAB4B-EGLN2 |
| TC0600014277.hg.1 | 13.19 | 13.54 | 1.28 | 2.12E-06 | HLA-DMA |
| TC1900009608.hg.1 | 11.9 | 12.25 | 1.28 | 2.84E-05 | EIF3G |
| TC0500012147.hg.1 | 10.23 | 10.58 | 1.28 | 7.77E-05 | HNRNPA0 |
| TC1700012185.hg.1 | 7.5 | 7.87 | 1.28 | 0.0002 | TNFSF12 |
| TC0500011095.hg.1 | 9.29 | 9.65 | 1.28 | 0.0003 | MRPS27 |
| TC0900012033.hg.1 | 7.64 | 8 | 1.28 | 0.0003 | DPP7 |
| TC0200010523.hg.1 | 10.7 | 11.05 | 1.28 | 0.0003 | CD28 |
| TC1500010894.hg.1 | 8.01 | 8.36 | 1.28 | 0.0004 | SNUPN |
| TC0600007596.hg.1 | 5.94 | 6.29 | 1.28 | 0.0006 | TNF |
| TC0300011050.hg.1 | 10.27 | 10.64 | 1.29 | 2.84E-07 | QARS; MIR6890 |
| TC1200010838.hg.1 | 10.77 | 11.13 | 1.29 | 3.64E-07 | ZNF385A |
| TC1200007461.hg.1 | 12.25 | 12.62 | 1.29 | 1.01E-05 | PCED1B |
| TC0200008556.hg.1 | 9.4 | 9.78 | 1.29 | 5.17E-05 | ZAP70 |
| TC0900009946.hg.1 | 8 | 8.37 | 1.29 | 0.0002 | SIT1 |
| TC0900009861.hg.1 | 6.87 | 7.24 | 1.29 | 0.0009 | NOL6 |
| TC0100013339.hg.1 | 12.57 | 12.93 | 1.29 | 0.0019 | RUNX3 |
| TC0200011362.hg.1 | 9.54 | 9.92 | 1.3 | 1.05E-05 | RNPEPL1 |
| TC1900011251.hg.1 | 13.85 | 14.24 | 1.31 | 2.58E-06 | NKG7 |
| TC1900009192.hg.1 | 8.03 | 8.42 | 1.31 | 3.82E-06 | ABHD17A |
| TC1900009628.hg.1 | 9.43 | 9.82 | 1.31 | 1.63E-05 | CDC37; MIR1181 |
| TC0600011499.hg.1 | 10.46 | 10.85 | 1.31 | 0.0011 | HLA-DQB1 |
| TC0900011769.hg.1 | 8.32 | 8.7 | 1.31 | 0.0012 | FAM78A |
| TC0500009587.hg.1 | 11.23 | 11.63 | 1.32 | 9.88E-08 | HIGD2A |
| TC1100013193.hg.1 | 10.42 | 10.82 | 1.32 | 7.55E-07 | PTPRCAP |
| TC0100012869.hg.1 | 9.85 | 10.25 | 1.32 | 2.84E-05 | SRM |
| TC1600008979.hg.1 | 5.98 | 6.4 | 1.33 | 2.58E-06 | NARFL |
| TC1900006520.hg.1 | 10.46 | 10.87 | 1.33 | 3.73E-06 | ATP5D |
| TC0100007676.hg.1 | 12.08 | 12.49 | 1.33 | 4.32E-05 | LCK |
| TC2000009971.hg.1 | 10.12 | 10.53 | 1.33 | 0.0002 | SIRPG |
| TC2200007312.hg.1 | 12.56 | 12.98 | 1.34 | 2.60E-07 | LGALS1 |
| TC1200012571.hg.1 | 9.92 | 10.34 | 1.34 | 1.07E-06 | ITFG2 |
| TC1900011779.hg.1 | 11.94 | 12.37 | 1.35 | 9.85E-07 | FLT3LG |
| TC0800007004.hg.1 | 7.19 | 7.63 | 1.36 | 1.99E-05 | RHOBTB2 |
| TC0900012176.hg.1 | 7.06 | 7.53 | 1.39 | 1.69E-06 | URM1 |
| TC1100013027.hg.1 | 11.44 | 11.93 | 1.4 | 4.45E-08 | COX8A |
