# Supplementary material for: Hyperbaric oxygen therapy induces transcriptome changes in elderly: a prospective trial
Source: Aging (Albany NY). 2021 Nov 24;13(22):24511–23. doi: 10.18632/aging.203709 (PMC8660606; doi:10.18632/aging.203709)
Supplement: Supplementary Table 2 [file aging-13-203709-s002.pdf]

Supplementary Table

Supplementary Table 2. List of differentially expressed genes two weeks following the last HBOT session.

ID	Baseline Avg (log2)	2 weeks post HBOT Avg (log2)	Fold Change	FDR *P*-val
TC0700007480.hg.1	8.39	7.76	−1.54	0.0079
TC0600007092.hg.1	12.17	11.87	−1.23	0.0029
TC0300009147.hg.1	14.86	14.62	−1.18	0.0004
TC0300008355.hg.1	4.66	4.47	−1.14	0.0066
TC0300010643.hg.1	11.54	11.36	−1.14	0.008
TC1400010619.hg.1	14.01	13.84	−1.13	0.0027
TC0200013096.hg.1	13.78	13.67	−1.08	0.0024
TC0200014971.hg.1	13.56	13.47	−1.07	0.009
TC1300008837.hg.1	12.61	12.76	1.11	0.008
TC1100013037.hg.1	9.74	9.9	1.12	0.006
TC0900009164.hg.1	9.99	10.17	1.13	0.0058
TC1500007093.hg.1	10.92	11.1	1.14	0.009
TC0800011171.hg.1	12.39	12.59	1.15	0.0027
TC0700013591.hg.1	12.34	12.55	1.16	0.0051
TC0800012147.hg.1	11.28	11.51	1.17	0.0008
TC2000008230.hg.1	8.56	8.79	1.17	0.0058
TC0300007387.hg.1	9.39	9.66	1.2	0.0058
TC0600014111.hg.1	8.26	8.54	1.21	0.0004
TC0800007004.hg.1	7.19	7.48	1.22	0.0051
